# Mitochondrial Ca^2+^ uniporter (MCU) variants form plasma-membrane channels

**DOI:** 10.1038/s42003-026-10285-x

**Published:** 2026-05-20

**Authors:** Iuliia Polina, Jyotsna Mishra, Michael W. Cypress, Maria Landherr, Nedyalka Valkov, Isabel Chaput, Bridget Nieto, Brian Rhee, Ameneh Ahrari, Nathan DeMichaelis, Kye-Im Jeon, Ulrike Mende, Peng Zhang, Bong Sook Jhun, Jin O-Uchi

**Affiliations:** 1 Lillehei Heart Institute, Cardiovascular Division, Department of Medicine, University of Minnesota, Minneapolis, MN USA; 2 Department of Physiology, University of Arizona, Tucson, AZ USA; 3 Center for Translational Medicine, Department of Medicine, Thomas Jefferson University, Philadelphia, PA USA; 4 Department of Medicine, Brown University Health and Warren Alpert Medical School of Brown University, Providence, RI USA; 5 Heart Institute, Hypertension and Kidney Research Center, Department of Molecular Pharmacology and Physiology, University of South Florida Morsani College of Medicine, Tampa, FL USA; 6 Vascular Research Laboratory, Providence VA Medical Center, Providence, RI USA

**Keywords:** Calcium signalling, Mitochondria

## Abstract

*MCU*, originally known as *CCDC109A*, is widely recognized as the gene responsible for encoding a pore-forming subunit of a Ca^2+^-selective channel, mitochondrial Ca^2+^ uniporter complex (mtCUC). While MCU expression is typically highly mitochondrial-specific, we report here a protein variant derived from the *MCU* gene, termed MCU-S, which lacks the mitochondria-targeting sequence (MTS) and forms a Ca^2+^-permeable channel outside of mitochondria. The mRNA of *MCU-S* was ubiquitously expressed in all cell types/tissues tested, with particularly high expression in human platelets. MCU-S protein formed Ca^2+^ channels at the plasma membrane, which exhibited similar channel properties to those observed in mtCUC. MCU-S channels at the plasma membrane served as an additional Ca^2+^ influx pathway for platelet activation. Our findings show that the MCU-S functions are completely distinct from the originally reported functions of the *MCU* gene and provide additional insights into the molecular importance of MCU variant-dependent cellular Ca^2+^ handling.

**Figure:**
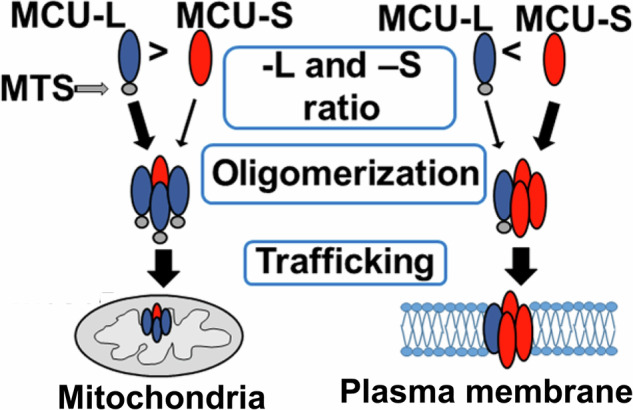

## Abstract (web summary)

While the mitochondrial Ca^2+^ uniporter (MCU) is typically recognized as a mitochondria-specific protein, MCU-S is a shorter isoform that forms Ca^2+^ channels at the plasma membrane. In platelets, MCU-S can serve as an additional Ca2+ influx pathway for platelet activation, suggesting a distinct function from the canonical function of full-length MCU.

## Introduction

The molecular identity of the pore-forming subunit of the mitochondrial Ca^2+^ (mtCa^2+^) uniporter complex (mtCUC) was discovered in 2011^1,2^, and provided opportunities for applying genetic approaches to precisely understand the mtCa^2+^ handling mechanism and its impact on the mitochondrial and cellular functions. The responsible gene coding the pore-forming protein was originally known as *CCDC109A* but has been renamed as *MCU* (Table 1). Indeed, rapid progress was attained in characterizing the role of MCU protein via genetic knockout/overexpression studies of *MCU* in both cell systems and in vivo^3–5^. Although the alteration of mtCa^2+^ via MCU is frequently observed in human diseases with disrupted energy metabolism^6–8^, it is still not clear how MCU protein expression and mtCUC function are impacted by the transcriptional regulation of *MCU* under both physiological and pathological conditions. MCU was originally considered to be a highly mitochondria-specific and Ca^2+^-selective channel in the inner mitochondrial membrane (IMM), with other subunits, including MICU family members, EMRE, and MCUb^3–5^, mainly because the original/conventional MCU protein (“long form” MCU, here renamed MCU-L) possesses a predicted cleavable N-terminal mitochondrial-targeting sequence (MTS)^1,2^ (see Table 1 and Supplementary Table 1). Data sets published prior to 2011 had predicted the existence of additional, still uncharacterized human and mouse MCU cDNAs^9–12^. However, the expression patterns and functional roles of these potential *MCU* variants have not yet been reported. Here, we report that the *MCU* (*CCDC109A*) gene encodes ion channels that are located both inside and outside the mitochondria. Especially in human platelets, an MCU-S variant forms a Ca^2+^-permeable channel at the plasma membrane (PM). This discovery greatly expands and revises the established concept of MCU^1,2^ as a mitochondria-specific Ca^2+^ channel.

**Table 1:** Gene names, variants, and corresponding protein names used in this study

Species	Gene name	Previous gene name	Variants, Transcript ID	Coding proteins UniProtKB ID
Human	*MCU*	*CCDC109A*	*MCU-L* (Baughman et al.^1^) ENST00000373053.8	MCU-L Q8NE86-1
			MCU-L2 ENST00000357157.10	*MCU-L2* Q8IQ70-2
			*MCU-S* ENST00000536019.5	MCU-S Q8NE86-3
Mouse	*Mcu*	*Ccdc109A*	*Mcu-L* (De Stefani et al. ^2^) ENSMUST00000020312.13	MCU-L Q3UMR5-1
			*Mcu-SS* ENSMUST00000009791.7	MCU-SS Q3UMR5-2

The information for human *MCU* and mouse *Mcu* genes and variants information was sourced from the Ensembl database ^46^ and UniProt (https://www.uniprot.org/). The names renamed in this study are shown in underlined.

## Results

### Detection of *MCU/Mcu* transcript variants

**Fig. 1:**
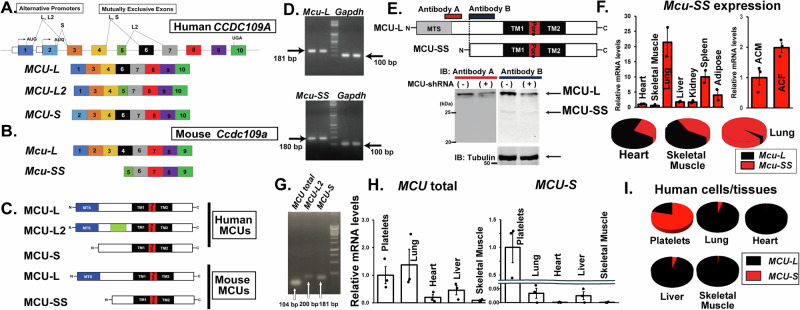
Detection of MCU variants in human and mouse cells/tissues. **A** Structural organization of predicted human *MCU* (*CCDC109A*) variants. The colored cassettes stand for the exons, and the blank cassettes show the predicted promoters. **B** Predicted mouse *Mcu* (*Ccdc109a*) variants. **C** Schematic protein structures of human and mouse MCU variants (see also Supplementary Fig 1). TM transmembrane domain. **D** Agarose gels showing PCR products of mouse *Mcu* variants. cDNA was created from mRNA extracted from C2C12 cells. The 1.8 kb fragments were amplified by a pair of *MCU* variant-specific primers. GAPDH was used as a control. The uncropped and unedited images are shown in Supplementary Fig 16. **E** *Top:* Mouse MCU-variant proteins and antibody detection sites. *Bottom:* Detection of MCU variant proteins in C2C12 cells overexpressing a control vector or shRNA targeted to the 3’ UTR of *Mcu*. The uncropped and unedited images are shown in Supplementary Fig 16. **F** *Top, left:* RT-qPCR analysis of *Mcu*-*SS* using adult FVB mouse tissues. Heart samples are from 4 mice, and all other organs are from 2 mice. Expression levels are normalized to the value in Heart. *Top, right:* RT-qPCR analysis of *Mcu-SS* using adult cardiomyocytes (ACMs) and cardiac fibroblasts (ACFs) isolated from 3 adult FVB hearts. Expression levels are normalized to ACMs. *Bottom:* Tissue-specific *Mcu-L*/*Mcu-SS* ratio in adult C56BL/6 mice calculated from the top, left panel. Error bars represent the SEM. **G** Agarose gel showing PCR products of human *MCU* variants using the total cDNA created from mRNA of HEK293T cells. The fragments were amplified using *MCU* variant-specific primers and a *MCU* total primer that reacts with all *MCU* variants. The uncropped and unedited image is shown in Supplementary Fig 16. **H** RT-qPCR analysis of total *MCU* and *MCU-S* in human tissues/cells. *n* = 3 for each group (see also Supplementary Tables 6 and 11). Expression levels are normalized to the value in platelets. Error bars represent the SEM. **I**. Tissue-specific *MCU-L/MCU-S* ratio in human platelets and tissues calculated from H.

*MCU* (in humans) and *Mcu* (in mice), originally known as *CCDC109A and Ccdc109A* genes (Table 1), consist of 10 and 9 exons, respectively (Fig. 1A and B). Based on a thorough search of online databases and published data sets of complete sequences for full-length, uncharacterized human and mouse cDNAs^9–12^, we identified 3 and 2 potential protein-coding transcripts from *CCDC109A* and *Ccdc109A* genes, respectively (Fig. 1A and B). Long-form mRNAs in both humans and mice (*MCU-L* and *Mcu-L*, respectively, see Table 1) are originally reported as the transcripts encoding the pore for mtCUC^1,2^.

In humans, two variant mRNAs (termed *MCU-L* and *MCU-L2*, see Table 1) start with the same 5’ exon (exon 1), which encodes the whole component of MTS^1,2^ and differ by alternative splicing of exon 5 or 6. Additional levels of complexity in the regulation of *MCU* gene may come from the presence of two different gene promoters found in the Eukaryotic Promoter Database (i.e., alternative initiation^13,14^), one of which drives the transcription of *MCU-L* and *-L2* starting from exon 1 (Fig. 1A and Supplementary Fig. 1). Another promoter accounts for the transcription of the “short-form” variant (termed *MCU-S*), which starts with exon 2. *MCU-S* lacks an MTS since the amino acids of the predicted MTS are not present in exon 2 (Fig. 1A, Supplementary Fig. 1, and Supplementary Table 1).

In mice, we also found a candidate transcript that is shorter than human *MCU-S* mRNA (termed “*Mcu-SS”*, see Table 1) (Fig. 1B and C and Supplementary Fig. 1). By designing specific primers targeting each variant (see Supplementary Tables 2 and 3), the expression of *Mcu* mRNA variants was confirmed in mouse tissues using conventional reverse transcription PCR (RT-PCR), quantitative RT-PCR (RT-qPCR) (Fig. 1D), and Western blotting of protein lysates from mouse myoblast C2C12 cells stably overexpressing shRNA targeting the 3’ untranslated region (UTR) of *Ccdc109a* (Fig. 1E. See also Supplementary Tables 4 and 5). The RT-qPCR assay showed ubiquitous expression of *Mcu-SS* in all investigated tissues, with the highest expression in the lung and with various *Mcu-L/Mcu-SS* ratios among the different mouse tissues (Fig. 1F, *left and bottom*). We also separately extracted the mRNA from primary cardiomyocytes and cardiac fibroblasts isolated from adult mice and found that cardiac fibroblasts contained a significantly higher amount of *Mcu*-*SS* mRNA compared to cardiomyocytes (Fig. 1F, *right*).

We next determined the expression of *MCU* variant mRNAs in humans. The specific primers targeted for *MCU-L2* and *MCU-S* were designed, and their specificity was confirmed by sequencing the PCR products purified from conventional RT-PCR gels (Fig. 1G, Supplementary Table 2 and Supplementary Figs. 2 and 3). The design of specific primers targeting *MCU-L* was challenging due to its high similarity with other variants (Supplementary Fig. 2). Therefore, a primer pair targeting the pore region was used to detect all *MCU* variants (i.e., *MCU* total) (Fig. 1G, Supplementary Table 2 and Supplementary Fig. 2), and *MCU-L* mRNA amount was then estimated by subtraction of other variants from *MCU* total mRNA. The expression of *MCU-L2* mRNA was barely detectable by conventional PCR (Fig. 1G) and RT-qPCR in all tissues and cell lines investigated (Supplementary Fig. 4).

*MCU-S* mRNA expression was detected in all tested cells/tissues, with significantly higher expression in platelets (Fig. 1G-I). It is technically challenging to distinguish the human MCU-S protein from the mature MCU-L protein using conventional SDS-PAGE and Western blotting due to their similarity with only one amino acid difference (M and T, respectively, see Supplementary Fig. 1). Therefore, we alternatively examined the endogenous protein expression of MCU-SS in the platelets isolated from adult mice. We found a smaller-sized protein band that reacted with an antibody against the internal region of MCU-L, but not with the one against the N-terminal, suggesting that the short *MCU* mRNAs can produce stable proteins in primary cells (Supplementary Fig. 5).

In summary, we found *MCU/Mcu* variant mRNAs that may produce N-terminal truncated MCU proteins in addition to the originally reported MTS-containing MCU. Specifically, we detected the endogenous MCU-SS protein in mouse cells, including platelets.

### Short MCU variants form Ca^2+^ channels at the plasma membrane, but not in the mitochondria

**Fig. 2:**
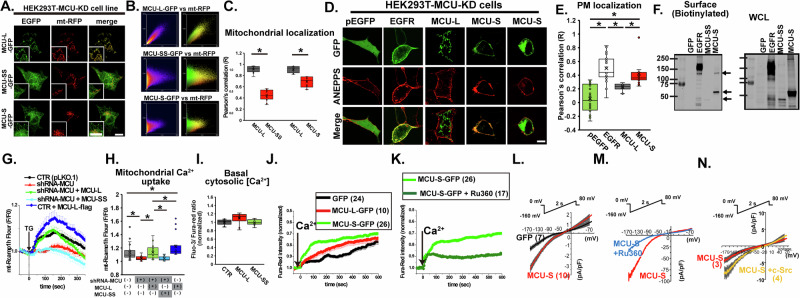
Short MCU variants do not serve as a mitochondrial Ca^2+^ channel, but form Ca^2+^ channels at the plasma membrane. **A** Subcellular localization of GFP-tagged MCU variants in MCU-KD cells. Scale bars, 10 µm. **B** Frequency (left) and color scatter plots (right) obtained from (**A**). **C** Summary data of Pearson’s correlation coefficient value from (**A**). The value of Pearson’s correlation coefficient nearly reached 1.0 when the subcellular localization of GFP and mt-RFP completely overlapped. *n*  (left to right) = 15,14, 12, and 10; 1st quartile (left to right), 0.914, 0.313, 0.928, and 0.570; mean averages (left to right), 0.941, 0.404, 0.946, and 0.665; median values (left to right), 0.952, 0.392, 0.951, and 0.708; 3rd quartile (left to right), 0.967, 0.525, 0.964, and 0.763; maximum values (left to right), 0.981, 0.554, 0.969, and 0.767; and minimum values (left to right), 0.863, 0.173, 0.915, and 0.401. Unpaired T-test: *p < 0.05. **D** PM localization of MCU-S-GFP in MCU-KD cells. Overexpression of GFP and EGFR-GFP was shown as negative and positive controls. Scale bar, 10 µm. **E**. Summary data of Pearson’s correlation coefficient value from (**D**). *n* (left to right) = 23 29, 35, and 19; 1st quartile (left to right), −0.110, 0.375, 0.180, and 0.330; mean averages (left to right), 0.103, 0.500, 0.254, and 0.421; median values (left to right), 0.070, 0.440, 0.250, and 0.360; 3^rd^ quartile (left to right), 0.290, 0.635, 0.340, and 0.440; maximum values (left to right), 0.81, 0.83, 0.39, and 0.48; and minimum values (left to right), -0.27, 0.07, 0.13, and 0.24. One-way ANOVA, Tukey’s post-hoc test: *p < 0.05. **F** Detection of MCU-S-GFP in the PM protein collected from HEK293Tcells stably overexpressing MCU-S-GFP by cell surface biotinylation. The PM proteins obtained from HEK293T cells stably overexpressing EGFR-GFP were used for positive control. Immunoreactive bands were detected by GFP antibody. In the PM, MCU-S-GFP was detected as monomer and dimer indicated by arrows. The uncropped and unedited image are shown in Supplementary Fig. 17. **G** mtCa^2+^ uptake triggered by the SERCA inhibitor 3 μM TG in MCU-KD cells after reintroduction of MCU variants. The changes in Ca^2+^ concentration at the mitochondrial matrix [Ca^2+^]_m_ were assessed by mitochondrial matrix-targeted Ca^2+^ biosensor mtRCaMP1h. **H** Summary data of G. One-way ANOVA, Tukey’s post-hoc test: *n* (left to right) = 28, 24, 28, 16, and 18; 1st quartile (left to right), 1.063, 1.024, 1.049, 1.019, and 1.118; mean averages (left to right), 1.128, 1.045, 1.133, 1.042, and 1.223; median values (left to right), 1.094, 1.035, 1.100, 1.026, and 1.149; 3rd quartile (left to right), 1.162, 1.070, 1.205, 1.069, and 1.235; maximum values (left to right), 1.292440164, 1.125472461, 1.384742273, 1.109080333, and 1.244951527; and minimum values (left to right), 1.018815722, 1.004118658, 1.027213791, 1.005034002, and 1.104975199. **I**. Basal [Ca^2+^]_c_ in cells stably overexpressing pcDNA3.1(+) (as a control), MCU-L, and MCU-S assessed by ratiometric [Ca^2+^] measurement using Ca^2+^-sensitive dyes Fluo-3 and Fura-Red. *n* = 10 for each group; 1st quartile  (left to right), 0.952, 0.988, and 0.955; mean averages (left to right), 1.000, 1.094, and 0.997; median values (left to right), 1.011, 1.143, and 0.988; 3rd quartile (left to right), 1.053, 1.186, and 1.063; maximum values (left to right), 1.076497051, 1.221544852, and 1.0929661; and minimum values (left to right), 0.887604095, 0.8354, and 0.874396428. **J** Changes in [Ca^2+^]_c_ in response to re-admission of 2 mM Ca^2+^ (arrow) following short-time removal of the extracellular Ca^2+^ assessed by Fura-Red. Error bars represent the SEM. **K** Effect of 1 μM Ru360 on cells overexpressing MCU-S-GFP. **L** Whole-cell *I*_*ca*_ elicited by voltage ramps in HEK293T stably expressing GFP and MCU-S-GFP. Error bars represent the SEM. **M** Representative recording of *I*_*Ca*_ before and after 1 μM Ru360 bath application. Error bars represent the SEM. **N** Co-expression of constitutively active c-Src enhances whole-cell *I*_*Ca*_ in HEK293T stably expressing MCU-S-GFP. Error bars represent the SEM.

Since the first exon for *MCU-L* mRNA, which includes MTS, is not used in mRNAs for human *MCU-S* or mouse *Mcu-SS* (Fig. 1A and B), both isoforms are predicted to produce proteins that have a significantly lower probability for being transported into the mitochondria compared to MCU-L proteins, as determined by mitochondrial-transport prediction programs (see Supplementary Table 1). Therefore, we examined whether MCU-S may be transported into other organelles and/or membrane compartments, such as PM, rather than mitochondrial membranes. To assess the subcellular localizations of MCU-S/MCU-SS and their functions in cellular Ca^2+^ handling, we reintroduced a single MCU variant with a C-terminal tag of GFP or Flag into the MCU knock-down (MCU-KD) HEK293T cell line stably overexpressing shRNA plasmids targeting the 3’-UTR of *MCU* mRNA^1^ (Supplementary Fig. 6). Live-cell imaging using the mitochondrial matrix-targeted DsRed (mt-RFP)^15,16^ revealed that MCU-L-GFP is exclusively localized in the mitochondria, as previously reported^1,2,15^ (Fig. 2A-C). In contrast, both MCU-S and MCU-SS showed significantly lower co-localization with mt-RFP compared to MCU-L, indicating that these isoforms are distributed at extramitochondrial sites, although a small fraction is still detectable in the mitochondria (Fig. 2A-C). Live cell imaging using a PM potential sensitive-dye, Di-8-ANEPPS^17^ showed that MCU-S/MCU-SS, but not MCU-L, are expressed at the PM area (Fig. 2D and E). A surface protein biotinylation assay^18^ further demonstrated that MCU-S and -SS are expressed on the extracellular face of the PM (Fig. 2F). Overexpressed GFP-tagged MCU-S and -SS showed variable levels of intracellular retention in addition to PM localization (Fig. 2A and D and Supplementary Fig. 6C), which is unlikely to be due to protein aggregation (Supplementary Fig. 7).

Reintroduction of MCU-L into MCU-KD cells successfully restored mtCa^2+^ uptake in response to the elevation of cytosolic Ca^2+^ concentration ([Ca^2+^]_c_) by thapsigargin (TG), a sarco/endoplasmic reticulum (SR/ER) Ca^2+^-ATPase (SERCA) inhibitor^16^, close to the levels in control cells (Fig. 2G and H). However, MCU variant expression failed to rescue the impaired mtCa²⁺ uptake in MCU-KD cells (Fig. 2G and H). Next, to test whether short MCU variants form a functional Ca^2+^-permeable channel at the PM, the changes in [Ca^2+^]_c_ were monitored after re-administration of extracellular Ca^2+^ following its short-time removal. Resting [Ca^2+^]_c_ was not significantly different between MCU-L and MCU-SS overexpressing cells (Fig. 2I). Upon increase in extracellular Ca^2+^ concentration from 0 to 2 mM, cells expressing short MCUs exhibited a more rapid increase in [Ca^2+^]_c_ compared to cells expressing GFP or MCU-L-GFP (Fig. 2J and Supplementary Fig. 8). Importantly, Ca^2+^ influx via MCU-S was significantly inhibited by a 5-min pretreatment with a cell-impermeable MCU inhibitor Ru360 in the extracellular solution (Fig. 2K). In contrast, Ru360 did not show any significant effect on the [Ca^2+^]_c_ profile in control cells (Supplementary Fig. 9).

Next, to precisely characterize the mtCUC-like channel function formed by MCU-S at the PM, whole-cell Ca^2+^ current (*I*_*Ca*_) was measured using a protocol adapted from mitoplast patch-clamp recordings of mtCUC activity^19^. We recorded Ru360-sensitive whole-cell *I*_*Ca*_ elicited by voltage ramps, which resembles the whole-IMM mtCUC current observed in mitoplasts^19^ (Fig. 2L and M). Finally, to further investigate the topology of MCU-S at the PM, constitutively active c-Src was co-expressed since c-Src activates mtCUC via tyrosine phosphorylation of MCU-L termini located in the mitochondrial matrix^20^. Larger *I*_*Ca*_ was recorded when constitutively active c-Src was co-expressed (Fig. 2N), indicating that the N- and C-termini of PM-localized MCU-S face the cytosol.

In summary, short MCU variants can form Ru360-sensitive Ca^2+^-permeable channels at the PM rather than the IMM.

### Short MCU variant inhibits mitochondrial Ca^2+^ uptake

**Fig. 3:**
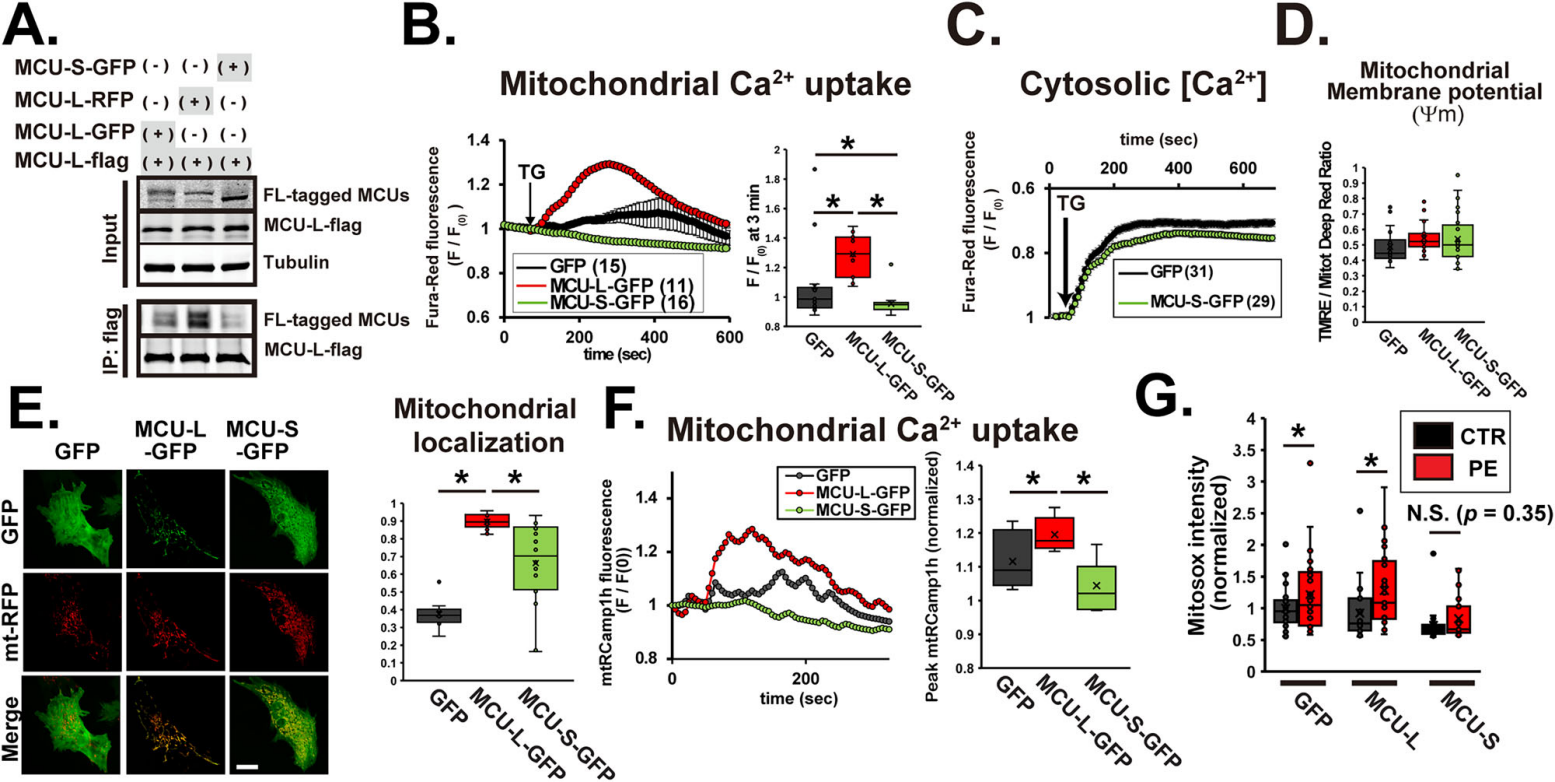
MCU-S inhibits mitochondrial Ca^2+^ uptake in cardiomyocytes. **A** Co-immunoprecipitation analysis of MCU-L and MCU-S. HEK293T cells stably overexpressing MCU-L-flag were transiently transfected with fluorescence (FL)-tagged MCU-L or MCU-S, and the whole cell lysates were prepared. Co-immunoprecipitation was performed with an anti-Flag antibody, and MCU was detected by antibody A used in Fig. 1E. The uncropped and unedited image is shown in Supplementary Fig. 17. **B** *Left:* mtCa^2+^ uptake triggered by thapsigargin (TG) assessed by mtRCaMP1h. *Light:* Summary data. *n* (left to right) = 15, 11, and 16 cells collected from 3 independent littermates; 1st quartile (left to right), 0.927, 1.134, and 0.916; mean averages (left to right), 1.073, 1.292, and 0.954; median values (left to right), 0.986, 1.293, and 0.948; 3^rd^ quartile (left to right), 1.064, 1.407, and 0.962; maximum values (left to right), 1.087426103, 1.478588983, and 0.976740864; and minimum values (left to right), 0.876823554, 1.072435194, and 0.875971688. One-way ANOVA, Tukey’s post-hoc test: *p < 0.05. **C** ER Ca^2+^ estimated by the cytosolic Ca^2+^ elevation levels by TG treatment, measured by a Ca^2+^-sensitive dye Fura-Red. **D** Effect of MCU-S overexpression on resting *Ψ*_*m*_. TMRE is a *Ψ*_*m*_-sensitive dye, and MtDRFM can label both active and inactive mitochondria, which does not depend on *Ψ*_*m*_. TMRE/MtDRFM ratio was used for comparison. *n* (left to right) = 18, 23, and 29 cells collected from 3 independent littermates; 1st quartile (left to right), 0.413, 0.488, and 0.425; mean averages (left to right), 0.488, 0.544, and 0.539; median values (left to right), 0.446, 0.522, and 0.500; 3rd quartile (left to right), 0.532, 0.574, and 0.628; maximum values (left to right), 0.62527457, 0.661429028, and 0.853427609; and minimum values (left to right), 0.352558291, 0.404305727, and 0.341037379. **E** *Left:* Subcellular localization of GFP-tagged MCU variants in rat NCMs. EGFP was used as a control. Mitochondria were labeled by mt-RFP. Scale bar, 20 µm. *Right:* Summary data of Pearson’s correlation coefficient values. *n* (left to right) = 10, 13, and 18 cells collected from 3 independent littermates; 1st quartile (left to right), 0.329, 0.868, and 0.513; Mean averages (left to right), 0.383, 0.903, and 0.644; median values (left to right), 0.369, 0.895, and 0.703; 3^rd^ quartile (left to right), 0.403, 0.936, and 0.866; maximum values (left to right), 0.422, 0.958, and 0.932; and minimum values, (left to right) 0.25, 0.825, and 0.165. One-way ANOVA, Tukey’s post-hoc test: *p < 0.05. **F** *Left:* Representative traces of mtCa^2+^ uptake triggered by TG. [Ca^2+^]_m_ was measured by mtRCaMP1h. *Right:* summary data. *n* (left to right) = 19, 6, and 10 cells collected from 2 independent littermates; 1st quartile (left to right), 1.044, 1.159, and 1.027; mean averages (left to right), 1.117, 1.210, and 1.057; median values (left to right), 1.119, 1.195, and 1.043; 3rd quartile (left to right), 1.201, 1.284, and 1.123; maximum values (left to right), 1.285, 1.284, and 1.132; and minimum values (left to right), 1.01885068, 1.146154655, and 1.019052476. One-way ANOVA, Tukey’s post-hoc test: *p < 0.05. **G** Effect of G_q_-coupled α_1_-adrenoceptor stimulation by phenylephrine (PE, 100 μM, 0.5 hr) on mitochondrial superoxide levels in NCMs overexpressing MCU variants assessed by MitoSOX-Red. PE stimulation was used for inducing mitochondrial superoxide generation via mtCa^2+^ overload in NCMs. NCMs overexpressing MCU-S-GFP have lower mitochondrial superoxide at rest than those overexpressing EGFP or MCU-L-GFP. NCMs overexpressing MCU-S-GFP did not show a significant increase in mitochondrial superoxide after PE stimulation. *n* (left to right) = 28, 28, 25, 26, 17 and 19 cells collected from 3 independent littermates; 1st quartile (left to right), 0.780, 0.723, 0.650, 0.832, 0.596, and 0.617; mean averages (left to right), 1.009, 1.214, 0.925, 1.281, 0.732, and 0.836; median values (left to right), 0.951, 1.048, 0.758, 1.089, 0.631, and 0.668; 3rd quartile (left to right), 1.126, 1.569, 1.157, 1.746, 0.739, and 1.031; maximum values (left to right), 1.536440972, 2.284058503, 1.562836129, 2.90739233, 0.882853471, and 1.625774721; and minimum values (left to right), 0.556185649, 0.580931964, 0.54961588, 0.591977288, 0.548741703, and 0.571559715. One-way ANOVA, Tukey’s post-hoc test: *p < 0.05. N.S., not significant.

Since the MCU-S protein includes all key structures that are required for maintaining channel activity in MCU-L^1,2^ (see Fig. 1 and Supplementary Fig. 1), we next investigated whether MCU-S or MCU-SS can interact with MCU-L and affect conventional MCU function. Co-immunoprecipitation of heterologously co-expressed MCUs with different tags^16,21^ and Förster resonance energy transfer (FRET)-based assessments in live HEK293T cells^22^ suggest an interaction between MCU-L and MCU-S/SS, in addition to the MCU-L-MCU-L binding (Fig. 3A and Supplementary Fig. 10). Mitochondrial fractionation assay showed that overexpressed MCU-S-GFP in HEK293T cells is mainly found in the cytosolic fraction (Supplementary Fig. 11), which contains the ER and PM^15,16^. In MCU-S-GFP ( ~ 55 kDa)-overexpressing HEK293T cells, endogenous MCU-L expression in the mitochondria-enriched fraction (the ~32-kDa bands in Supplementary Fig. 11A) was markedly decreased compared to control cells, concomitant with the appearance of a ~ 40-kDa band (corresponding to the size of endogenous MCU-L with MTS) in the cytosolic fraction (Supplementary Fig. 11A). Corresponding to the decreased MCU-L in the mitochondria (Supplementary Fig. 11A and B), MCU-S-GFP overexpression decreased mtCa^2+^ uptake in response to TG application, without affecting the ER Ca^2+^ content or the mitochondrial membrane potential (*Ψ*_*m*_) (Fig. 3B-D). This observation is consistent with overexpression studies of dominant-negative (DN) pore-forming subunits of MCU-L (MCU-DN)^1,2,16^ or endogenous MCU-inhibitory subunit MCUb^16,21^, which binds to MCU-L and forms Ca^2+^-impermeable pores, but likely using a different molecular mechanism (i.e., decreasing the mtCUC number in the mitochondria, see Supplementary Fig. 11C). Similar results were observed in the primary cells. Overexpressed MCU-S was found outside of mitochondria, which can inhibit mtCa^2+^ uptake and mtCa^2+^ uptake-mediated mitochondrial superoxide generation in rat neonatal cardiomyocytes (NCMs) (Fig. 3E-G).

In summary, in the presence of MCU-L, MCU-S can form hetero-oligomers with MCU-L, interfering with MCU-L trafficking to the IMM, and subsequently inhibiting mtCa^2+^ uptake.

### MCU-S at the plasma membrane forms a Ca^2+^-permeable pathway in human platelets

**Fig. 4:**
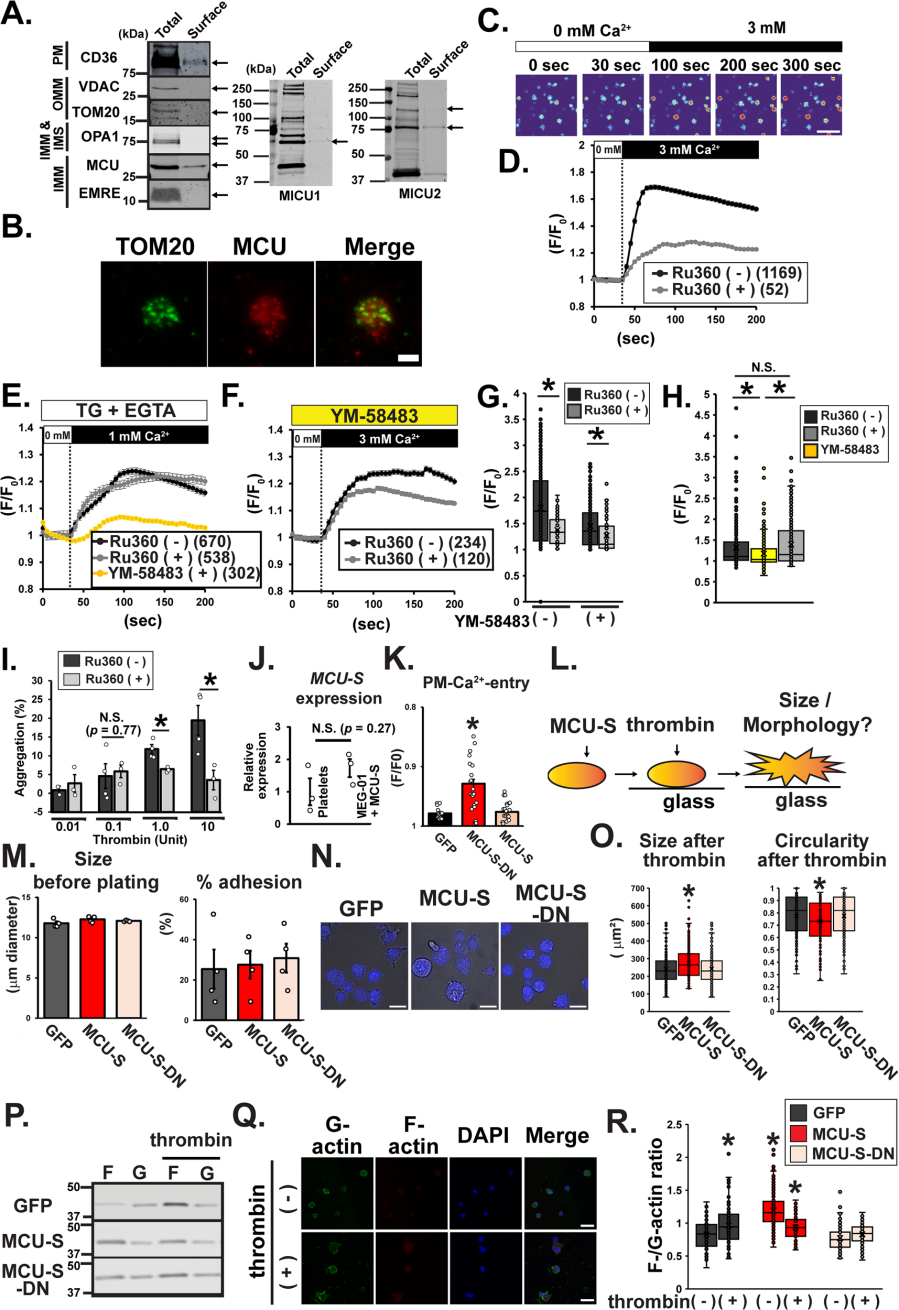
Ca^2+^-permeable MCU channels at the plasma membrane participate in cellular Ca^2+^ signaling in human platelets. **A** The PM localization of MCU and mtCUC subunits by cell surface biotinylation assay followed by immunoblots. CD36 was used as a positive control for PM proteins in platelets. OMM, outer mitochondrial membrane; IMS, intermembrane space; VDAC, voltage-dependent anion channel; OPA1, optic atrophy 1. The uncropped and unedited image are shown in Supplementary Fig. 18. **B** Representative confocal images of fixed platelets co-stained with anti-TOM20 and an MCU-specific antibody against the N-terminal domain (see also Supplementary Fig. 12). Scale bar, 2 μm. **C** Representative time-lapse confocal images of the changes in [Ca^2+^]_cyto_ in response to the reintroduction of extracellular Ca^2+^ (3 mM) after following the 1 min removal of extracellular Ca^2+^, assessed by Fluo-3. Scale bar, 10 µm. **D** Effect of 10 μM Ru360 on Ca^2+^ entry via the PM. **E** Effect of 10 μM Ru360 and a SOCE inhibitor (3 μM YM-58483) on SOCE. For ER Ca^2+^ depletion, platelets were pretreated with 1 μm TG and 200 μm EGTA for 5 min. **F** Non-SOCE Ca^2+^ entry via the PM in the presence or absence of Ru360. **G** Summary data of (**D** and **F**). *n* (left to right) = 1169, 52, 234, and 120; 1st quartile (left to right), 1.171, 1.117, 1.093, and 1.026; mean averages (left to right), 1.809, 1.376, 1.451, and 1.267; median values (left to right), 1.743, 1.332, 1.356, and 1.103; 3rd quartile (left to right), 2.324, 1.574, 1.705, and 1.449; maximum values (left to right), 3.97404485, 2.04346073, 2.592620555, and 1.952978693; and minimum values (left to right), 1.005146944, 1.011958, and 1, 1.007991. Unpaired T-test: *p < 0.05. **H** Summary data of E. *n* (left to right) = 670, 302, and 535; 1st quartile (left to right), 1.017, 0.974, and 1.001; mean averages (left to right), 1.323, 1.178, and 1.395; median values (left to right), 1.105, 1.036, and 1.154; 3rd quartile (left to right), 1.456, 1.292, and 1.725; maximum values (left to right), 2.111720668, 1.763687015, and 1.725442884; and minimum values (left to right), 0.779026247, 0.652370587, and 0.871856161. One-way ANOVA, Tukey’s post-hoc test: *p < 0.05. **I** Effect of Ru360 on thrombin-mediated platelet aggregation assessed by the microplate-based light transmission aggregometry in the presence or absence of 3 µM Ru360. Human platelets were stimulated with thrombin at indicated concentrations. *n* = 2, 3, 4, 3, 4, 3, 4, and 3 (left to right). Unpaired T-test were performed for the experiments with ≥ 0.1 U of thrombin. *p < 0.05, compared to control cells treated with the vehicle (Ru360(-)) in each thrombin concentration. Error bars represent the SEM. **J** RT-qPCR analysis of *MCU-S* in human platelets (Plt) and MEG-01 transfected with an MCU-S plasmid assessed by MCU-S and GFP primers, respectively. *n* = 3 for each group. The mRNA samples from MEG-01 were treated with DNase to remove MCU-S plasmid DNA. Error bars represent the SEM. **K** Effect of MCU-S or MCU-S-DN expression on Ca^2+^ entry via the PM in MEG-01 cells assessed by Fura-Red. See protocol in panel 4 C. One-way ANOVA, Tukey’s post-hoc test: *n* = 15-21, *p < 0.05, compared to GFP-transfected cells. Error bars represent the SEM. **L** Schematic diagram of experimental setup. Non-adherent MEG-01 cells were collected and plated on glass-bottom dishes for 20 min. Adherent cells were loaded with a cell-permeant fluorescent probe, Calcein Violet-AM, for 20 min to confirm their viability and then stimulated with thrombin (0.1 U/mL) for 30 min. **M** Effect of MCU-S on cell size (before plating) and adhesion to glass dishes. *n* = 4 for each group. **N** Representative confocal and phase contrast images of MEG-01 cells transfected with GFP, MCU-S-GFP, and MCU-S-DN-GFP. Scale bars, 20 μm. **O** Effect of thrombin on cell size and circularity after plating on glass-bottom micro dishes assessed by phase contrast images. Circularity reaches 1 if the cell is a perfect circle, and circularity becomes low in complex cell shapes. *n* (left to right) = 176, 217, 176, 176, 217, and 176; 1st quartile (left to right), 182.884, 205.973, 182.884, 0.655, 0.611, and 0.655; mean averages (left to right), 241.896, 275.340, 241.896, 0.776, 0.730, and 0.776; median values (left to right), 230.499, 264.874, 230.499, 0.819, 0.732, and 0.819; 3rd quartile (left to right), 287.887, 328.002, 287.887, 0.928, 0.879, and 0.928; maximum values (left to right), 445.375, 500.35, 445.375, 1, 0.966, 1; and minimum values (left to right), 81.897, 130.591, 81.897, 0.306, 0.251, and 0.306. One-way ANOVA, Tukey’s post-hoc test. *p < 0.05, compared to GFP-transfected cells. **P** Representative immunoblots of F-/G-actin in MCU-S-GFP transfected MEG-01 cells before and after thrombin stimulation. GFP- and MCU-S-DN-GFP-transfected cells are shown as comparisons. The uncropped and unedited image is shown in Supplementary Fig. 18. **Q** Representative confocal images of fixed GFP-transfected MEG-01 cells before and after thrombin stimulation, co-stained with monoclonal JLA20 anti-actin antibody and CF568 conjugated-phalloidin to detect G-actin and F-actin, respectively. Scale bars, 20 μm. **R** Summary data of F-/G-actin ratio quantified from confocal images. *n* (left to right) = 74, 157, 295, 48, 101, and 145; 1st quartile (left to right), 0.649, 0.756, 1.023, 0.799, 0.634, and 0.730; mean averages (left to right), 0.829, 0.964, 1.191, 0.933, 0.770, and 0.831; median values (left to right), 0.833, 0.944, 1.159, 0.932, 0.749, and 0.841; 3rd quartile (left to right), 0.978, 1.135, 1.331, 1.054, 0.863, and 0.941; maximum values (left to right), 1.322, 2.051, 1.789676371, 1.350, 1.158, and 1.163; and minimum values (left to right), 0.321657099, 0.45773226, 0.636859886, 0.59213142, 0.465098612, and 0.438734896. One-way ANOVA, Tukey’s post-hoc test. *p < 0.05, compared to GFP-transfected cells before thrombin stimulation.

Since *MCU-S* mRNA was expressed most abundantly in platelets among the human cells/tissues that we tested (see Fig. 1), we next addressed whether endogenous MCU-S proteins can form Ca^2+^ channels at the PM in human platelets from healthy donors (Supplementary Tables 6) similar to our observation in the heterologous expressions system (see Fig. 2), and if so, whether this Ca^2+^ pathway has a role in cellular Ca^2+^ signaling and overall platelet functions. First, three established subunits of mtCUC, MCU, and MICU1/2were found in the PM proteins collected via a cell surface biotinylation assay^18^, but EMRE was not detectable (Fig. 4A). Next, the mitochondrial and extra-mitochondrial localization of MCU was confirmed via immunocytochemistry (Fig. 4B, see also Supplementary Fig. 12). Lastly, similar to HEK293T cells stably overexpressing MCU-S, human platelets exhibited Ru360-sensitive [Ca^2+^]_c_ elevation upon an increase of extracellular [Ca^2+^] from 0 to 3 mM (Fig. 4C and D). A previous report showed that 5-min pretreatment of an inhibitor of store-operated calcium entry (SOCE), YM-58483 (0.1–10 μM), blocked SOCE in a concentration-dependent manner in the human platelets, with a half-maximal inhibition (*IC*_*50*_) of 0.5 μM and the complete inhibition at 3 μM of YM-58483^23^. Moreover, non-capacitive Ca^2+^ entry activated by a diacylglycerol analog was not affected by 3 μM of YM-58483^23^. Even under the SOCE inhibition by 3 μM YM-58483, we found that the Ru360-sensitive Ca^2+^ influx component still existed (Fig. 4F and G). Although Ru360 did not inhibit SOCE in the platelets (Fig. 4E and H), 5-min pretreatment of Ru360 significantly inhibited platelet aggregation induced by ≥ 1-Unit (U) thrombin (Fig. 4I).

In summary, human platelets possess an endogenous Ru360-sensitve Ca^2+^-permeable pathway at the PM that is an independent mechanism from SOCE, which may potentially modulate platelet functions such as aggregation.

Since in vitro gene modification is challenging in platelets due to the lack of nuclei, we next utilized a human megakaryocyte leukemia cell line (MEG-01) to further assess the impact of MCU-S expression on the platelet Ca^2+^ signaling and functions. Importantly, *MCU-S* mRNA was barely detectable in MEG-01 cells, and the conventional protocol for the induction of in vitro differentiation/maturation^24^, which leads to polyploidy and the production of platelet-like particles, failed to induce the *MCU-S* expression in MEG-01 cells, indicating that MCU-S expression is likely specific to the matured platelets in vivo (Supplementary Fig. 13). Therefore, we used this cell line as a template for exogenously expressing MCU-S and its dominant-negative (DN) mutant MCU-S-DN (mutating negatively charged residues of the conserved region in the MCU-S pore [D260, E263 in MCU-L^2,16^] into glutamines). We titrated the overexpression levels of *MCU-S* in MEG-01 cells comparable to the levels of endogenous *MCU-S* in human platelets (Fig. 4J). Introducing MCU-S, but not MCU-S-DN, increased PM-Ca^2+^ entry, as we observed in the HEK293T system (Fig. 4K). Platelets undergo morphological changes via actin polymerization when [Ca^2+^]_c_ elevation occurs, such as ER-Ca^2+^ release by thrombin stimulation, one of the key inducers of platelet activation in vivo via protease-activated receptors^25^. Following the published protocol^26^, we next plated non-adherent MEG-01 cells to adhere to glass-bottom microplates and then treated them with thrombin to activate downstream Ca^2+^ signaling (Fig. 4L). MCU-S or MCU-S-DN expression did not affect the cell size before plating, adhesion efficiency (Fig. 4M), or the expression of cellular Ca^2+^ handling proteins, including SOCE machinery proteins, ER-Ca^2+^ handling proteins, mtCa^2+^ handling proteins, and conventional PM-Ca^2+^ entry pathways in platelets (Supplementary Fig. 14, and Supplementary Tables 7 and 8). We also observed that morphological changes induced by thrombin treatment in MEG-01 cells were not affected by the pretreatment with a mitochondrial uncoupler, carbonyl cyanide-p-trifluoromethoxyphenylhydrazone (FCCP) that can eliminate the mtCa^2+^ uptake (Supplementary Fig. 15A and B). This indicates that 1) the Ca^2+^ release via mitochondrial permeability transition pore (mPTP) triggered by the mtCa^2+^ uptake is likely not required for actin activation and 2) [Ca^2+^]_c_ elevation by ERCa^2+^ release is sufficient to cause morphological changes. Importantly, MCU-S transfected cells showed increased cell size and decreased circularity after thrombin stimulation compared to GFP (as a control) and MCU-S-DN transfected cells (Fig. 4N and O). Moreover, this effect by MCU-S was enhanced by the FCCP pretreatment, possibly due to the decreased [Ca^2+^]_c_ buffering by mitochondria that exaggerates the [Ca^2+^]_c_ elevation (Supplementary Fig. 15C). We confirmed that thrombin increased β-actin polymerization (as assessed by increased filamentous [F]-actin /globular [G]-actin ratio, see also Material and Methods) in control cells, whereas in MCU-S-transfected cells, the F-/G-actin ratio was already elevated without thrombin stimulation (Fig. 4P-R). Furthermore, thrombin-induced β-actin polymerization was abolished by MCU-S-DN transfection (Fig. 4P-R).

In summary, endogenous MCU proteins in human platelets, likely MCU-S, form Ca^2+^ channels at the PM. The Ca^2+^ entry via PM-MCU-S channels activates Ca^2+^ signaling for actin rearrangements after global [Ca^2+^]_c_ elevation by thrombin, which is critical for the activation of aggregation/coagulation pathways in platelets^27,28^.

## Discussion

MCU is dogmatically known as a pore subunit for the highly Ca^2+^-selective channel at the IMM and represents the major mechanism for mtCa^2+^ uptake. This is mainly because 1) the MTS found in the conventional MCU-L variant^1,2^ (see also Supplementary Fig.1. and Supplementary Table 1) and 2) mitochondria mass spectrometry data support MCU-L protein expression in the mitochondria^29^. Here, we report that short MCU variants that do not possess an MTS (Fig. 1) are likely to form the MCU tetramers outside of mitochondria and can produce mtCUC-like Ca^2+^-permeable channels at the PM in the heterologous expression system and in human platelets (Figs. 2 and 4).

The human mtCUC contains 4 or 5 major subunits: 1) MCU-L, which tetramerizes to form the Ca^2+^-permeable pore for mtCUC; 2) EMRE, a single-pass transmembrane protein that activates metazoan MCU channel activity in the IMM environment; and 3) MICU family members that provide Ca^2+^-sensitivity to the mtCUC^3–5^.

First, our biochemical and cell biological data suggests that MCU-L can interact with MCU-S (Fig. 3A and Supplementary Fig. 10). Since matured MCU-S and MCU-L have only one amino acid difference at the N-terminal (Supplementary Fig.1), it is feasible that MCU-S and MCU-L can form hetero-oligomers via similar domain interactions as those reported in MCU-L-MCU-L or MCU-L-MCUb^22,30^, which contribute to the extra-mitochondrial retention of MCU heteromers when MCU-S is overexpressed (Fig. 3 and Supplementary Fig. 11). Moreover, MCU-S is likely to form homomeric pore structures because MCU-S has all the key structures that are required to form stable MCU-L tetramers (*i.e*., N-terminal domain, coiled-coil domain 1, and linking helix α1 between these two domains^30^, see also Supplementary Fig. 1). Indeed, we detected *I*_*Ca, MCU*_-like current after MCU-S overexpression at the PM of HEK293T cells, which is a similar observation to the recent report from Tsai’s group using MCU-L-EMRE tandem construct in the Xenopus oocytes^31^.

Second, while EMRE is recognized as a key protein for mtCUC assembly and Ca^2+^ transport activity in the IMM in higher organisms^32^, our biochemical assay failed to detect the endogenous EMRE in the PM of human platelets (Fig. 4A). Several reports showed that the human MCU protein alone is sufficient for MCU activity in artificial bilayers^2,21,33^. Since the lipid composition/environment is different between the IMM and PM, MCU-S oligomers may produce Ca^2+^ permeability without interacting with EMRE in the PM, similar to the case in artificial bilayers. Another possibility is that mammalian PM may contain small single-pass membrane proteins like EMRE that can alternatively activate the MCU pores.

Third, although PM fractions from human platelets contain endogenous MICU1 and MICU2 (Fig. 4A), it remains unclear whether they can regulate endogenous MCU-S pore functions since PM-localized MCU-S channels are continuously exposed to Ca^2+^ concentrations in the millimolar range, which is ≅1000 times higher than the *K*_*d*_ for Ca^2+^ of the regulatory MICU complex^34^. Indeed, the expression of the MICU subunits at the PM is entirely omitted to record whole-cell *I*_*Ca, MCU*_ in our heterologous expression system study (Fig. 2L-M), as well as in the report from Tsai’s group using Xenopus oocytes^31^, suggesting that the interaction between MCU-S and the MICU subunits is unlikely required to produce *I*_*Ca, MCU*_-like current at the PM. Moreover, since the interaction between the MICU subunits and MCU-L is suggested to occur via EMRE^35^, MCU-S at the PM may not interact with MICU subunits due the lack of (or low expression of) EMRE at the PM in human platelets.

Nevertheless, given that EMRE and MICUs exert significant influence on intrinsic channel activity, and/or channel complex assembly in the IMM^3–5^, the detailed channel properties of mtCUC at the IMM and MCU-S channels at the PM, such as single channel conductance and the Ca^2+^-sensitivity, are likely different. However, our data using MCU-S-DN clearly provides evidence that MCU channels at the PM have a similar putative pore-forming structure that produces Ca^2+^ permeability. Further studies, such as knocking out EMRE and/or MICU subunits, are required to precisely understand their roles in MCU-S channels at PM.

In addition to *MCU-S*, we found another short variant *MCU-SS* in mice and confirmed that MCU-SS expression can also produce a Ca^2+^-permeable pathway at the PM (Supplementary Fig. 8). However, unlike MCU-S, MCU-SS is ~150 amino acids shorter in the N-terminal domain than MCU-L (Supplementary Table 1), which may produce the functional difference between MCU-S channels and MCU-SS channels. Specifically, MCU-SS is missing the majority of the N-terminal domain (amino acids 75-165 in MCU-L, see Supplementary Fig. 1), which contains the important structures for mtCUC dimerization, EMRE/MICU subunit association with MCU, and mtCUC channel activation^30,36–39^. Although the N-terminal domain is responsible for mtCUC dimerization, this uniplex dimerization is not necessary for maintaining its basic channel function^36^, as such N-terminal deletion mutants can still form Ca^2+^ permeable channels^32,38,40^ (but, in some cases, their activity is smaller than that of full-length MCU-L). Moreover, N-terminal domain deletion potentially leads to reduced stability of the MCU tetramer, despite the helix α1 after the N-terminal domain and the coiled-coil domain 1 remain intact^30,40^. Therefore, based on these observations in MCU-L, the channel formed by MCU-SS is expected to be less stable and may exhibit smaller conductance compared to MCU-S. Assessment of endogenous MCU-SS channel function, using the platelets from MCU knockout mice^41^, may be necessary to precisely compare its channel properties to those in MCU-S.

Our MCU-S overexpression studies show that MCU-L/MCU-S expression ratios may regulate the subcellular localization of MCU hetero-oligomers (Fig. 3A-E and Supplementary Fig. 11), suggesting that the majority of human cell types/tissues which we tested may not possess endogenous MCU channels outside of mitochondria, except for platelets (Fig. 1). Indeed, we detected a Ru360-sensitive, non-SOCE Ca^2+^-permeable pathway at the PM of human platelets, which can produce SOCE-independent platelet aggregation under thrombin stimulation (Fig. 4). Human platelets have three major molecular mechanisms that elevate [Ca^2+^]_c_ during their activation^27,42^: 1) ER-Ca^2+^ release; 2) Ca^2+^ entry via PM channels, including SOCE, transient receptor potential channel family, and receptor-operated Ca^2+^ channels; and 3) mPTP opening triggered by the mtCa^2+^ uptake via mtCUC. Supramaximal [Ca^2+^]_c_ signal is required for transformation into procoagulant platelets during in vitro experiments^28,41^. Although the PM-mediated Ca^2+^ entry mechanism holds the smallest impact on [Ca^2+^]_c_, the assessment of recent studies using transgenic mouse models with knockout of major SOCE components revealed that the PM-Ca^2+^ entry mechanism via SOCE is also essential for procoagulant platelet formation^27,42,43^. Lastly, we also showed that Ca^2+^ permeability via MCU-S channels at the PM is capable of modulating thrombin-induced actin cytoskeleton remodeling and cell morphological changes (Fig. 4), which precede platelet aggregation or adhesion. Since MCU-S-mediated cytoskeleton remodeling still exists under the inhibition of mtCa^2+^ uptake (Supplementary Fig. 15), our observation also suggests that the MCU channels expressed outside of the mitochondria are involved in platelet activation.

In conclusion, we identified a human MCU variant MCU-S that lack the MTS, which enables the MCU channels to localize outside of mitochondria. This is completely distinct from the originally reported MCU function as a highly mitochondria-specific Ca^2+^ channel. Expression ratios of the conventional mitochondria-targeted MCU and the non-mitochondria-targeted variants regulate the subcellular localization of the MCU channels. MCU-S forms Ca^2+^-permeable channels at the PM of platelets and participates in the Ca^2+^-signaling for platelet activation. Our findings provide additional insights into the molecular basis of MCU variant-dependent cellular Ca^2+^ handling in physiological and potentially in pathological conditions in which both cytosolic and mitochondrial Ca^2+^ dynamics play key roles. Key limitations of this study are the lack of 1) MCU variant-specific antibodies due to the high similarity between the matured MCU-L and MCU-S proteins, and 2) a MCU variant-specific knockout system, which significantly hinders our precise dissection of the endogenous MCU-S functions from MCU-L. Future studies will address the each variant’s function of each variant in vivo by establishing transgenic mice with specific expression of human MCU-S or MCU-L in a MCU-knockout background^41^. Since the use of alternative promoters is associated with various diseases, including cancer^44^, exploring the changes in *MCU-L*/*MCU-S* ratio in various human diseases may offer mechanistic insights into the regulatory complexities of MCU functions in both human physiology and pathophysiology. Since some members of the bacteria, yeast, and ciliate groups contain MCU homologs without MTS^45^, characterization of MCU variants may also help to elucidate the role of mtCa^2+^ transport in the evolution of mitochondria as well as the emergence of eukaryotic life.

## Methods

### Ethical approval

All animal experiments were performed in accordance with the Guidelines on Animal Experimentation of Thomas Jefferson University (01241, and 01387), Rhode Island Hospital (501917, and 869645), and University of Minnesota (1806-36049A, and 1810-36441A). The study protocols were approved by the Institutional Animal Care and Use Committee at each institution. The investigation conformed to the Guidelines for the Care and Use of Laboratory Animals published by the US National Institutes of Health (NIH).

### Animals

Adult FVB and C56BL/6 mice, and neonatal Sprague-Dawley rats were obtained from Taconic Bioscience (Rensselaer, NY, USA), Jackson Laboratory (Bar Harbor, ME, USA), and Envigo (Indianapolis, IN, USA), respectively. All the animals were used for the source for obtaining the primary cells. Laboratory rats and mice maintained under social housing in solid-bottom cages with proper bedding and kept under environmental enrichment in controlled temperatures (22-24°C) and 12-hr light cycles.

### Antibodies, plasmids, chemicals and reagents

The antibodies and plasmids used for the experiments are listed in Supplementary Tables 4 and 5, respectively. All chemicals and reagents were purchased from Sigma-Aldrich Corporation (St Louis, MO, USA) otherwise listed in Supplementary Table 9.

### Variants information acquisition and sequence analysis

Variants information of human *MCU and* mouse *Mcu* genes were sourced from the Ensembl database^46^. Conserved domains of variants were analyzed by the Clustal Omega multiple sequence alignment program of the European Bioinformatics Institute^47^. The mitochondrial targeting probabilities of variants were determined using the MitoProt II prediction program^48^.

### Cells, culture, and transfection

All the cell lines used in this study were listed in Supplementary Table 10.

HEK293T cells were maintained in Dulbecco’s modified Eagle’s medium (DMEM) supplemented with 4.5 g/L glucose, 1 mM sodium pyruvate, 1% L-glutamine, 10% fetal bovine serum (FBS), 100 U/ml penicillin, and 100 μg/ml streptomycin at 37 °C with 5% CO_2_ in a humidified incubator.

C2C12 mouse myoblast cells were maintained in DMEM supplemented with 1.0 g/L glucose, 1 mM sodium pyruvate and 1% L-glutamine, 20% FBS, 100 U/ml penicillin, 100 μg/ml streptomycin and 0.25 μg/mL fungizone and at 37 °C with 5% CO_2_ in a humidified incubator.

MEG-01 cells were maintained in RPMI 1640 medium supplemented with 10% FBS, 100 U/ml penicillin, 100 μg/ml streptomycin at 37 °C with 5% CO_2_ in a humidified incubator. Cell counting and cell size measurements before and after the adhesion on the glass bottom plates were performed by EVE™ Automated Cell Counter, NanoEnTek (Seoul, Republic of Korea).

HEK293T cells stably overexpressing MCU variants were generated transfecting with plasmids carrying MCU variants^16,49^. Cells stably overexpressing pcDNA3.1(+) or pEGFP-N1 were used as controls. HEK293T and C2C12 cells stably knocking down MCU were generated by transfecting with plasmids carrying shRNA targeted to the 3’-UTR of human *MCU* and mouse *Mcu*, respectively. Cells stably overexpressing empty PLKO.1 plasmid was used as a control. Stable cell lines were maintained in 1200 μg/ml G-418 or 0.5 μg/ml puromycin.

NCMs were enzymatically isolated from ventricles from two- to three-day-old Sprague Dawley rats using collagenase type 2^50^. Rats were euthanized by decapitation and hearts were harvested for NCM isolation. NCMs were plated onto gelatin-coated dishes and maintained in DMEM supplemented with 4.5 g/L glucose, 1 mM sodium pyruvate, 1% L-glutamine, 10% FBS, 100 U/ml penicillin, and 10 mg/ml streptomycin at 37 °C with 5% CO_2_ in a humidified incubator.

Adult cardiomyocytes (ACMs) and cardiac fibroblasts (ACFs) were isolated from ventricles of 8-month-old adult FVB hearts by retrograde heart perfusion with collagenase type 2^49^. ACMs were allowed to settle by gravity in the washing buffer with 2% BSA and a stepwise increase in Ca^2+^ concentration ([Ca^2+^]) from 20 μM to 1.0 mM and then used fresh. ACFs were cultured in DMEM/F12 with 10% FBS, 100 U/ml penicillin, and 100 mg/ml streptomycin, and collected after 48 h.

Human platelets from healthy donors were commercially obtained and the donor information provided by the companies was listed in Supplementary Table 6. Platelets in human platelet-rich plasma (PRP) were collected by centrifugation at 800 × g for 20 min at room temperature. Platelets were stored in Tyrode’s buffer with 5 mM glucose and BSA. All platelets from donors were used for experiments within 30 h from collection.

Mouse platelets are collected from adult FVB mice^51^. Cardiac punctures were performed immediately after euthanasia by CO_2_. The blood was anti-coagulated by rapid mixing with the citrate-dextrose solution (ACD buffer) during collection. The blood from three mice was pooled, and platelet-rich plasma was collected. After the addition of prostaglandin E1 (0.25 µM) to prevent platelet activation, platelets were collected by centrifugation at 1250 × *g* at room temperature for 10 min.

For transient transfections, HEK293T, C2C12, MEG-01, and NCMs were transfected with plasmids using FUGENE-HD transfection reagent and used for experiments 48–72 h after transfection^16^.

### RNA extraction, reverse transcription, and quantitative reverse transcription PCR (RT-qPCR)

Total RNA was isolated from mouse cells/tissues and human cell lines using the Qiagen RNeasy Mini Kit. For human platelets and MEG-01 cells, total RNA was isolated using TRIzo. Total RNA samples from the transfected MEG-01 cells were treated with DNase or the removal of the plasmid DNAs and processed with Zymo RNA Clean & Concentrator-5 kit. RNA quantity and purity were measured using a NanoDrop 2000 spectrophotometer (NanoDrop Technologies, Wilmington, DE, USA). cDNA was synthesized from 1.0 or 2.0 µg of total RNA using the iScript reverse transcription kit from Bio-Rad Laboratories (Hercules, CA, USA) or Superscrip IV VILO Master Mix from Thermo Fisher Scientific (TFS) (Waltham, MA, USA), respectively. Total cDNAs from various human tissues were commercially obtained and the donor information of human total cDNAs provided by the companies was listed in Supplementary Table 11.

The primer set for the amplification of *MCU and Mcu* cDNA was designed according to Ensembl sequences using the Primer3 program^39^ (Supplementary Table 2). All other primer sets for the assessment of human and mouse cells/tissues were listed in Supplementary Table 3. All primers were purchased from Integrated DNA Technologies, Inc. (Coralville, IA, USA) and Sigma-Aldrich. The PCR products of *MCU/Mcu* variants from HEK293T cells were separated by electrophoresis on 2.0% agarose gels in Tris-borate-EDTA (TBE) buffer, stained with ethidium bromide, and visualized under ultraviolet light.

For DNA sequence analysis, amplified PCR products were extracted from agarose gel using the QIAquick gel extraction kit and were sequenced by the dideoxy (Sanger) approach at Integrated DNA Technologies, Inc. Sequences were further compared with the full-length human MCU mRNA sequence (Supplementary Fig. 3).

RT-qPCR analysis for mouse cells/tissues, and human cell lines in Fig. 1F and Supplementary Fig. 4 was performed using an iCycler thermocycler (Bio-Rad Laboratories) with a TaqMan RT-PCR Master Mix (Bio-Rad Laboratories) or Fast SYBR Green Master Mix with an Applied Biosystems 7500 Fast RT-PCR System (TFS).

RT-qPCR for human platelets and tissues in Fig. 1H was performed using an Applied Biosystems 7500 Fast RT-PCR System (TFS) with an Applied Biosystems PowerUp SYBR Green Master Mix. RT-qPCR for MEG-01 cells in Fig. 4J and Supplementary Fig 14 was performed using a QuantStudio™ 3 (TFS) with an Applied Biosystems PowerUp SYBR Green Master Mix. The relative transcript levels for human and mouse MCU variants were determined by the ΔΔC_T_ method with GAPDH as the endogenous reference gene.

### Protein fractionations, Western blot analysis, and immunoprecipitation

Whole cell lysates were prepared using a commercial lysis buffer with 1 mM PMSF and 1% protease inhibitor cocktail. Mitochondria-enriched protein fractions and cytosolic protein fractions containing ER proteins were isolated as previously described with different centrifugation^16,50^.

Immunoprecipitation was performed as we previously described^16,50^. Briefly, protein lysates (500 μg) were incubated with primary antibodies (1 μg) overnight at 4 °C, followed by the addition of Protein A/G Plus Agarose. Immunocomplexes were washed with lysis buffer and subsequently subjected to Western blot.

For biochemically measuring F-actin/G-actin ratio, Adhered MEG-01 on the glass bottom dishes were detached, collected, washed with PBS, and lysed with actin stabilization buffer containing 100 mM PIPES in pH 6.9, 30% glycerol, 5% DMSO, 1 mM MgSO_4_, 1 mM EGTA, 1% Triton X-100, 1 mM ATP and 1% protease inhibitor cocktail^52,53^. Lysates were incubated on ice for 10 min and centrifuged at 100,000 × g for 60 min at 4 °C. Supernatant was corrected as a fraction containing G-actin. The pellets containing F-actin were solubilized with actin depolymerization buffer containing 100 mM PIPES in pH 6.9, 1 mM MgSO_4_, 10 mM CaCl_2_, and 5 μM cytochalasin D and incubated on ice for 30 min^52,53^.

All the protein samples were separated by sodium dodecyl sulfate polyacrylamide (SDS)-PAGE and were transferred to the nitrocellulose membranes and incubated with primary antibodies, followed by treatment with fluorescence-conjugated secondary antibodies. Immunoreactive bands were visualized by Odyssey Infrared Imaging System (LI-COR Biotechnology, Lincoln, NE, USA) and quantified by densitometry using Image J software (NIH) and Image Studio Lite Version 5.2 (LI-COR Biotechnology)^16,50^.

### Cell surface protein biotinylation assay

To distinguish the cell surface localization of MCU, a cell-surface biotinylation assay was performed using Pierce Cell Surface Protein Isolation Kit and Pierce Cell Surface Biotinylation and Isolation Kit^18^.

HEK293T cells stably overexpressing GFP-tagged proteins plated in 15-cm dishes were washed with an ice-cold Ca^2+^ and Mg^2+^-free phosphate-buffered saline (PBS) and labeled with EZ-Link Sulfo-NHS-SS-Biotin for 30 min at 4 °C, followed by quenching and harvesting with tris-buffered saline (TBS). For collecting surface protein from human platelets, platelets were rinsed once with PBS at room temperature and incubated with EZ-Link Sulfo-NHS-SS-Biotin in PBS for 30 min at room temperature. The biotin labeling reaction was quenched with cold PBS containing 100 mM glycine for 10 min.

After labeling, cells and platelets were centrifuged at 500 × *g* for 3 min at 4 °C, and the pellets were treated with lysis buffer containing protease inhibitor cocktail, followed by a brief sonication, and incubated on ice for 30 min with vortexing every 5 min for 5 s. Next, cell lysates were centrifuged at 10,000 × *g* for 2 min at 4 °C, and supernatants were collected in separate tubes. To isolate labeled proteins, supernatants were incubated with NeutrAvidin agarose for 60 min at room temperature. Biotinylated protein-NeutrAvidin agarose complex was collected, washed with the washing buffer containing protease inhibitor cocktail, and eluted with the Pierce Lane Marker Non-Reducing Sample Buffer with 10 mM dithiothreitol for 60 min at room temperature, followed by centrifugation at 1000 × *g* for 2 min. Eluted protein samples were mixed with sample buffer and subjected to SDS-PAGE and Western blot.

### Live cell Ca^2+^ imaging

HEK293T cells and NCMs were plated on glass bottom dishes (MatTek, Ashland, MA, Cellvis, Mountain View, CA, and Matsunami Glass, Osaka, Japan), loaded with membrane-permeant acetoxymethyl (AM) ester forms of Fluo-3-AM and/or Fura-Red-AM at 37 °C, and the changes in cytosolic Ca^2+^ concentration ([Ca^2+^]_c_) were measured at room temperature by FV1000 or FV3000 laser scanning confocal microscope (Olympus, Tokyo, Japan) in modified HEPES Tyrode’s solution (in mM): NaCl, 136.9; KCl, 5.4; CaCl_2_, 1; MgCl_2_, 0.5; NaH_2_PO_4_, 0.33; HEPES, 5; glucose, 5; pH 7.40 adjusted with NaOH^15,16,49^.

For measuring the changes in Ca^2+^ concentration at mitochondrial matrix ([Ca^2+^]_mt_), cells were transiently transfected with mitochondrial matrix-targeted Ca^2+^ biosensor mtRChamp1h, and the mtRChamp1h fluorescence was monitored with excitation wavelength at 543 nm and emission wavelength at 560-660 nm at room temperature^54^.

To assess the PM localization of GFP-tagged proteins, cells were stained with a PM potential-sensitive dye Di-8-ANEPPS^17^. Di-8-ANEPPS was excited with a 445 nm laser and emission was collected at 587-637 nm, while GFP was excited with a 488 nm laser and emission was collected at 500–541 nm.

To detect the MCU variant associations in live HEK293T cells, a Förster resonance energy transfer (FRET)-based method was used^22^. GFP and monomeric cherry (mCherry) were set up as an energy donor and an energy acceptor, respectively. Smac-mCherry^55^, an intermembrane space protein, was used as a negative control. GFP was excited with a 488 nm laser and emission was collected at 500–530 nm. mCherry was excited with a 561 nm laser and emission was collected at 575–675 nm. FRET signal was collected at 575–675 nm.

To assess and compare the *Ψ*_*m*_ between the different cell lines, Tetramethylrhodamine, ethyl ester (TMRE)/ MitoTracker Deep Red FM (MtDRFM) ratio was measured^56^. TMRE is a *Ψ*_*m*_-sensitive dye, and MtDRFM can label both active and inactive mitochondria, which does not depend on *Ψ*_*m*_.

For comparing the basal cytosolic Ca^2+^ concentration, cells loaded with Ca^2+^-sensitive dyes Fluo-3 and Fura-Red and monitored using a Nikon TE2000 epifluorescence microscope equipped with 60× oil TIRF objective lens (Nikon, Tokyo Japan) and a Retiga EXi camera (QImaging, Surrey, BC, Canada) at room temperature. Fluo-3/Fura-Red ration was measured in each microscopic view.

Human platelets were resuspended in modified platelet HEPES Tyrode’s buffer (in mM: 136.5 mM NaCl, 136.5; KCl, 2.68; NaHCO_3_, 11.9, NaH_2_PO_4_, 0.42; MgCl_2_, 0.5; HEPES, 5; pH 7.40 adjusted with NaOH) containing 0.35% (w/v) human serum albumin (HSA) 0.35% (w/v), and prostaglandin I2 (PGI_2_, 0.5 μM). Platelets were immobilized on the glass-bottom dishes coated with Cell-Tak, loaded with Fluo-3-AM for 30 min at room temperature, and washed with modified platelet HEPES Tyrode’s buffer containing 0.35% HSA and 0.5 μM PGI_2_. Ca^2+^ imaging was performed in modified platelet HEPES Tyrode’s buffer containing 0.1% HSA and apyrase (0.02 U/mL)^23^ using an FV3000 laser scanning confocal microscope (Olympus) at room temperature. All the live cell imaging data were analyzed using Fiji ^57^.

### Quantitative co-localization and morphology analysis in live cells

Colocalization analysis of the live cell images and the generation of color and frequency scatter plots were performed using ImageJ software (NIH) with an Intensity Correlation Analysis plugin (The Bob and Joan Wright Cell Imaging Facility, Toronto Western Hospital, Toronto, Ontario, Canada). Colocalization was assessed using Pearson’s correlation coefficient^15,50^. The values for Pearson’s correlation range from 1 to −1. A value of 1 represents perfect correlation, −1 represents perfect exclusion, and 0 represents random localization.

For the morphology assay of MEG-01 cells, non-adherent MEG-01 cells were resuspended in modified platelet HEPES Tyrode’s buffer with 0.4 mM CaCl_2_ and plated on glass-bottom dishes. Adherent cells were loaded with a cell-permeant fluorescent probe, Calcein Violet-AM, for 20 min, followed by the stimulation with thrombin (0.1 U/mL) for 30 min^26^. Cell morphology was measured under an FV3000 laser scanning confocal microscope (Olympus) at room temperature. Cells stained with Calcein Violet were defined as viable cells, and their circularity was assessed from phase contrast images using Fiji^57^.

### Immunocytochemistry

Immunocytochemistry for HEK293T cells and human platelets was performed as we previously described^50^. Briefly, HEK293T cells plated on glass bottom dishes and platelets immobilized on poly-L-lysine-coated on glass bottom dishes were fixed with 4.0% paraformaldehyde at 4 °C for 10 min, permeabilized with saponin at room temperature for 10 min, blocked with 10% goat serum, and incubated with primary antibodies overnight, followed by incubation with fluorescent secondary antibodies for 1 hr. Immuno-stained images were acquired with an FV3000 laser scanning confocal microscope (Olympus) at room temperature. Negative control experiments were performed using secondary antibodies without incubation of primary antibodies, which showed no noticeable labelling.

For the assessment of protein aggregation, the PROTEOSTAT® Aggresome detection kit was used^58^. Briefly, HEK293T cells were fixed with 4.0% paraformaldehyde at room temperature for 30 min, permeabilized with Triton X-100 at 4 °C for 30 min, and stained with an aggresome dye PROTEOSTAT^®^ and Hoechst 33342 at room temperature for 30 min. The cells treated with 5 µM MG-132 were used as positive control. PROTEOSTAT^®^ dye was excited with 514 nm laser, and emission was collected at 570–620, while GFP was excited with 488 nm laser, and emission was collected at 490–510 nm. No significant bleed-through of PROTEOSTAT® dye fluorescence into GFP detection was confirmed by the negative controls without GFP or without PROTEOSTAT® dye.

Immunostaining of F- and G-actin in MEG-01 cells was performed following the published protocol^59^. Briefly, cells were first fixed with 4.0% paraformaldehyde for 15 min on ice, and washed with cold PBS, followed by additional fixation with cold acetone at -20 °C for 5 min. For detecting G-actin, cells were washed again with cold PBS after fixation, blocked with 10% goat serum, and incubated with a monoclonal anti-actin JLA20 antibody for overnight, followed by incubation with Alexa488-conjugated secondary antibody for 1 h. The JLA20 antibody developed by Dr. J.J.-C Lin^60^ was obtained from the Developmental Studies Hybridoma Bank, created by the NICHD of the NIH and maintained at The University of Iowa, Department of Biology, Iowa City, IA 52242. For staining F-actin, cells were incubated with CF568-conjugated phalloidin and DAPI before observation.

### Electrophysiology

Whole-cell patch-clamp MCU Ca^2+^ current (*I*_*Ca, MCU*_) was carried out for measuring the Ca^2+^ current via hMCU-S (*I*_*Ca, MCU*_) from HEK293T cells stably overexpressing hMCU-S-GFP at room temperature using an Axopatch 200 amplifier with Digidata 1550B, CV-203BU head stage, and PClamp 10 software (Molecular Devices, San Jose, CA, USA). Briefly, before recordings, culture medium was removed, and cells were washed with Tyrode’s solution containing (in mM): 135, NaCl; 5, KCl; 1, MgCl_2_; 2, CaCl_2_; 10, HEPES; 5.6 Glucose; pH was adjusted to 7.4 with NaOH. For eliminating endogenous K^+^ currents, Tyrode’s solution was modified by substituting KCl with CsCl and used as a bath solution (in mM): 135, NaCl; 5, CsCl; 1, MgCl_2_; 2, CaCl_2_; 10, HEPES; 5.6, Glucose; pH was adjusted to 7.4 with CsOH. The composition of the pipette solution was set up as nominally free Ca^2+^ as follows (in mM): 135, CsCl; 5, NaCl; 1, MgCl_2_; 10, HEPES; pH was adjusted to 7.2 with CsOH. The *I*_*Ca, MCU*_ were elicited with a standard voltage ramp protocol^19^. From a holding potential of 0 mV, a 100-ms step hyperpolarization to -160 mV was followed by an ascending voltage ramp (from −160 mV to +80 mV at 120 mV/s), applied every 5 seconds. MCU blocker Ru360 (1 μM) was added, and the Ru360-sensitive component was defined as *I*_*Ca, MCU*._ Recording electrodes were pulled from thin-wall borosilicate glass capillaries with filament (World Precision Instruments, Sarasota, FL, USA) using a pipette puller (model P-97, Sutter Instruments, Novato, CA, USA). Data were low-pass filtered at 5 kHz and digitized at a sampling rate of 20 kHz.

### Microtiter plate-based light transmission aggregometry

Aggregometry was conducted in 96-well plates with a standard plate reader^61,62^. Human platelet count was adjusted to a standard OD using a plate reader at 405 nm^61^. An MCU blocker 3 µM Ru360, or its vehicle (water) was added to the wells containing the platelets, followed by the addition of thrombin. After thrombin addition, the plate was shaken for 5 min at 37 °C, and the absorbance was read on an Accuris Microplate Reader at 405 nm (Benchmark Scientific, Sayreville, NJ, USA). Wells with platelets with Tyrode’s solution were used as unstimulated resting platelet suspensions representing 0% aggregation. Wells with only Tyrode’s solution were used as buffer-only controls representing 100% aggregation. We also confirmed that there was around 5% or less spontaneous, agonist-independent aggregation in our system.

### Statistics and reproducibility

Statistical analyses were conducted using Origin Software (OriginLab Corporation, Northampton, MA, USA) and Excel v16.0 (Microsoft, Redmond, WA, USA). Experiments were performed independently at least two times with similar results to ensure reproducibility. Results are shown as bar graphs with all the points and error bars (representing standard error: SEM) or box-and-whisker plots. Box & whiskers plots show the minimum and maximum values, 1st and 3rd quartiles, and the median. Mean average in each group was represented by “x” in the box. An unpaired Student’s t-test was performed for two data sets. For multiple comparisons, one-way ANOVA followed by the post hoc Tukey test was performed. The value of *p*  <  0.05 was considered as significant. Number of independent replicates (*n*) for each experimental group is shown in the figure legends or in the figure panels using parentheses.

### Reporting summary

Further information on research design is available in the Nature Portfolio Reporting Summary linked to this article.

## Supplementary information

Supplementary Information

Description of Additional Supplementary File

Supplementary Data 1

Reporting Summary

Transparent Peer Review file

## Data Availability

All source data underlying the graphs presented in the main and Supplementary Figs. are uploaded as Supplementary Data 1. All the uncropped and unedited blot/gel images are shown in Supplementary Figs. 16-19.
