# Supplementary material for: Mitochondrial Ca2+ uniporter (MCU) variants form plasma-membrane channels
Source: Commun Biol. 2026 May 20;9:1074. doi: 10.1038/s42003-026-10285-x (PMC13458766; doi:10.1038/s42003-026-10285-x)
Supplement: Supplementary file 1 [file 42003_2026_10285_MOESM1_ESM.pdf]

Supplementary information for

Mitochondrial Ca^{2+} uniporter (MCU) variants form plasma-membrane channels

Iuliia Polina, Jyotsna Mishra, Michael W Cypress, Maria Landherr, Nedyalka Valkov, Isabel Chaput, Bridget Nieto, Brian Rhee, Ameneh Ahrari, Nathan DeMichaelis, Kye-Im Jeon, Ulrike Mende, Peng Zhang, Bong Sook Jhun, Jin O-Uchi

This PDF file includes:

Supplementary Figures S1 to S19

Supplementary Tables S1 to S11

Supplementary References

Supplementary Figures

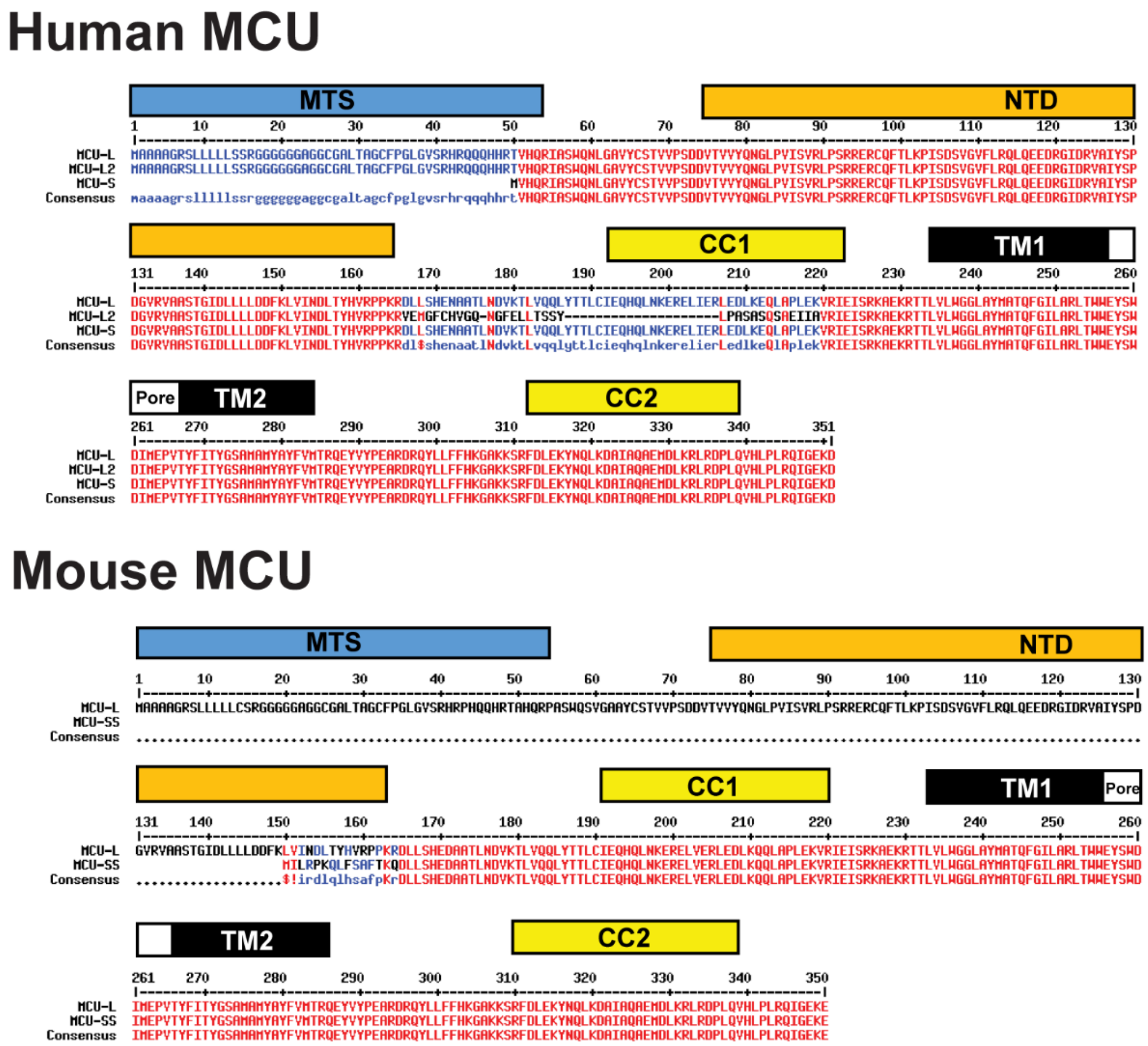

Supplementary Fig. 1. Alignment of the amino acid sequence of human and mouse MCU variant proteins.

Multiple sequence alignment was performed by MultAlin (http://multalin.toulouse.inra.fr/multalin/multalin.html). Mitochondria-targeted sequence (MTS) was predicted by MitoProt [1]. Key structure domains of MCU are shown based on the information from Uniprot (https://www.uniprot.org/uniprotkb/Q8NE86/entry). NTD, N-terminal domain; TM, transmembrane domain; and MTS, mitochondrial targeted sequence; CC, coiled coil domain.

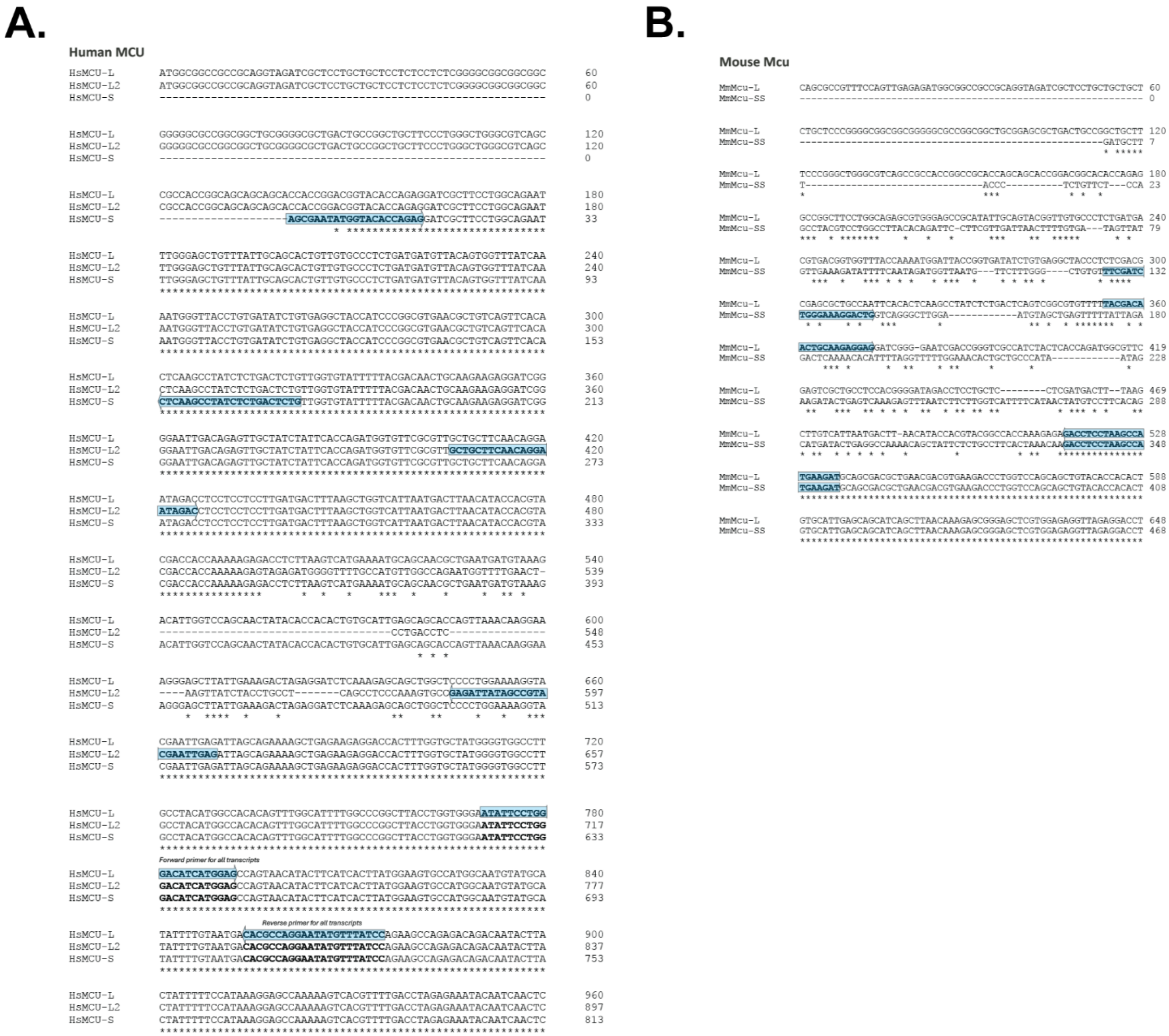

Supplementary Fig. 2. Design of specific PCR primers for detecting human *MCU* and mouse *Mcu* variants.

The binding sites of the primers specific to human *MCU* (**A**) and mouse *Mcu* variants (**B**) are shown in blue. Since it is difficult to design a human *MCU-L*-specific primer, the primer design shown "HsMCU-L" detects all human *MCU* variants.

Human MCU-L2

```
HsMCU-L2        CGCGTTGCTGCTTCAACAGGAATAGACCTCCTCCTCCTTGATGACTTTAAGCTGGTCATT   480
Seq_For         ------------------------------CCCTTTGGAATGACTTTTTAGCTGGTCATT   30
Seq_Rev-rc      -------CTGCTTCAACAGGAATAGACCTCCTCCTCCTTGATGACTTTAAGCTGGTCATT   53
                                              * * *          *** ***********

HsMCU-L2        AATGACTTAACATACCACGTACGACCACCAAAAAGAGTAGAGATGGGGTTTTGCCATGTT   540
Seq_For         AATGACTTAACATACCACGTACGACCACCAAAAAGAGTAGAGATGGGGTTTTGCCATGTT   90
Seq_Rev-rc      AATGACTTAACATACCACGTACGACCACCAAAAAGAGTAGAGATGGGGTTTTGCCATGTT   113
                ************************************************************

HsMCU-L2        GGCCAGAATGGTTTTGAACTCCTGACCTCAAGTTATCTACCTGCCTCAGCCTCCCAAAGT   600
Seq_For         GGCCAGAATGGTTTTGAACTCCTGACCTCAAGTTATCTACCTGCCTCAGCCTCCCAAAGT   150
Seq_Rev-rc      GGCCAGAATGGTTTTGAACTCCTGACCTCAAGTTATCTACCTGCCTCAGCTCCATGGCCC   173
                **************************************************  *

HsMCU-L2        GCCGAGATTATAGCCGTACGAATTGAGATTAGCAGAAAGCTGAGAAGAGGACCACTTTG   660
Seq_For         GCCGAGATTATAGCCGTACGAATTGAGGGT------------------------------   180
Seq_Rev-rc      ------------------------------------------------------------   173
```

Human MCU-S

```
HsMCU-S         ctttattcccctctgtgacttcctctgtgtgtgtgcatggggaaccggctccctcgagat      300
Seq_For         ------------------------------------------------------------      0
Seq_Rev-rc      -------------------------------------------------------CAGAT      5

HsMCU-S         ggatgctgcattgcttcaggaggcaagcttcatcctcttgggtccttaaaggtgcttttg      360
Seq_For         ------------------------------------------------------------      0
Seq_Rev-rc      AGGTTTCCCGACTGGAAAGC-GGGCAGTGAGCGCCACGCAAGTA-----ATGTGAGTTAA      59

HsMCU-S         ggggtcctttcgagggctactgtataagcgtgttcacgcgtgcctgaagcgggaagggtg      420
Seq_For         ------------------------------------------------------------      0
Seq_Rev-rc      TTTTTCATTAGGCACCCCA--------------GGCTTTACACTTGATGCTTCCGGTTTG      105

HsMCU-S         gtcagcaggcacaggagaagcgaatATGGTACACCAGAGGATCGCTTCCTGGCAGAATTT      480
Seq_For         ----------------------------------------GGGCCTATCCTGTCAGATTT      20
Seq_Rev-rc      TATGTTGTGTGGAATATTAGCGAATATGGTACACCAGAGGATCGCTTCCTGGCAGAATTT      165
                                                            **  *  *    ****

HsMCU-S         GGGAGCTGTTTATTGCAGCACTGTTGTGCCCTCTGATGATGTTACAGTGGTTTATCAAAA      540
Seq_For         GGGAGCTGTTTATTGCAGCACTGTTGTGCCCTCTGATGATGTTACAGTGGTTTATCAAAA      80
Seq_Rev-rc      GGGAGCTGTTTATTGCAGCACTGTTGTGCCCTCTGATGATGTTACAGTGGTTTATCAAAA      225
                ************************************************************

HsMCU-S         TGGGTTACCTGTGATATCTGTGAGGCTACCATCCCGGCGTGAACGCTGTCAGTTCACACT      600
Seq_For         TGGGTTACCTGTGATATCTGTGAGGCTACCATCCCGGCGTGAACGCTGTCAATTCACACT      140
Seq_Rev-rc      TGGGTTACCTGTGATATCTGTGAGGCTACCATCCCGGCTAACGCCGCTGC----------      275
                **************************************     *     *

HsMCU-S         CAAGCCTATCTCTGACTCTGTTGGTGTATTTTTACGACAACTGCAAGAAGAGGATCGGGG      660
Seq_For         CAAGACTATCTCTGAATCTGGCCCCCTCCTTCATAATGAGCACTAAAAAAATTAAATA--      198
Seq_Rev-rc      ------------------------------------------------------------      275
```

Supplementary Fig. 3. Sequence of PCR products purified from conventional gels.

PCR product was purified from the conventional DNA agarose gels (see Figure 1G) and sequenced using the dideoxy (Sanger) approach. Seq_For: sequencing read by forward primer, Seq_Rev-rc: sequencing read by reverse primer (reverse complement).

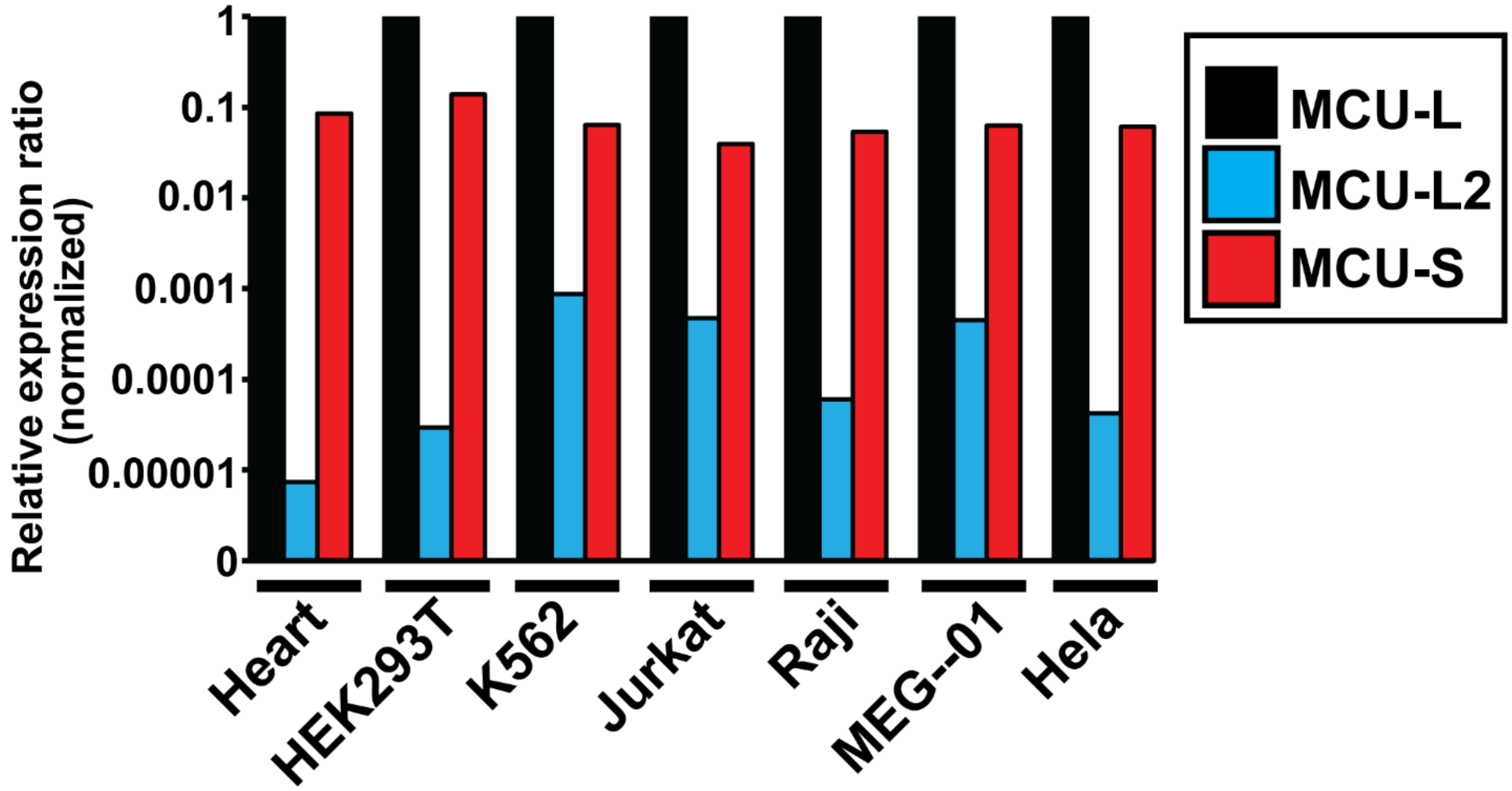

Supplementary Fig. 4. RT-qPCR analysis of *MCU* variants in human cell lines.

Human *MCU-L2* and *MCU-S* were measured using specific primers (see **Supplementary Figure 2** and **Supplementary Table 2**). The total *MCU* primer can detect all *MCU* variants, and quantitative results are shown. *MCU-L* mRNA amount was then estimated by subtraction of other variants from *MCU* total mRNA. Expression levels are normalized to the MCU-L value in each cell line. The Y axis is shown in a logarithmic scale.

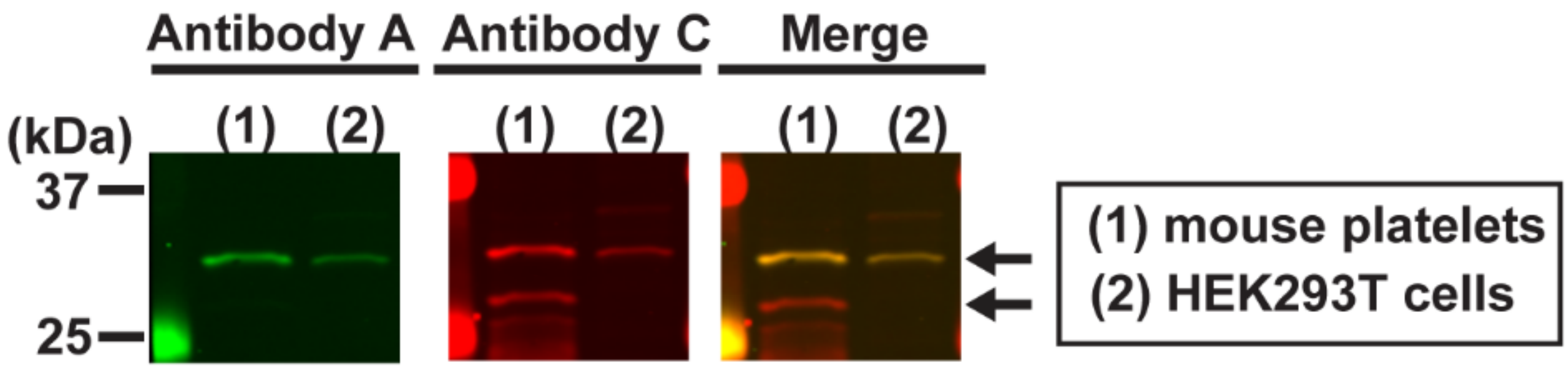

Supplementary Fig. 5. Detection of MCU variant proteins in mouse platelets.

MCU proteins in the whole cell lysates from mouse platelets were detected by an infrared imaging system (LI-COR Biotechnology). Double staining was performed using antibodies against the N-terminal region of MCU (Antibody A) and the internal region (Antibody C) (see also Supplementary Table 4). Lysates from HEK293T cells were used for comparison. The uncropped and unedited image are shown in Supplementary Fig.19.

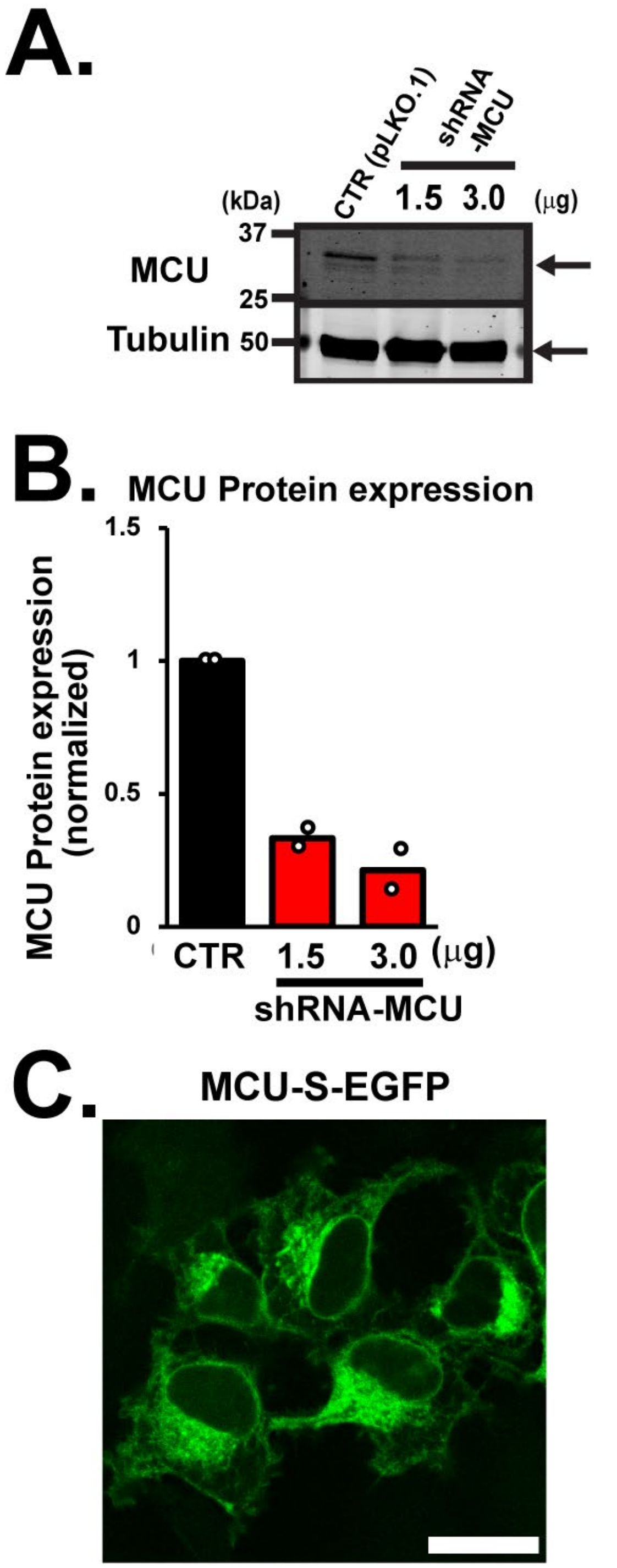

Supplementary Fig. 6. Generation of MCU-KD HEK293T cells.

A. Generation of MCU-KD cells using shRNA against the 3'-UTR of *MCU*. Control cells (CTR) were prepared by overexpression of an empty vector (pLKO.1). The uncropped and unedited image are shown in Supplementary Fig.19. **B.** Summary data of panel A. The bar plots indicates mean values. *n* = 2. **C.** Overexpression of MCU-S-GFP in HEK293Tcells stably overexpressing shRNA against the 3'-UTR of *MCU* (see also **Figure 2A**). Scale bar, 10 μm.

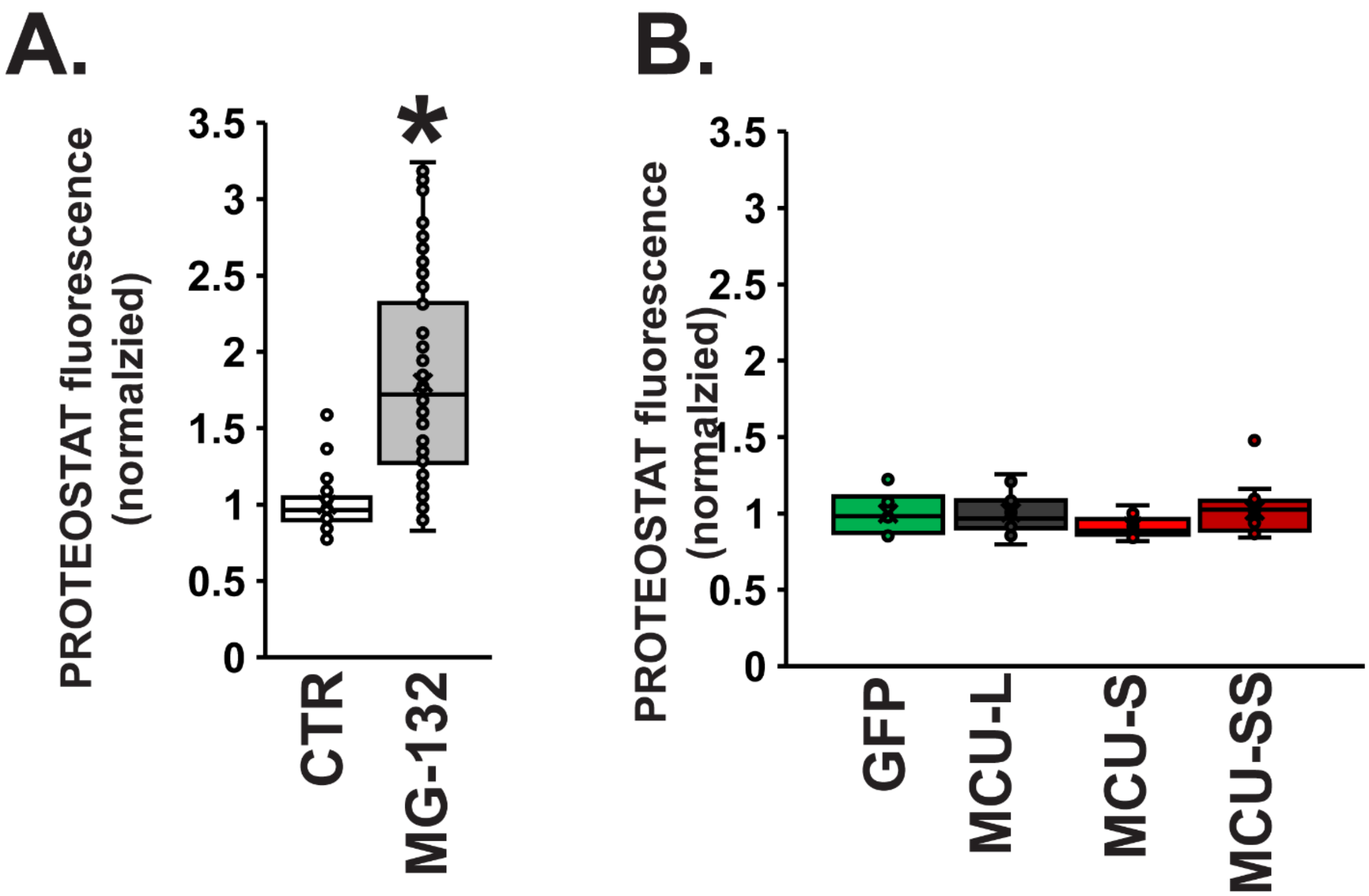

Supplementary Fig. 7. Overexpression of short MCU variants does not induce protein aggregation.

A. Protein aggregation measurements in fixed HEK293T cells. Cells were treated with a proteasome inhibitor, 5 μM MG-132, or DMSO as a vehicle (control: CTR) for 18 hrs. After treatment, cells were fixed and stained with PROTEOSTAT® dye. This dye specifically intercalates into the cross-beta spine of quaternary protein structures typically found in misfolded and aggregated proteins, which inhibits the dye's rotation and leads to strong fluorescence. The fluorescent intensity values from the MG-132 group were normalized to those in CTR cells. Box & whiskers plots show the minimum and maximum values, 1st and 3rd quartiles, and the median. Mean average in each group was represented by "x" in the box. *n* = 63 and 62; 1st quartile, 0.899 and 1.272; mean averages, 1.000 and 1.798; median values, 0.965 and 1.721; 3rd quartile, 1.047 and 2.320; maximum values, 1.267310676 and 3.241; and minimum values, 0.740146072 and 0.828178951 for CTR and MG123 groups, respectively. Unpaired T-test: *p<0.05. **B.** PROTEOSTAT® dye-staining in HEK293T cells stably overexpressing EGFP, MCU-L-GFP, MCU-S-GFP, and MCU-SS-GFP. Fluorescent values from MCU groups were normalized to those in GFP cells. *n* (left to right) = 10, 17, 19, and 16; 1st quartile (left to right), 0.872, 0.903, 0.861, and 0.886; mean averages (left to right), 1.000, 1.006, 0.916, and 1.019; median values (left to right), 0.983, 0.967, 0.886, and 1.024; 3rd quartile (left to right), 1.112, 1.086, 0.964, and, 1.084; maximum values (left to right), 1.274, 1.256, 1.054, and 1.161; and minimum values (left to right), 0.827234765, 0.797719912, 0.818818863, and 0.842492902.

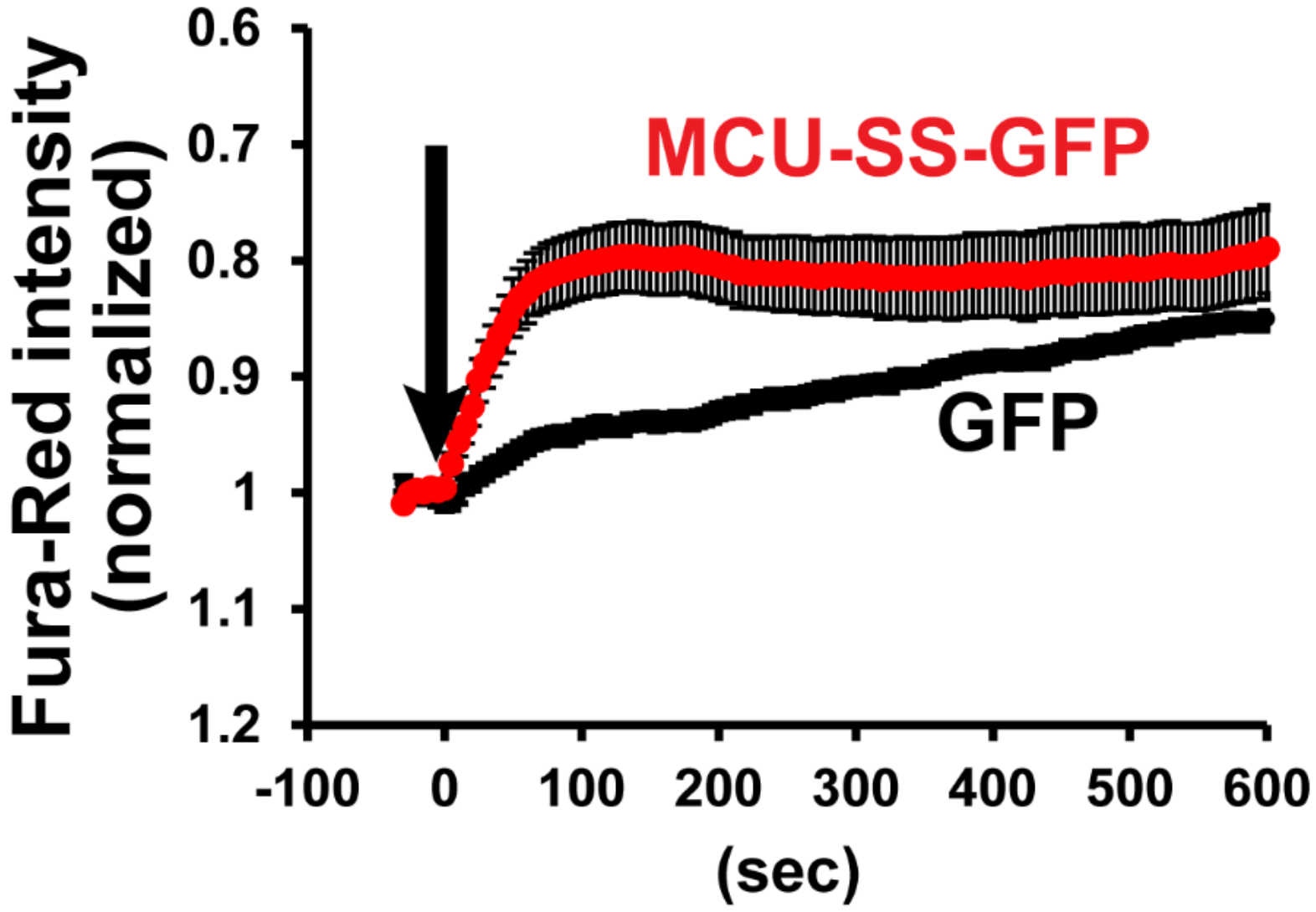

Supplementary Fig. 8. MCU-SS forms a Ca^{2+}-permeable pathway at the plasma membrane.

HEK293T cells stably overexpressing MCU-SS-GFP (*n*=28) or GFP (*n*=21) were stained with a Ca^{2+}-sensitive dye Fura-Red and changes in $[Ca^{2+}]_c$ were measured in response to re-admission of 2 mM Ca^{2+} (indicated by the arrow) following the 1-min removal of extracellular Ca^{2+} under the confocal microscopy. Upon an increase of extracellular Ca^{2+} concentration from 0 to 2 mM, cells stably overexpressing MCU-SS-GFP showed $[Ca^{2+}]_c$ elevation with faster kinetics compared to cells stably overexpressing GFP. Results are shown as mean value of each time points and error bars represent SEM.

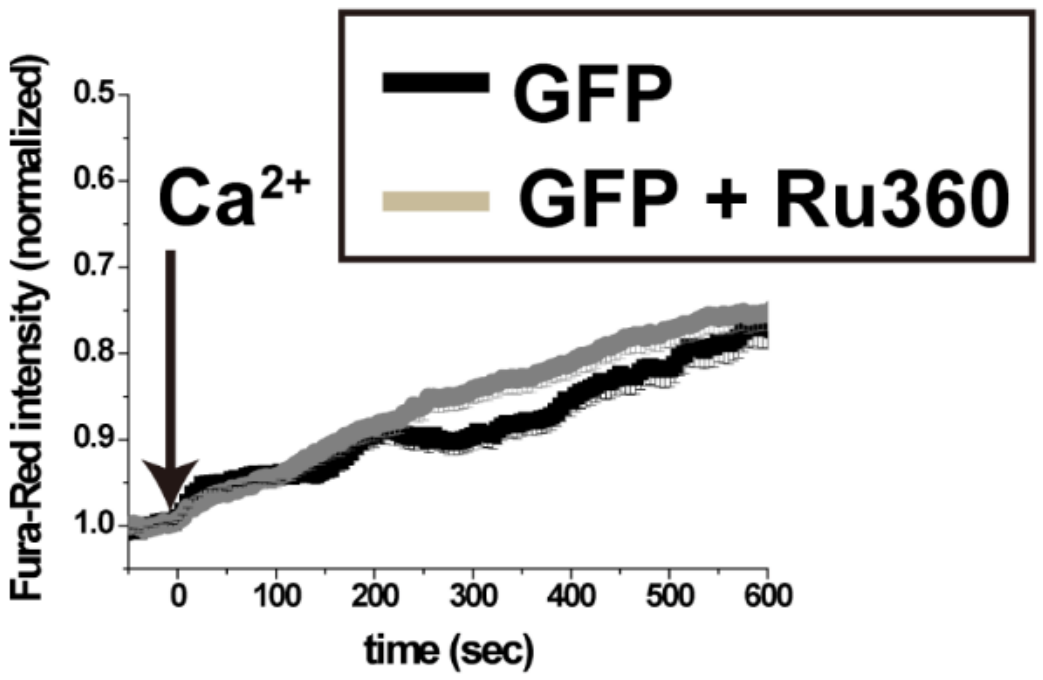

Supplementary Fig. 9. MCU blocker Ru360 does not show a significant effect on Ca^{2+} entry at the plasma membrane in control HEK293T cells.

Effect of 1 μM Ru360 on HEK293T cells overexpressing MCU-S-GFP. Changes in $[Ca^{2+}]_c$ in response to re-admission of the extracellular Ca^{2+} (2 mM, arrow) after the short-time removal of the extracellular Ca^{2+}, were assessed by Fura-Red. Fluorescent values of Fura-Red for each time point were normalized to the initial value before Ca^{2+} application. *n* = 24 for each group. Results are shown as mean value of each time points and error bars represent SEM.

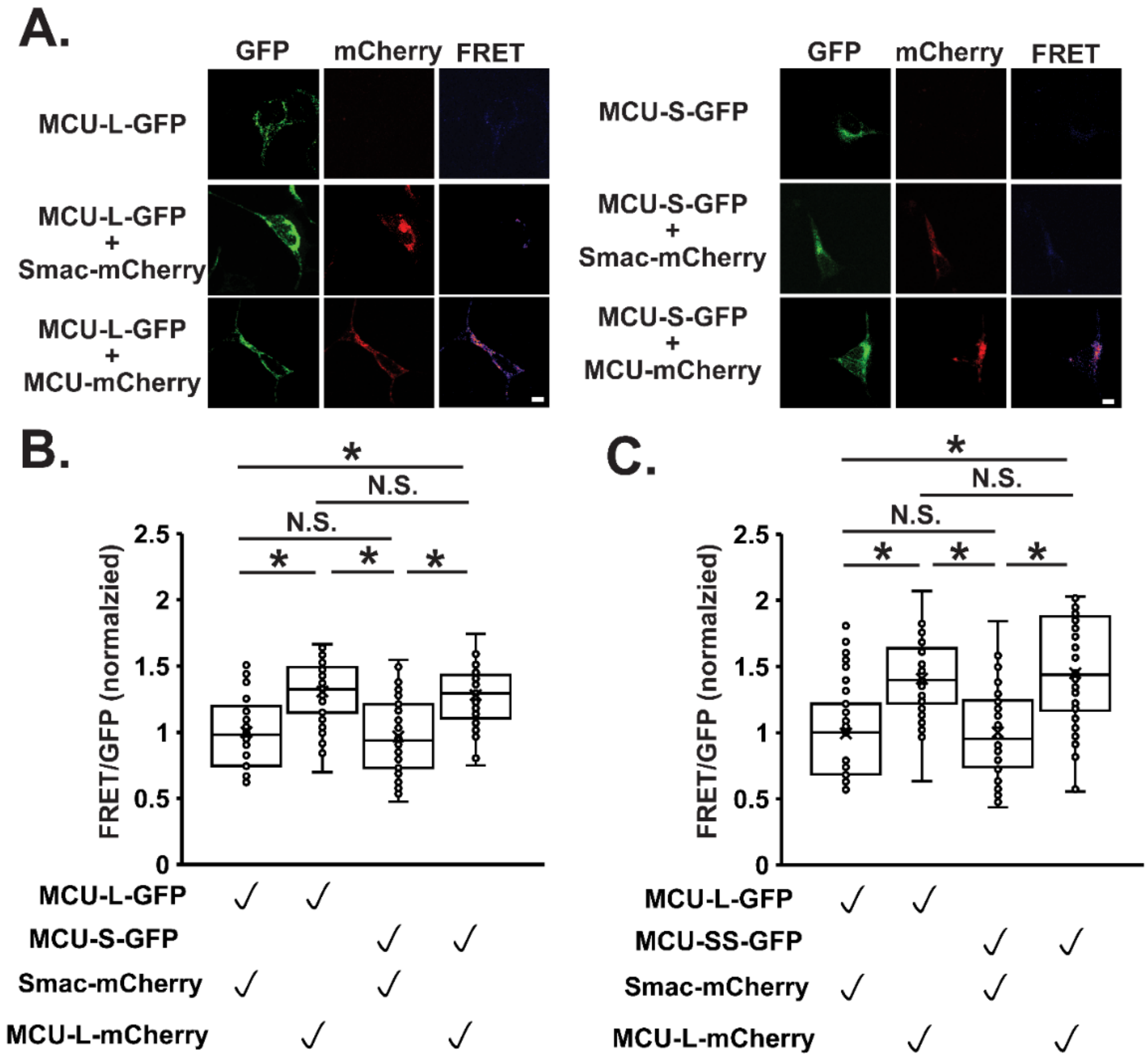

Supplementary Fig. 10. Short MCU variants interact with MCU-L.

A. Representative images in HEK293T cells transiently transfected with GFP-tagged MCU variants with either monomeric cherry (mCherry)-tagged MCU-L or Smac. Scale bars, 10 µm. Interaction between MCU-L and MCU-S in live HEK293T cells was assessed using the FRET method by setting up EGFP as an energy donor and mCherry fluorescent protein as an energy acceptor. Smac-mCherry, an intermembrane space protein, was used as a negative control. **B.** Summary data of panel A. The FRET value in each cell was compensated with the fluorescent value of GFP. FRET/GFP value was normalized with that in cells co-expressing MCU-L-GFP and Smac-mCherry. *n* = 38-76 for each group. Box & whiskers plots show the minimum and maximum values, 1st and 3rd quartiles, and the median. Mean average in each group was represented by "x" in the box. *n* (left to right) = 38, 68, 69, and 76; 1st quartile (left to right), 0.745, 1.151, 0.759, and 1.138; mean averages (left to right), 1.000, 1.327, 0.975, and 0.975; median values (left to right), 0.981, 1.345, 0.948, and 1.294; 3rd quartile (left to right), 1.197, 1.522, 1.204, and 1.427; maximum values (left to right), 1.645, 1.693, 1.547, and 1.743; and minimum values (left to right), 0.501764609, 0.697192105, 0.463670005, and 0.749665256. One-way ANOVA, Tukey's post-hoc test: *p<0.05. N.S., not significant. **C.** Interaction between MCU-L and MCU-SS in live HEK293T cells. *n* (left to right) = 55, 48, 46, and 50; 1st quartile (left to right), 0.328, 0.586, 0.355, and 0.559; mean averages (left to right), 0.477, 0.675, 0.481, and 0.695; median values (left to right), 0.480, 0.670, 0.457, and 0.689; 3rd quartile (left to right), 0.584, 0.785, 0.596, and 0.902; maximum values (left to right), 0.876, 0.993, 0.884, and 0.972; and minimum values (left to right), 0.269921775, 0.303605542, 0.209419095, and 0.266183961. One-way ANOVA, Tukey's post-hoc test: *p<0.05.

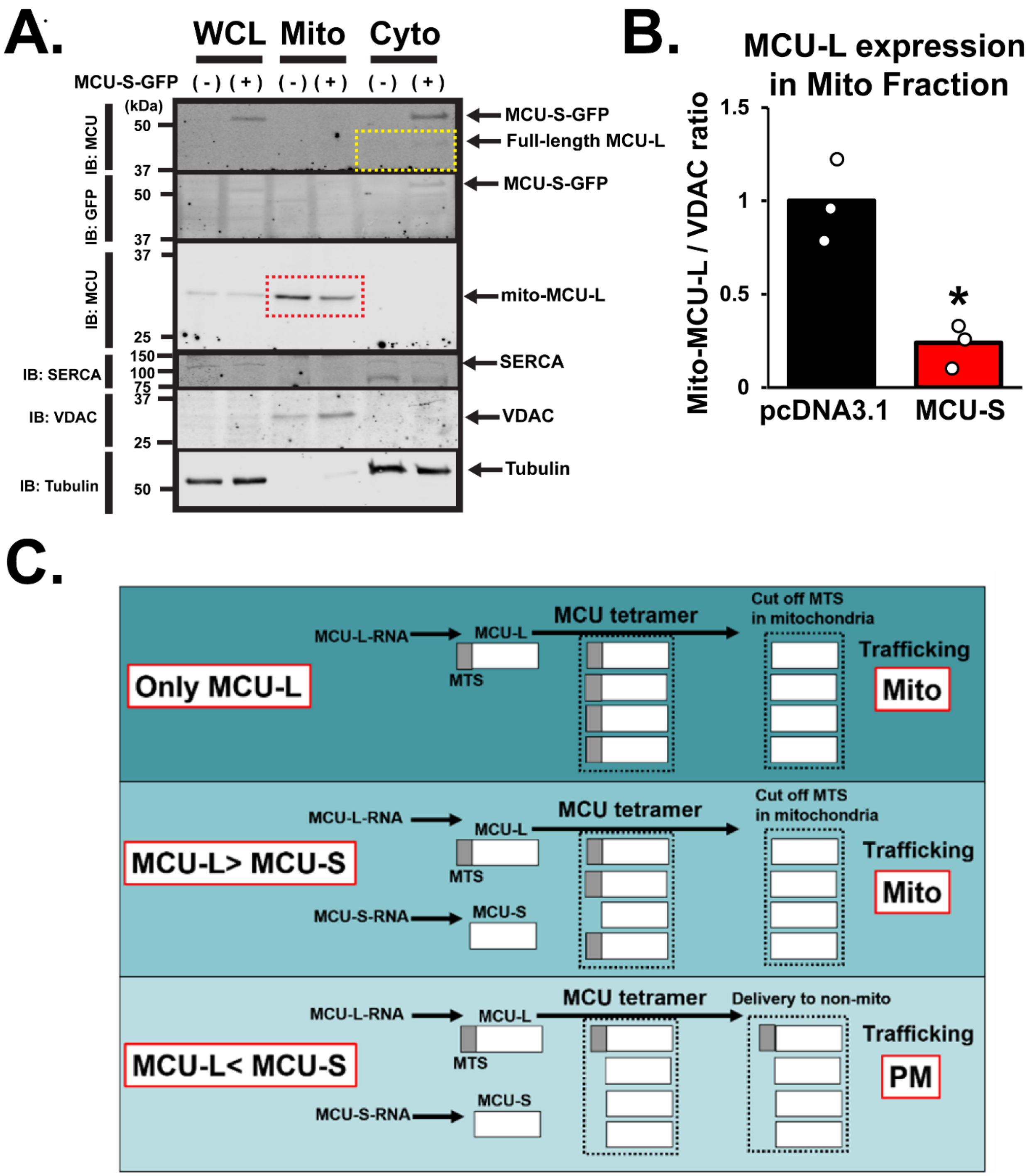

Supplementary Fig. 11. MCU-S inhibits mtCUC trafficking to mitochondria.

A. Detecting human MCU variants in HEK293T cells overexpressing MCU-S-GFP or GFP (as a control) using fractionated protein. The mitochondria-enriched protein fraction (Mito) and cytosolic protein fraction (Cyto, which contains the ER proteins and PM proteins) were isolated as previously described with different centrifugation (see details in Method section). Whole cell lysates (WCL) were also prepared for comparison. Tubulin was used as a loading control for WCL. SERCA and VDAC were used for loading and quality controls for Cyto and Mito fractions, respectively. The yellow-dotted box indicates the location of the band presumably corresponding

to endogenous full-length MCU-L protein that includes the MTS (i.e., ~40 kDa protein expressed in Cyto). The red-dotted box indicates the location of the bands presumably corresponding to endogenous MCU-L without MTS (i.e., ~32 kDa protein expressed in the Mito). The uncropped and unedited image are shown in Supplementary Fig.19. **B.** Summary data of panel A. The bar plots indicate mean values. *n* = 3 for each group. Unpaired T-test: **p*<0.05. **C.** Schematic diagram of the effect of MCU-S on MCU channel trafficking in the different MCU-L/MCU-S ratio. Oligomerization of ion-channel pore subunits mainly occurs in the ER and/or Golgi before delivery to the targeted organelle (Nagaya & Papazian, 1997) and the mtCUC pore is formed by MCU tetramer. If the MCU-L subunit number is larger than MCU-S within the MCU heteromeric tetramer (*i.e.*, in the majority of cells/tissues under normal conditions, See Figure 1), the majority of MCU proteins composing the tetramer possess MTS and the MCU tetramer will likely be trafficked to the mitochondria. If MCU-S expression is high, such as during MCU-S overexpression or in human platelets, MCU tetramers do not specifically traffic to mitochondria and may be sorted into other membrane structures, such as PM.

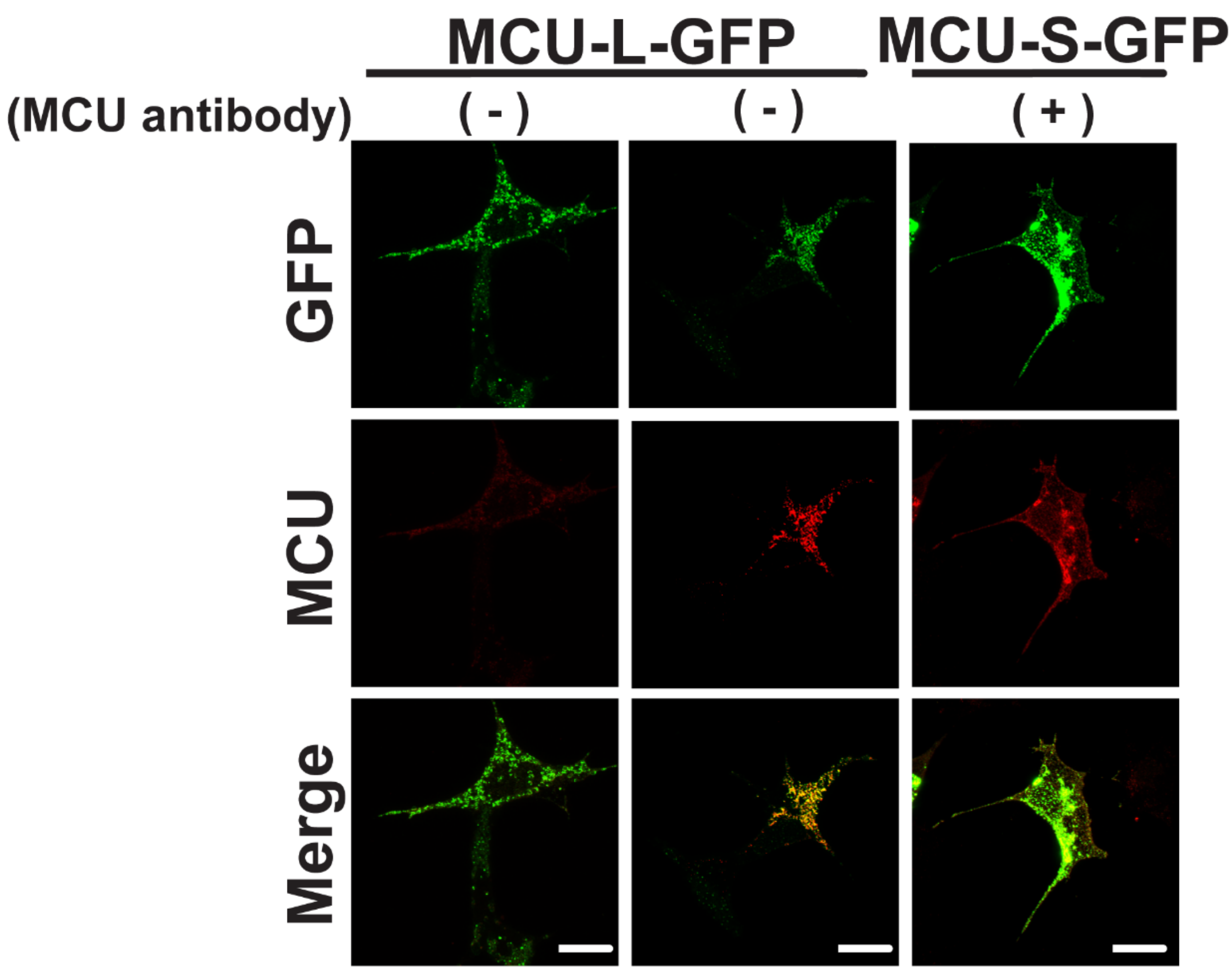

Supplementary Fig. 12. Specificity of MCU antibody used for immunocytochemistry.

HEK293T cells transiently overexpressing MCU-L-GFP and MCU-S-GFP were fixed and stained with a specific antibody against the internal region of human MCU (amino acid residues 110-129, see also Supplementary Table 4). Scale bar, 10 μm.

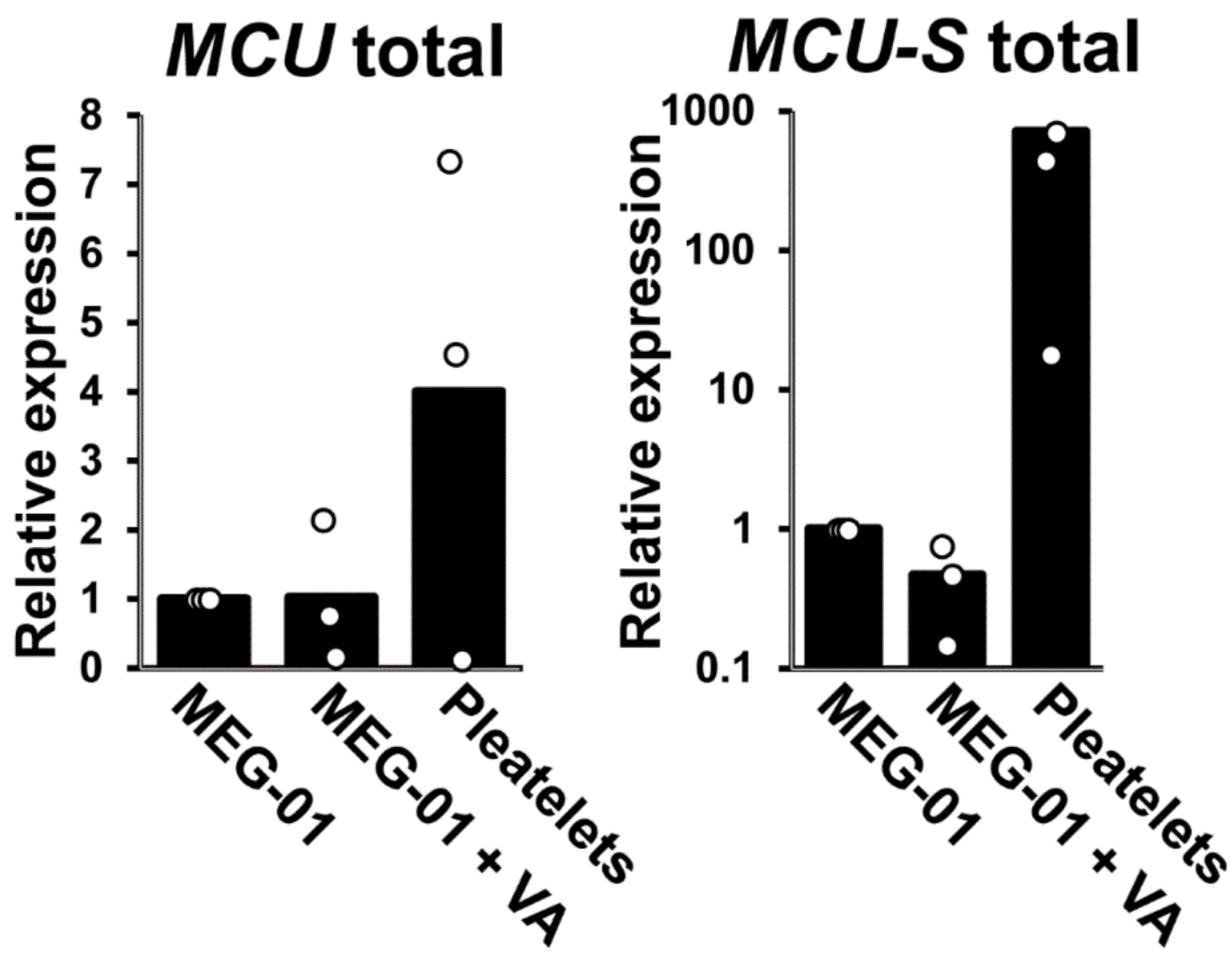

Supplementary Fig. 13. Effect of *in vitro* differentiation/maturation induction on MCU expression in MEG-01 cells.

RT-qPCR analysis of total *MCU* and *MCU-S* in MEG-01 cells before and after treatment with valproic acid (VA, 2 mM, 21 days) for maturation. The bar plots indicate mean values. *n*=3 for each group. Media containing VA was replaced every 72 hours. The data from human platelets were shown for comparison. MCU-S data is shown in visualizing in the logarithmic scale.

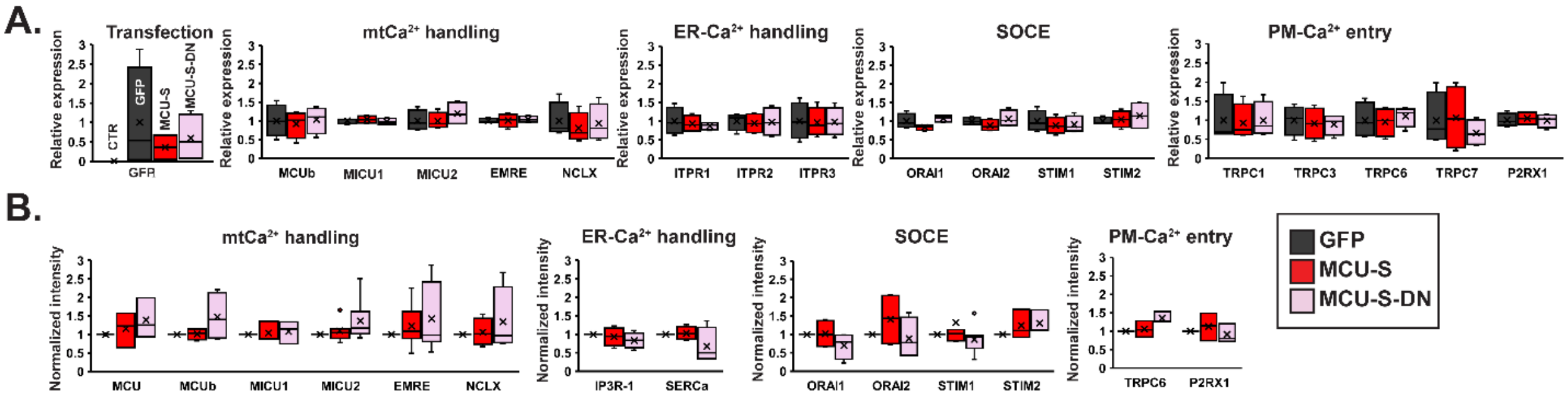

Supplementary Fig. 14. Effect of MCU-S expression on Ca^{2+} handling proteins in MEG-01 cells.

A. RT-qPCR analysis of major Ca^{2+} handling proteins in MEG-01 cells after transient transfection of MCU-S-GFP or MCU-S-DN-GFP. GFP was used as a control. CTR, non-transfected MEG-01 cells. Expression level in each condition was normalized to that in GFP-transfected cells. *n* = 4 for each group. Box & whiskers plots show the minimum and maximum values, 1st and 3rd quartiles, and the median. Mean average in each group was represented by “x” in the box. All the values for the plots were shown in Supplementary Table 7. **B.** Protein expression levels of major Ca^{2+} handling proteins in MEG-01 cells quantified by Western blotting. Tubulin was used as a loading control. All results were normalized to the value from the cells transfected with GFP. *n* (left to right) = 3, 4, 6, 4, 6, 4, 5, 4, 4, 4, 7, 3, 3, and 3. Box & whiskers plots show the minimum and maximum values, 1st and 3rd quartiles, and the median. Mean average in each group was represented by “x” in the box. All the values for the plots were shown in Supplementary Table 8.

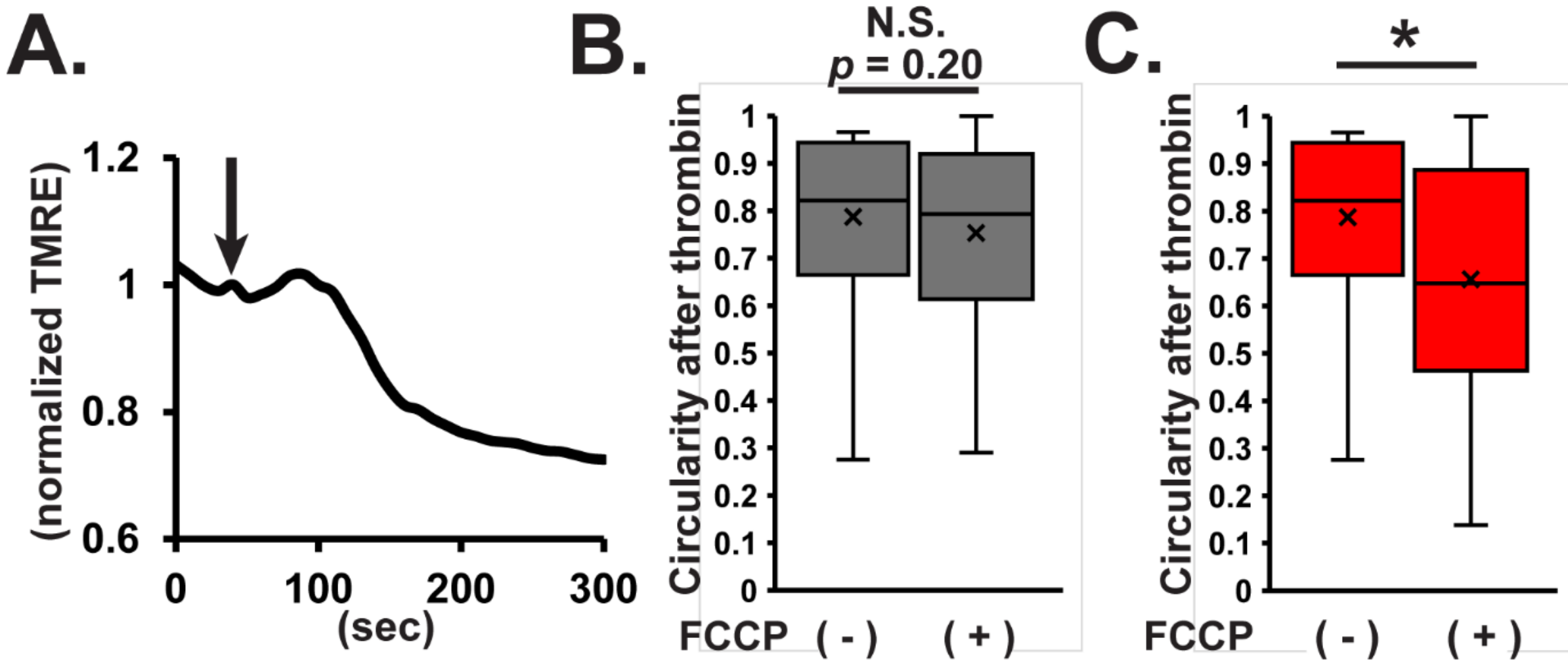

Supplementary Fig. 15. Effect of the mitochondrial uncoupler FCCP on thrombin-mediated morphological changes in MEG-01 cells.

A. Representative trace of the changes in the fluorescence intensity of mitochondrial membrane potential (Ψ_m)-sensitive dye TMRE in response to treatment with 500 nM FCCP in a single MEG-01 cell. The fluorescence value at each time point was normalized with the value before FCCP treatment. **B.** Effect of FCCP on thrombin-mediated morphological changes in MEG-01 cells. MEG-01 cells transiently transfected with GFP were incubated with 500 nM FCCP or vehicle (DMSO) for 5 minutes before thrombin stimulation. Morphological changes after thrombin stimulation were assessed in the glass-bottom micro dishes, as assessed by phase contrast images. Circularity reaches 1 if the cell is a perfect circle and becomes low in complex cell shapes. Box & whiskers plots show the minimum and maximum values, 1st and 3rd quartiles, and the median. Mean average in each group was represented by “x” in the box. *n* = 90 and 95; 1st quartile, 0.665 and 0.614; mean averages, 0.787 and 0.753; median values, 0.822 and 0.793; 3rd quartile, 0.944 and 0.920; maximum values, 0.966, and 1.000; and minimum values, 0.276 and 0.29, for FCCP (-), and FCCP (+) groups, respectively. Unpaired T-test. N.S., not significant. **C.** Effect of FCCP on thrombin-mediated morphological changes in MEG-01 cells overexpressing MCU-S. MEG-01 cells, transiently transfected with MCU-S-GFP, were incubated with 500 nM 00 nM FCCP (*n* = 90) or vehicle (DMSO, *n* = 91) for 5 minutes before thrombin stimulation. *n* = 91 and 217; 1st quartile, 0.611 and 0.464; mean averages, 0.730 and 0.657; median values, 0.732 and 0.648; 3rd quartile, 0.879 and 0.887; maximum values, 0.966 and 1.000; and minimum values, 0.251 and 0.138, for FCCP (-), and FCCP (+) groups, respectively. Unpaired T-test: *p<0.05.

Fig.1 D (top)

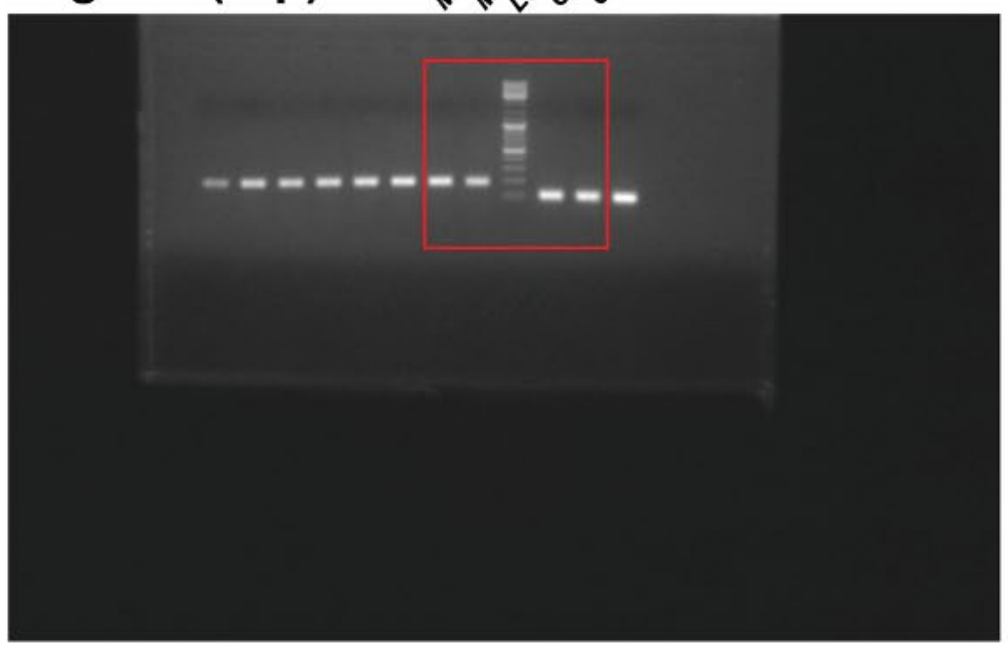

Fig.1 D (bottom)

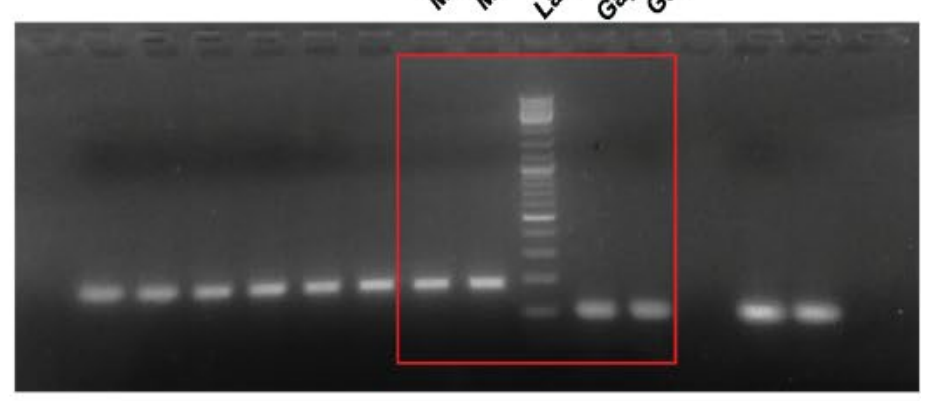

Fig.1 E

Antibody A **Antibody B** **Tubulin**

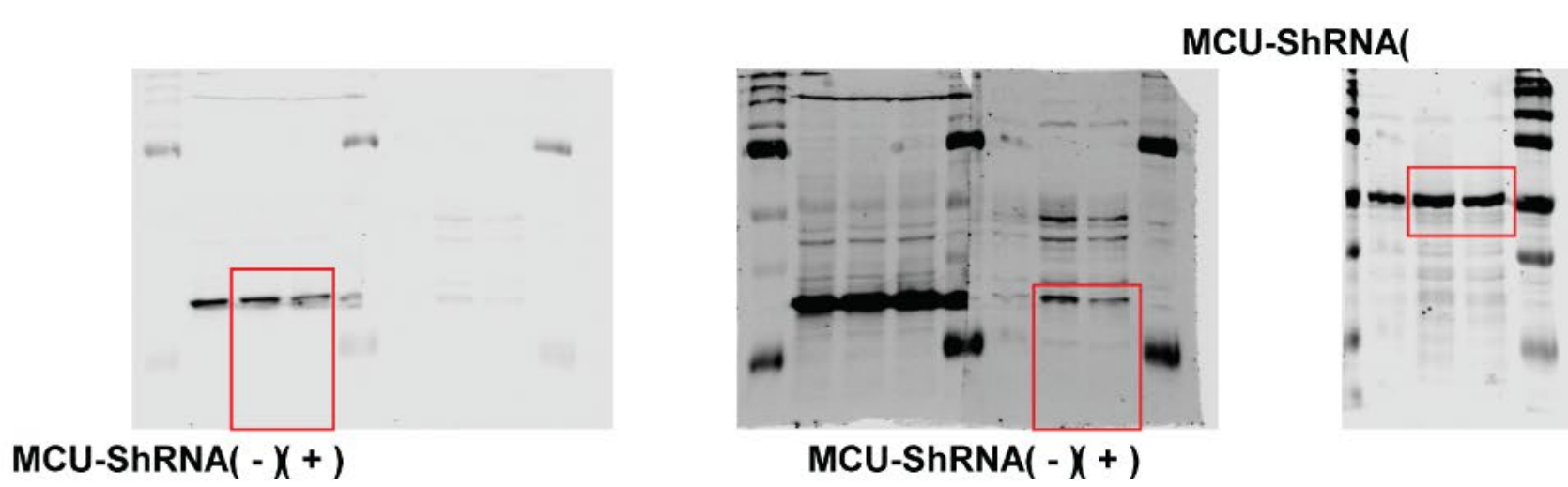

Fig.1G

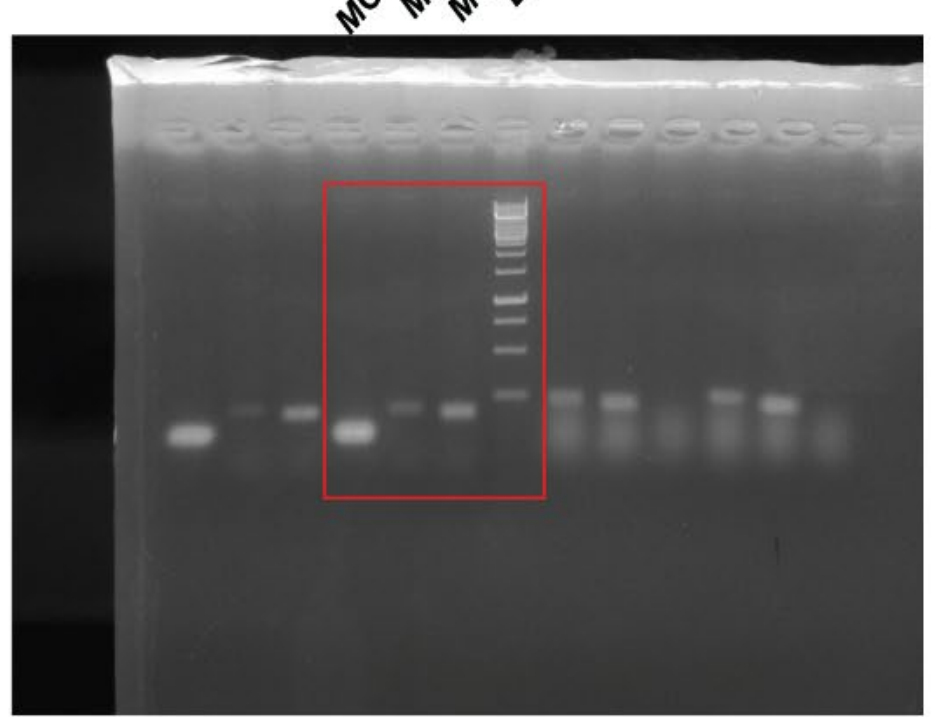

Supplementary Fig. 16. Uncropped gel and Western blotting images for Fig. 1

Uncropped gel images for Fig. 1D and G and Western blotting images for Fig. 1E are shown.

Fig. 2F

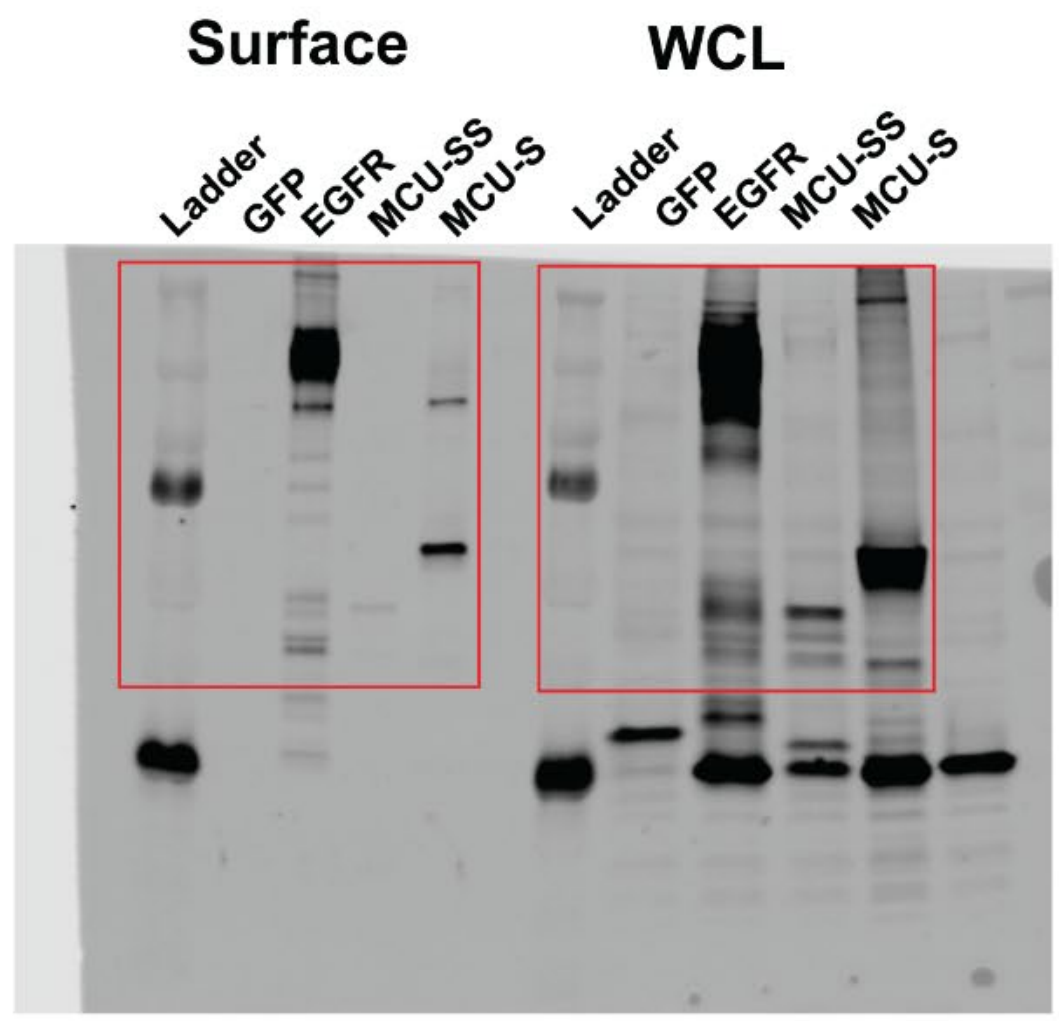

Fig. 3A

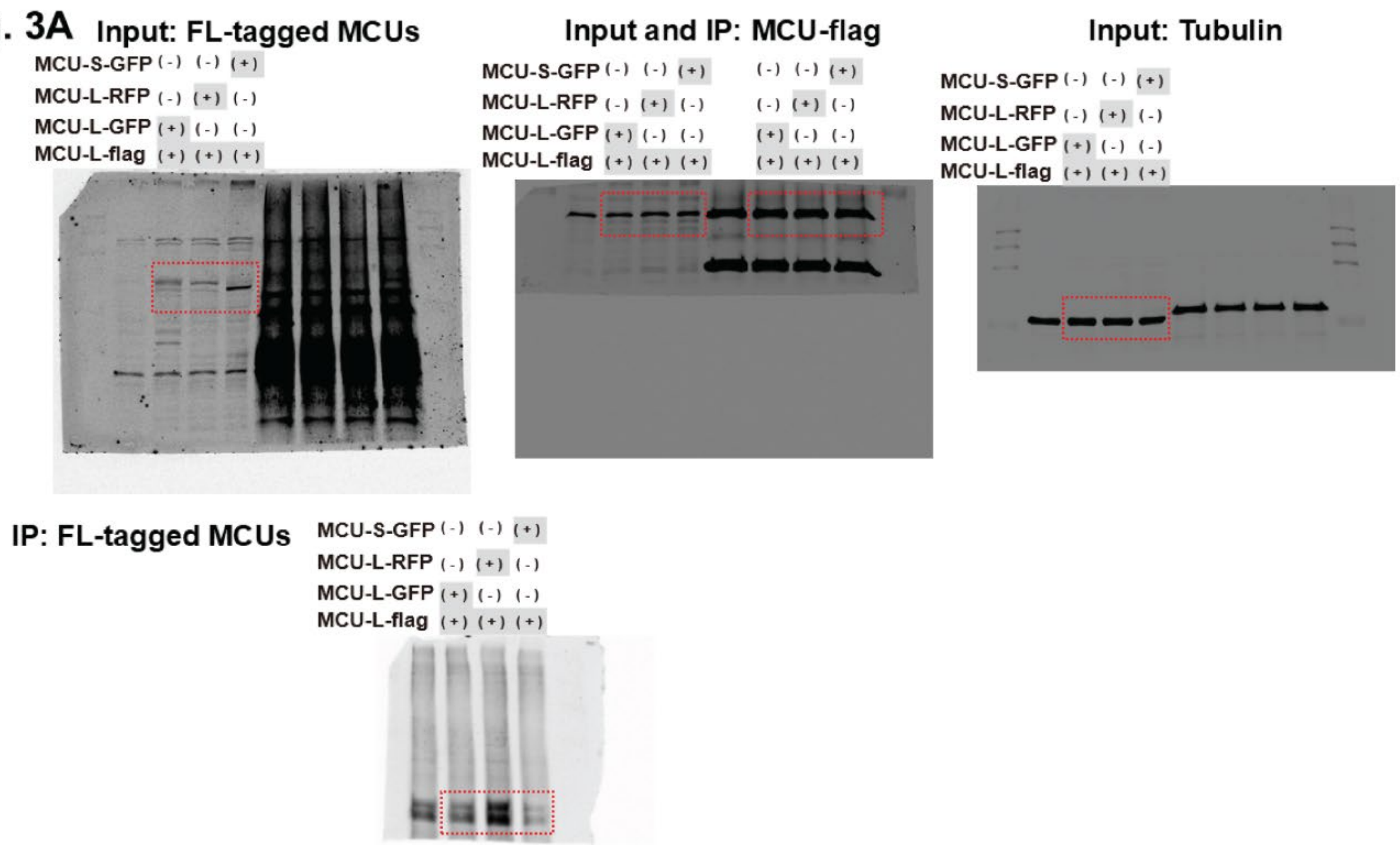

Supplementary Fig. 17. Uncropped Western blotting images for Figs. 2 and 3.

Uncropped Western blotting images for Figs. 2F and 3 A are shown.

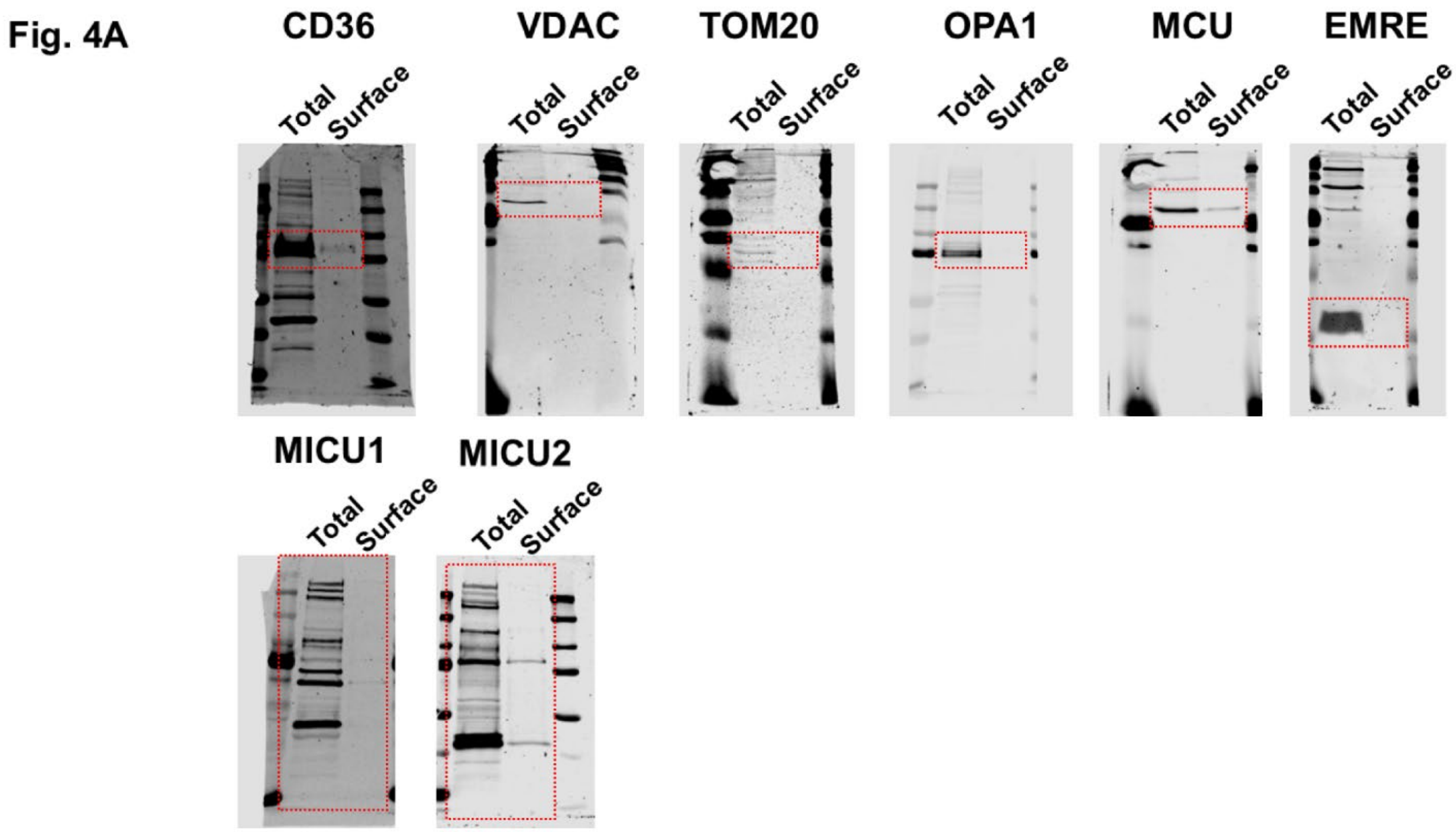

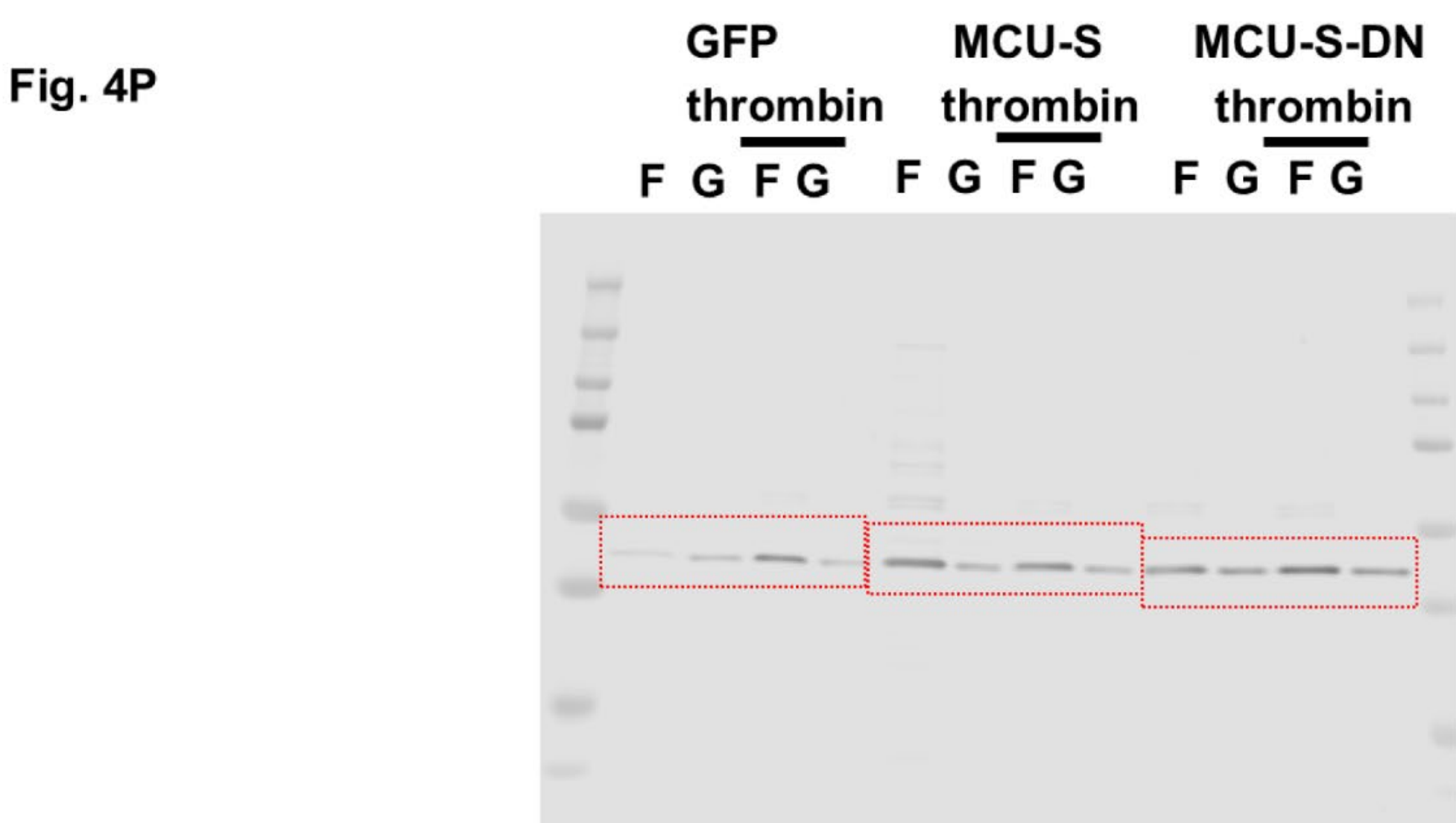

Supplementary Fig. 18. Uncropped Western blotting images for Fig. 4.

Uncropped Western blotting images for Fig. 4A and P are shown.

Supplementray Fig. 5

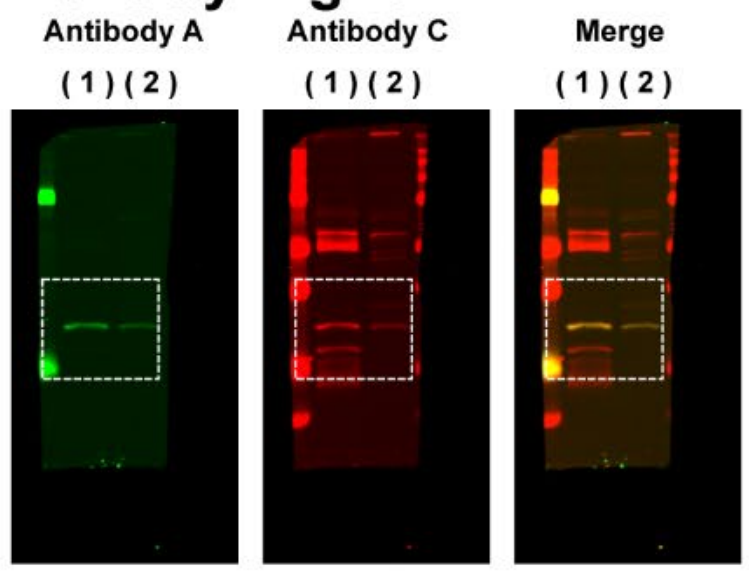

Supplementray Fig. 6A

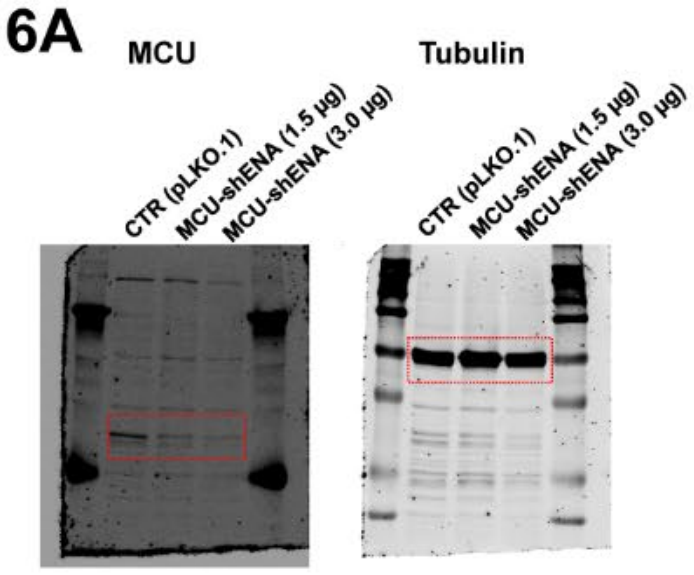

Supplementary Fig. 11

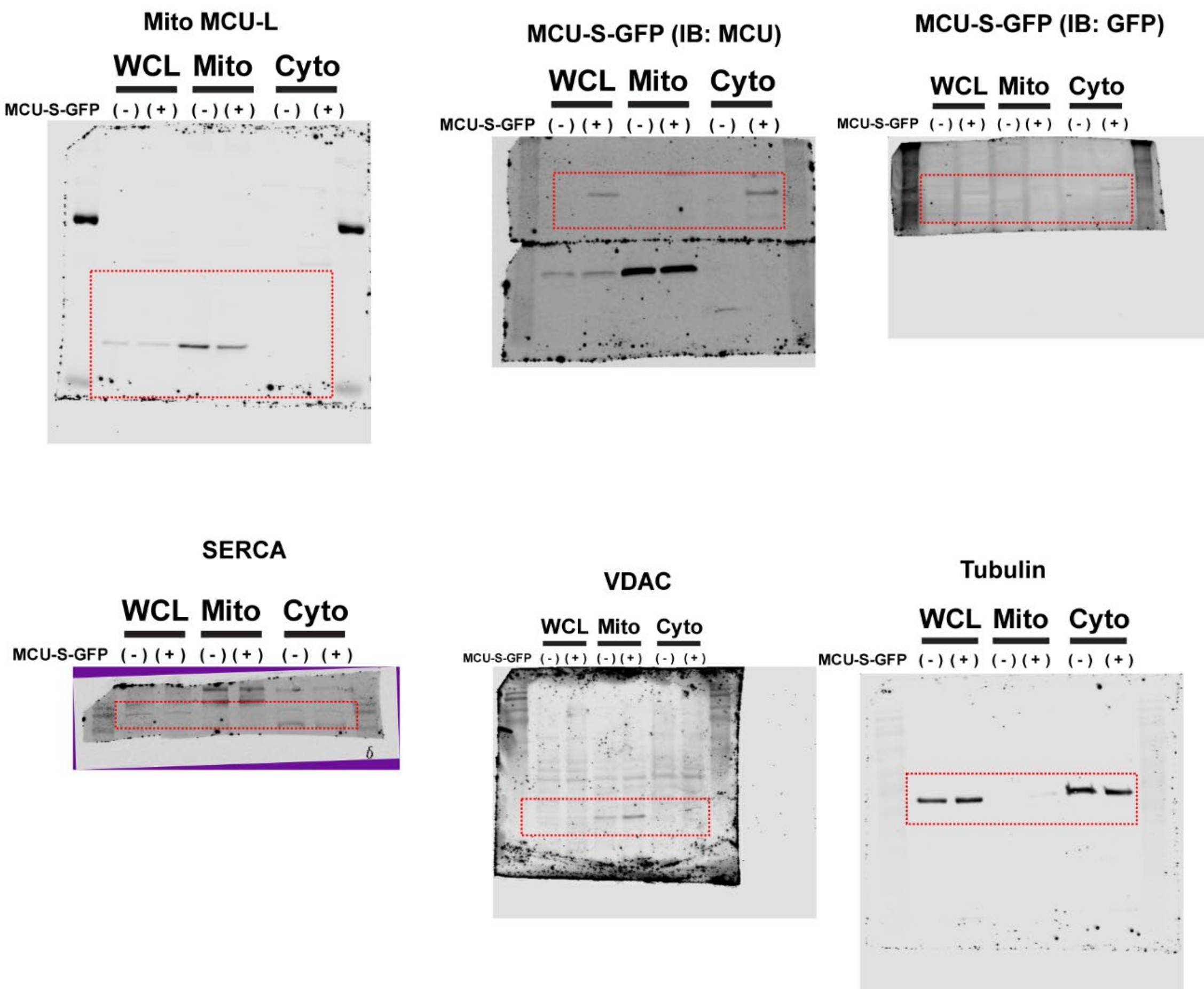

Supplementary Fig. 19. Uncropped Western blotting images for Supplementary Figures.

Uncropped Western blotting images for Supplementary Figs. 5, 6A and 11A are shown.

Supplementary Table 1. Molecular features of MCU variants.

Mitochondrial transport prediction was assessed by MitoProt 2 (https://ihg.helmholtz-muenchen.de/ihg/mitoprot.html) [2]. Red texts show the information regarding the short MCU variants.

MCU member	Specifc 5' exon	Promoter	Exons	Transcripts (bp)	Protein (AA)	Protein (kDa)	MTS size (kDa)	Mito transport prediction
human MCU-L	1	1	8	2949	351	39.9	5.4	0.9848
human MCU-L2	1	1	8	2874	330	37	5.4	0.7344
human MCU-S	2	2	8	3224	302	35.2	not predicted	0.1215
mouse MCU-L	1	1	8	2872	350	39.7	5.3	0.7307
mouse MCU-SS	5	2	5	1079	201	23.9	not predicted	0.5353

Supplementary Table 2. Primers for MCU variants.

Gene	Transcript ID (Ensembl)	Primer	Sequence	Product size (bp)
HsMCU-L	ENST00000373053.7	Forward	5' ATATTCCTGGGACATCATGGAG 3'	104
		Reverse	5' GGATAAACATATTCCTGGCGTG 3'	
HsMCU-L2	ENST00000357157.10	Forward	5' GCTGCTTCAACAGGAATAGAC 3’	200
		Reverse	5' CTCAATTCGTACGGCTATAATCTC 3'	
HsMCU-S	ENST00000536019.5	Forward	5' AGCGAATATGGTACACCAGAG 3'	181
		Reverse	5' CAGAGTCAGAGATAGGCTTGAG 3'	
MmMCU-L	ENSMUST00000020312.12	Forward	5' TACGACAACTGCAAGAGGAG 3’	181
		Reverse	5' ATCTTCATGGCTTAGGAGGTC 3'	
MmMCU-SS	ENSMUST00000009791.6	Forward	5' TTCGATCTGGGAAAGGACTG 3'	180
		Reverse	5' TTTGGCCTCAGTATCATGCT 3'	

Supplementary Table 3. Primers for Ca^{2+} handling genes in MEG-01 cells.

Function	Species	Gene Name	NCBI Gene ID	FWD Sequence (5'-3')	REV Sequence (5'-3')
**Housekeeping gene**	mouse	GAPDH	14433	5'-CAC CAT CTT CCA GGA GCG AG-3'	5'-CAA AGT CCC AGG CAG TTT CT-3′
	Human	GAPDH	2597	5'-TCG GAG TCA ACG GAT TTG-3'	5'-CAA CAA TAT CCA CTT TAC CAG AG-3′
**Reporter gene**	Aequorea victoria	GFP		5'-AAG CTG ACC CTG AAG TTC ATC TGC-3'	5'-CTT GTA GTT GCC GTC GTC CTT GAA-3′
**$mtCa^{2+}$ influx/efflux**	Human	MCUb	55013	5′-GGC CTT CCC TTG GTA ACA CT-3'	5'-GTT GCC ATC TGC TGT GAA GA-3′
	Human	MICU1	10367	5'-GAG GCA GCT CAA GAA GCA CT-3'	5'-CAA ACA CCA CAT CAC ACA CG-3’
	Human	MICU2	221154	5'-GGC AGT TTT ACA GTC TCC GC-3'	5'-AAG AGG AAG TCT CGT GGT GTC-3’
	Human	EMRE	91689	5'-TGT CGG GAC ACT CAT TAG CA-3'	5'-GCT GAT AGG GAA GGC AGA GA-3’
	Human	NCLX	80024	5'-GCC AGC ATT TGT GTC CAT TT-3'	5'-AAT TCG TCT CGG CCA CTT AC-3'
**SOCE**	Human	ORAI1	84876	5'-GAT GAG CCT CAA CGA GCA CT-3'	5'-ATT GCC ACC ATG GCG AAG C-3'
	Human	ORAI2	80228	5'-TGG CGG AAG CTC TAC CTG AG-3'	5'-CGG GTA CTG GTA CTG CGT C-3'
	Human	STIM1	6786	5'-AGT GAG GAT GAG AAA CTC AG-3'	5'-GAA CTC ATC ACT TTC TTC CAC-3'
	Human	STIM2	57620	5'-CCC TCA CCA CCC GCA ACA-3'	5'-GAT GTG TGG CGA GGT TTA GGC-3'
**ER Ca^{2+} releasing sites**	Human	ITPR1	3708	5'-AAG GAA AGA AAC CCT TGA CTG-3'	5'-CTG ATC CTT TAA TTC CGA CAG-3'

	Human	ITPR2	3709	5'-CTG TGT GGA AGA GCA ACT AC-3'	5'-ATG GGT CTG TAG GTA GGA ATA G-3'
	Human	ITPR3	3710	5'-CAA GCC CTC CAA AGA TGA G-3'	5'-CGA AGG TGT CGA TGA TTA CC-3'
**Plasma Membrane Ca^{2+} influx**	Human	TRPC1	7220	5'-TGC AGC TTC TTT TGG ACT ACG-3'	5'-CGA TGA GCA GCT AAA ATG ACA-3'
	Human	TRPC3	7222	5'-GAT CGC ACC TTG CAG CAG GC-3'	5'-AGG CAT TGA ACA CAA GCA GAC CC-3'
	Human	TRPC6	7225	5'-TTC ACA ACA GTT GAA GAG AG-3'	5'-GGA ATG AAC TGT TGA TCA TGG-3'
	Human	TRPC7	57113	5'-ACA AGT GGT GGC CTT CAG AC-3'	5'-CGT TGG CTG GCA GAA TGT AT-3'
	Human	P2RX1	5203	5'-ATG GTG CTG GTG CGT AAT AAG-3'	5'-GGA AGA CGT AGT CAG CCA CA-3'

Supplementary Table 4. List of primary antibodies used in this study.

Targeted Protein	Type	Company	Catalog number	Immunogen
GFP	Mouse monoclonal	Roche, Mannheim Germany and Sigma-Aldrich, St Louis, MO, USA	11814460001	Recombinant Aequorea victoria GFP
GFP	Rabbit monoclonal	Cell Signaling• Technology (CST), Danvers, MA, USA	2956	Monoclonal antibody is produced by immunizing animals with a synthetic peptide corresponding to the amino terminus of GFP
Tubulin	Mouse monoclonal	Sigma-Aldrich	T5168	Sarkosyl-resistant filaments from Strongylocentrotus purpuratus (sea urchin) sperm axonemes
Anti-voltage-dependent anion channel (VDAC)	Rabbit polyclonal	CST	4866S	A synthetic peptide corresponding to the amino terminus of human VDAC-1
Anti-flag	Rabbit polyclonal	CST	14793S	A synthetic DYKDDDDK peptide
Anti-flag	Mouse monoclonal	Sigma-Aldrich	F1804	A synthetic DYKDDDDK peptide
Anti-Argonaute 2 (Argo2)	Rabbit monoclonal	CST	2897S	A synthetic peptide corresponding to mouse Argonaute 2
Anti-MCU	Rabbit polyclonal	Sigma-Aldrich	HPA016480	N-term region of Human MCU (HHRTVHQRIASWQNLGAV YCSTVVPSDDVTVVYQNGL PVISVRLPSRRERCQFTLKP ISDSVGVFLRQLQEEDRGID RVAIYSPDGVRVAASTGIDL LLLDDFKLV). Antibody A in Figure 2E and in

				Supplementary Figure 4.
Anti-MCU	Rabbit polyclonal	Biorbyt, Cambridge, UK	orb185533	NDLTYHVRPPKRDLLS<br>Antibody B in Figure 2E.
Anti-MCU	Mouse monoclonal	Santa Cruz Biotechnology, Dallas, TX, USA	sc-515930	Epitope mapping between amino acids 110-129 within an internal region<br>of CCDC109A of human origin<br>Antibody C in Supplementary Figure 4.
Anti-SERCA1/2/3	Mouse monoclonal	Santa Cruz Biotechnology	sc-271669	Raised against amino acids 1-300 mapping at the N-terminus of SERCA1 of human origin.
MCUb	Rabbit polyclonal	Proteintech, Rosemont, IL, USA	20387-1-AP	MLQRGLWPWRTRLLPTPG TWRPARPWPLPPPPQVLR VKLCGNVKYYQSHHYSTVV PPDEITVIYRHGLPLVTLTLP SRKERCQFVVKPMLSTVGS FLQDLQNEDKGIKTAAIFTA DGNMISASTLMDILLMNDFK LVINKIAYDVQCPKREKPSN EHTAEMEHMKSLVHRLFTIL HLEESQKKREHHLLEKIDHL KEQLQPLEQVKAGIEAHSE AKTSGLLWAGLALLSI
MICU1	Rabbit polyclonal	Sigma-Aldrich	HPA037480	LKGKLTIKNFLEFQRKLQHD VLKLEFERHDPVDGRITER QFGGMLLAYSGVQSKKLTA MQRQLKKHFKEGKGLTFQ EVENFFTFL
MICU2	Rabbit polyclonal	Abcam, Cambridge, UK	ab101465	Recombinant fragment corresponding to Human MICU2 aa 95-238
EMRE	Rabbit polyclonal	Santa Cruz Biotechnology	sc-86337	A peptide mapping at the C-terminus of C22orf32 of human origin
NCLX	Rabbit	Proteintech	21430-1-AP	YVVTVILCTWIYQRQRRGS LFCPMPVTPEILSDSEEDRV

	polyclonal			SSNTNSYDYGDEYRPLFFY QETTAQILVRALNPLDYMK WRRKSAYWKALKVFKLP
Orai1	Mouse monoclonal	Santa Cruz Biotechnology	sc-377281	Amino acids 256-301 mapping at the C-terminus of Orai1 of human origin
Orai 2	Mouse monoclonal	Santa Cruz Biotechnology	sc-376757	Amino acids 1-49 mapping at the N-terminus of Orai2 of human origin
STIM1	Mouse monoclonal	Santa Cruz Biotechnology	sc-166840	Amino acids 441-620 mapping near the C-terminus of Stim1 of human origin
STIM2	Rabbit polyclonal	Proteintech	21192-1-AP	EKICGFQIAHNSGLPSLTSS LYSDHSWVVMPRVSIPPYP IAGGVDDLDEDTPPIVSQFP GTMAKPPGSLARSSSLCRS RRSIVPSSPQPQRAQLAPH APHPSHPRHPHHPQHTPH SLPSPDPDILSVSSCPALYR NEEEEEAIYFSAEKQ
IP_3 receptor type 1	Rabbit polyclonal	Alomone Lab, Jerusalem, Israel	ACC-019	Peptide (C)RIGLLGHPPHMNVNPQQPA, corresponding to amino acid residues 2732-2750 of rat IP3R1
P2RX1	Rabbit polyclonal	Aviva Systems Biology, San Diego, CA, USA	ARP35105_T100	VVGITIDWHCDLDWHVRHC RPIYEFHGLYEEKNLSPGF NFRFARHFVENG
TRPC6	Rabbit polyclonal	Novus Biologicals, Centennial, CO, USA	NBP1-77260	Antibody was raised against a 14 amino acid synthetic peptide from near the carboxy terminus of human TRPC6 (LEEKSQNTEDLAEL).
β-actin	Mouse monoclonal	Abclonal, Woburn, MA, USA	AC004	Whole sequence of human β-actin.

actin	Mouse monoclonal	Developmental Studies Hybridoma Bank (DSHB). JLA20 was deposited to the DSHB by Lin, J.J.-C. [3]	JLA20	Chicken actin
TOM20	Mouse monoclonal	BD Bioscience, Franklin Lakes, NJ, USA	612278	Human Tom20 aa. 47-145
TOM20	Rabbit polyclonal	Proteintech	11802-1-AP	MVGRNSAIAAGVCGALFIG YCIYFDRKRRSDPNFKNRL RERRKKQKLAKERAGLSKL PDLKDAEAVQKFFLEEIQLG EELLAQGEYEKGVDHLTNA IAVCGQPQQLLQVLQQTLP PPVFQMLLTKLPTISQRIVS AQSLAEDDVE
CD36 (Thrombospondin Receptor)	Mouse monoclonal	Proteintech	66395-1-Ig	1-472 aa encoded by BC008406
OPA1	Mouse monoclonal	BD Biosciences	612607	Human OPA1 aa. 708-830

Supplementary Table 5. List of plasmids used in this study.

No.	Inserted gene	Vector Backbone	Source /Provider/Company	Notes	References
**1**	Mitochondrial matrix-targeted DsRed (mt-RFP)	pDsRed1-N1	Dr. Yisang Yoon		4
**2**	Mouse MCU-L-GFP	pEGFP-N1	Dr. Rosario Rizzuto		5
**3**	Mouse MCU-L-flag	pcDNA 3.1 (+)	Dr. Rosario Rizzuto		5
**4**	Mouse MCU-L-DsRed	pDsRed1-N1		Generated from plasmid No.2 by replacing the plasmid backbone.	6
**5**	Mouse MCU-SS	pCR4-TOPO	Source BioScience, Nottingham, UK	(GenBank: BC139257.1, cDNA clone MGC:170884 IMAGE:8862279, termed mMCU-SS)	
**6**	Mouse MCU-SS-flag	pcDNA 3.1 (+)		Generated from plasmid No.5. N-term part of mouse MCU-L in plasmid 3 was replaced by a part of plasmid 5.	
**7**	Mouse MCU-SS-GFP	pEGFP-N1		Generated from plasmid No.5. N-term part of Mouse MCU-L in plasmid 2 was replaced by a part of plasmid 5.	
**8**	TurboGFP-tagged human MCU3	pCMV6-AC-GFP	OriGene Technologies, Rockville, MD, USA	(NM_001270680, termed hMCU-S)	

**9**	hMCU-S-EGFP	pEGFP-N1		Generated from plasmid No.8 by subcloning hMCU-S into pEGFP-N1	
**10**	Mitochondrial matrix-targeted Ca^{2+} biosensor, mt-RCaMP1h		Drs. Jacopo Fontana and Anita Aperia		[7]
**11**	MCU-S-DN-GFP	pEGFP-N1	VectorBuilder, Chicago, IL, USA	Mutating negatively charged residues of the conserved region in the MCU-S pore (D260, E263 in MCU-L) [5] into glutamines. Plasmid No.9 was used as a template.	
**11**	N/A	pEGFP-N1	Clonetech, Mountain View, CA, USA		
**12**	N/A	pLKO.1 puro	Addgene, Cambridge, MA, USA, Dr. Bob Weinberg	#8453	[8]
**13**	EGFR-GFP	pEGFP-N1	Addgene, Dr. Alexander Sorkin	#32751	[9]
**14**	chick src Y527F (constitutive active mutant c-Src)	pLNCX	Addgene, Dr. Joan Brugge	#13660	
**15**	shRNA targeted to the 3' UTR of mouse MCU	pLKO.1 puro	Sigma-Aldrich	MISSION shRNA TRCN0000251263	

**16**	shRNA targeted to the 3' UTR of human MCU	pLKO.1 puro	Sigma-Aldrich	MISSION shRNA TRCN0000133861	
**17**	MCU-mCherry	pmCherry N1	Addgene, Dr. Giulia Bertolin	#232790	[10]
**18**	Smac (1-60) mCherry (human DIABLO)	pcDNA3.1	Addgene, Dr. Douglas Green	# 40880	[11]
**19**	N/A	pcDNA3.1(+)	Invitrogen, Grand Island, NY, USA		
**20**	N/A	pCMV6-Entry	OriGene Technologies	# PS100001	

Supplementary Table 6. List of platelets used for this study.

Company	Sex	Age(s)	Ethnicity
Zenbio/American Blood Bank, Durham, NC, USA	F	63	African American
Zenbio/American Blood Bank	F	50	unknown
Zenbio/American Blood Bank	F	56	Hispanic
HumanCells Biosciences, Milpitas, CA, USA	M	59	Caucasian
HumanCells Biosciences	M	33	African American
HumanCells Biosciences	M	31	African American
HumanCells Biosciences	M	52	Hispanic
HumanCells Biosciences	M	48	Caucasian

Supplementary Table 7. List of key parameters in Supplementary Fig.14A.

		Quartile 1	Average	Median	Quartile 3	Maximum	Minimum
**Transfection**	**CTR**	5.8E-11	9.42E-10	6.32E-10	2.14E-09	2.49E-09	1.77E-11
**Transfection**	**GFP**	0.042559	1	0.535921	2.421519	2.886104	0.042053
**Transfection**	**MCU-S-GFP**	0.047513	0.360041	0.363139	0.669471	0.677305	0.03658
**Transfection**	**MCU-S-DN-GFP**	0.07486	0.592919	0.499683	1.204214	1.300399	0.071911
**MCUb**	**GFP**	0.604076	1	0.984026	1.411898	1.532928	0.49902
**MCUb**	**MCU-S-GFP**	0.542006	0.918572	1.015069	1.198642	1.234754	0.409397
**MCUb**	**MCU-S-DN-GFP**	0.663896	1.031112	1.104425	1.325013	1.366389	0.549207
**MICU1**	**GFP**	0.929223	1	1.031373	1.039404	1.039445	0.897809
**MICU1**	**MCU-S-GFP**	0.942938	1.034569	1.030767	1.130002	1.148614	0.928127
**MICU1**	**MCU-S-DN-GFP**	0.91234	0.980322	0.960595	1.068031	1.091904	0.908194
**MICU2**	**GFP**	0.779845	1	0.938826	1.281329	1.371693	0.750654
**MICU2**	**MCU-S-GFP**	0.829676	0.990955	0.92175	1.221439	1.317994	0.802325
**MICU2**	**MCU-S-DN-GFP**	0.926489	1.19303	1.16237	1.490232	1.52428	0.923102
**EMRE**	**GFP**	0.954976	1	0.980197	1.064827	1.087091	0.952515
**EMRE**	**MCU-S-GFP**	0.833201	1.010362	1.025949	1.171938	1.21191	0.777642
**EMRE**	**MCU-S-DN-GFP**	0.963533	1.036446	1.027143	1.118662	1.136171	0.955328
**NCLX**	**GFP**	0.709586	1	0.801193	1.489222	1.714122	0.683492
**NCLX**	**MCU-S-GFP**	0.519645	0.804769	0.681454	1.213208	1.386437	0.469731
**NCLX**	**MCU-S-DN-GFP**	0.53683	0.928788	0.803954	1.445579	1.620597	0.486646
**ITPR1**	**GFP**	0.68053	1	0.955157	1.364314	1.470477	0.619209
**ITPR1**	**MCU-S-GFP**	0.737214	0.929804	0.901338	1.150859	1.194057	0.722482
**ITPR1**	**MCU-S-DN-GFP**	0.7697	0.87179	0.893594	0.952076	0.952344	0.747629
**ITPR2**	**GFP**	0.757084	1	1.08744	1.155477	1.169737	0.655383
**ITPR2**	**MCU-S-GFP**	0.692476	0.937802	0.927805	1.193125	1.217649	0.677949
**ITPR2**	**MCU-S-DN-GFP**	0.623947	0.977493	0.954408	1.354124	1.408088	0.593068
**ITPR3**	**GFP**	0.54913	1	0.971361	1.479509	1.617888	0.43939
**ITPR3**	**MCU-S-GFP**	0.646333	0.963737	0.886789	1.35809	1.505387	0.575985
**ITPR3**	**MCU-S-DN-GFP**	0.644845	0.97657	0.93432	1.350545	1.482275	0.555364
**ORAI1**	**GFP**	0.847305	1	0.952902	1.199792	1.276152	0.818043
**ORAI1**	**MCU-S-GFP**	0.760813	0.835797	0.850592	0.895985	0.901246	0.740758
**ORAI1**	**MCU-S-DN-GFP**	0.984014	1.075096	1.092993	1.148283	1.153011	0.961389
**ORAI2**	**GFP**	0.911765	1	0.987133	1.101102	1.12076	0.904973
**ORAI2**	**MCU-S-GFP**	0.757257	0.884365	0.852107	1.043731	1.080691	0.752556
**ORAI2**	**MCU-S-DN-GFP**	0.886761	1.063546	1.012446	1.29143	1.357106	0.872184
**STIM1**	**GFP**	0.772146	1	0.943762	1.284092	1.393291	0.719185
**STIM1**	**MCU-S-GFP**	0.683397	0.882985	0.865254	1.100304	1.175516	0.625916
**STIM1**	**MCU-S-DN-GFP**	0.739876	0.907593	0.84308	1.139822	1.222707	0.721503
**STIM2**	**GFP**	0.932182	1	0.962453	1.105364	1.143692	0.931402
**STIM2**	**MCU-S-GFP**	0.836374	1.049325	1.044307	1.267293	1.325904	0.782781
**STIM2**	**MCU-S-DN-GFP**	0.812758	1.14853	1.139869	1.492964	1.508468	0.805916
**TRPC1**	**GFP**	0.640238	1	0.686485	1.673277	1.98743	0.639599

**TRPC1**	**MCU-S-GFP**	0.622484	0.930395	0.743382	1.42532	1.623678	0.611139
**TRPC1**	**MCU-S-DN-GFP**	0.661249	0.999891	0.852307	1.486116	1.666903	0.628046
**TRPC3**	**GFP**	0.608165	1	1.052895	1.33894	1.416562	0.477647
**TRPC3**	**MCU-S-GFP**	0.524012	0.913694	0.906685	1.310384	1.388611	0.452794
**TRPC3**	**MCU-S-DN-GFP**	0.608016	0.892345	0.968799	1.100221	1.103907	0.527877
**TRPC6**	**GFP**	0.599286	1	0.932941	1.467774	1.573383	0.560736
**TRPC6**	**MCU-S-GFP**	0.581623	0.958722	0.995184	1.299361	1.341264	0.503258
**TRPC6**	**MCU-S-DN-GFP**	0.831056	1.106892	1.190699	1.298922	1.326728	0.719444
**TRPC7**	**GFP**	0.599286	1	0.932941	1.467774	1.573383	0.560736
**TRPC7**	**MCU-S-GFP**	0.581623	0.958722	0.995184	1.299361	1.341264	0.503258
**TRPC7**	**MCU-S-DN-GFP**	0.831056	1.106892	1.190699	1.298922	1.326728	0.719444
**P2RX1**	**GFP**	0.860751	1	0.964593	1.174656	1.24289	0.827924
**P2RX1**	**MCU-S-GFP**	0.898438	1.048591	1.038828	1.208508	1.218908	0.897802
**P2RX1**	**MCU-S-DN-GFP**	0.801625	0.990781	1.029472	1.141247	1.148684	0.755497

Supplementary Table 8. List of key parameters in Supplementary Fig.14B.

		Quartile 1	Average	Median	Quartile 3	Maximum	Minimum
**MCU**	**GFP**	1	1	1	1	1	1
**MCU**	**MCU-S-GFP**	0.647401	1.149491	1.229021	1.572051	1.572051	0.647401
**MCU**	**MCU-S-DN-GFP**	0.936478	1.391709	1.252152	1.986496	1.986496	0.936478
**MCUb**	**GFP**	1	1	1	1	1	1
**MCUb**	**MCU-S-GFP**	0.859151	1.0112	1.030395	1.144055	1.154449	0.829563
**MCUb**	**MCU-S-DN-GFP**	0.899563	1.476132	1.403964	2.12487	2.218581	0.878019
**MICU1**	**GFP**	1	1	1	1	1	1
**MICU1**	**MCU-S-GFP**	0.796637	0.971741	0.87783	1.240755	1.356785	0.774518
**MICU1**	**MCU-S-DN-GFP**	0.843104	1.433141	1.23987	2.216448	2.50873	0.744093
**MICU2**	**GFP**	1	1	1	1	1	1
**MICU2**	**MCU-S-GFP**	1.00304	1.153395	1.089648	1.27526	1.660202	0.894809
**MICU2**	**MCU-S-DN-GFP**	0.99127	1.173928	1.120827	1.347629	1.617285	0.907789
**EMRE**	**GFP**	1	1	1	1	1	1
**EMRE**	**MCU-S-GFP**	0.887115	1.222415	1.081589	1.620753	2.251661	0.489574
**EMRE**	**MCU-S-DN-GFP**	0.809786	1.422788	0.977622	2.42603	2.878232	0.522364
**NCLX**	**GFP**	1	1	1	1	1	1
**NCLX**	**MCU-S-GFP**	0.731789	1.061565	1.015535	1.43737	1.546798	0.668392
**NCLX**	**MCU-S-DN-GFP**	0.779357	1.346729	0.974381	2.286451	2.673514	0.764641
**IP3R-1**	**GFP**	1	1	1	1	1	1
**IP3R-1**	**MCU-S-GFP**	0.689389	0.932982	0.941824	1.167733	1.225451	0.622829
**IP3R-1**	**MCU-S-DN-GFP**	0.632439	0.834652	0.837853	1.033664	1.098097	0.564805
**SERCa**	**GFP**	1	1	1	1	1	1
**SERCa**	**MCU-S-GFP**	0.908745	1.061568	1.042159	1.042159	1.26316	0.827721
**SERCa**	**MCU-S-DN-GFP**	0.346395	0.698887	0.630744	0.630744	1.364032	0.329455
**ORAI1**	**GFP**	1	1	1	1	1	1
**ORAI1**	**MCU-S-GFP**	0.670532	1.014572	0.997616	1.375566	1.398344	0.66471
**ORAI1**	**MCU-S-DN-GFP**	0.322711	0.697627	0.793649	0.976521	0.985298	0.217913
**ORAI2**	**GFP**	1	1	1	1	1	1
**ORAI2**	**MCU-S-GFP**	0.74794	1.414525	1.437733	2.057901	2.070474	0.712159
**ORAI2**	**MCU-S-DN-GFP**	0.429707	0.890251	0.776615	1.46443	1.59185	0.415924
**STIM1**	**GFP**	1	1	1	1	1	1
**STIM1**	**MCU-S-GFP**	0.826277	1.322335	1.021901	1.127468	1.127468	0.806789
**STIM1**	**MCU-S-DN-GFP**	0.621519	0.861848	0.934895	0.971714	0.971714	0.318419
**STIM2**	**GFP**	1	1	1	1	1	1
**STIM2**	**MCU-S-GFP**	0.927936	1.238427	1.106383	1.680962	1.680962	0.927936
**STIM2**	**MCU-S-DN-GFP**	1.11731	1.302666	1.126955	1.663734	1.663734	1.11731
**TRPC6**	**GFP**	1	1	1	1	1	1
**TRPC6**	**MCU-S-GFP**	0.84507	1.053252	1.035146	1.27954	1.27954	0.84507
**TRPC6**	**MCU-S-DN-GFP**	1.256994	1.355168	1.27295	1.53556	1.53556	1.256994
**P2RX1**	**GFP**	1	1	1	1	1	1
**P2RX1**	**MCU-S-GFP**	0.741935	1.125737	1.147847	1.487429	1.487429	0.741935
**P2RX1**	**MCU-S-DN-GFP**	0.725806	0.91186	0.803302	1.206471	1.206471	0.725806

Supplementary Table 9. List of key chemicals and reagents used in this study.

Chemical /Reagent name	Source/Provider/Company	Product number
Di-8-ANEPPS	Santa Cruz Biotechnology	sc-214873
MG132	AbMol, Houston, TX, USA	M1902
thapsigargin	Alomone Labs	T-650
YM-58483	Selleck Chemicals, Houston, TX, USA	S8380
valproic acid	AmBeed, Arlington Heights, IL, USA	A143794
carbonyl cyanide-p-trifluoromethoxyphenylhydrazone (FCCP)	Focus Biomolecules, Plymouth Meeting, PA, USA	10-1182-0010
MitoSOX-Red	Thermo Fisher Scientific (TFS), Waltham, MA, USA	M36008
MitoTracker Deep Red FM (mtDRFM)	TFS	M22426
Fluo-3	Biotium, Fremont, CA, USA	50013
tetramethylrhodamine ethyl ester perchlorate (TMRE)	Biotium	70005
Fura-Red (Fura 2-TH-AM)	Setareh Biotech, Eugene, OR, USA	7198
Calcein Violet-AM	Biolegend, San Diego, CA	425203
prostaglandin E1	Cayman Chemical, Ann Arbor, MI, USA	13010
prostaglandin I2	Cayman Chemical	18220
Dulbecco's modified Eagle's medium (DMEM)	Cytiva, Marlborough, MA<br>Mediatech/Corning, Corning, NY, USA	SH30243.02<br>10-013-CM
fetal bovine serum (FBS)	GIBCO, Grand Island, NY, #26140095	26140095
100 U/ml penicillin, and 100 μg/ml streptomycin	Genesee Scientific, San Diego, CA, USA<br>Mediatech/Corning	25-512<br>30002CI
100 μg/ml streptomycin and 0.25 μg/mL Fungizone	GIBCO	15240062
RPMI 1640 medium	Cytiva<br>Genesee Scientific	SH30027.02<br>25-506
G-418	Mediatech/Corning	61-234-RK

puromycin	GeminiBio Products, West Sacramento, CA, USA	400-128P
collagenase type 2	Worthington Biochemical Corp., Lakewood, NJ, USA	LS004177
DMEM/F12	TFS	11320033
Fugene HD transfection reagent	Promega, Madison, WI, USA<br>Fugent LLC, Middleton, WI, USA	E2312<br>HD-5000
RNeasy® Mini Kit	Qiagen Inc., Germantown, MD, USA	74134
TRIzol™	TFS	15596026
DNase	Zymo Research, Irvine CA, USA	E1010
RNA Clean & Concentrator-5 kit	Zymo Research	R1013
iScript™ reverse transcription kit	Bio-Rad Laboratories, Hercules, CA, USA	1708840
Superscript™ IV VILO™ Master Mix	TSF	11756050
ethidium bromide	TSF	J66192.03
QIAquick gel extraction kit	Qiagen	28704
Fast SYBR Green Master Mix	TFS	4385612
PowerUp SYBR Green Master Mix	TFS	A25742
Lysis Buffer	CST	9803S
PMSF	TCI America, Portland, OR, USA	B1062
Protein A/G Plus Agarose	Santa Cruz Biotechnology	sc-2003
cytochalasin D	Focus Biomolecules	10-2071-0001
nitrocellulose membranes	Bio-Rad Laboratories<br>Genesee Scientific<br>Santa Cruz Biotechnology	1620115<br>84-876<br>sc-3724
fluorescence-conjugated secondary antibodies for Western Blotting	LI-COR Biotechnology, Lincoln, NE, USA	926-32211 and 925-68020

Pierce Cell Surface Protein Isolation Kit	TFS	89881
Pierce Cell Surface Biotinylation and Isolation Kit	TFS	A44390
EZ-Link Sulfo-NHS-SS-Biotin	TFS	PI21331
NeutrAvidin agarose (TFS)	TFS	29201
Pierce Lane Marker Non-Reducing Sample Buffer	TFS	39001
dithiothreitol	TFS	A39255
Cell-Tak	Corning	354241
poly-L-lysine	Electron Microscopy Sciences, Hatfield, PA, USA	19320-B
paraformaldehyde	TFS	PI28908
goat serum	CST	5425
fluorescence-conjugated secondary antibodies for immunocytochemistry	TFS	A-11034 and A-11019
PROTEOSTAT® Aggresome detection kit	Enzo Biochem, Farmingdale, NY, USA	ENZ-51035
Triton X-100	TFS	28314
acetone	Neta Scientific, Hainesport, NJ	OAK-035905-500ml
CF568-conjugated phalloidin	Biotium	00044-T
DAPI	Biotium	40043

Supplementary Table 10. List of cell lines used in this study.

Cell line name	Source/Provider/Company	Product number
HEK293T	Dr. Keigi Fujiwara, UT MD Anderson Cancer Center, Houston TX, USA	
K562	American Type Culture Collection (ATCC), Manassas, VA, USA	CCL-243
Jurkat	ATCC	TIB-152
Raji	ATCC	CCL-86
MEG-01	ATCC	CRL-2021
HeLa	ATCC	CRM-CCL-2
C2C12	Dr. Robert T Dirksen, University of Rochester, Rochester NY, USA	
HEK293T stably overexpressing MCU-L-Flag	Developed in the authors' lab [6].	
HEK293T stably overexpressing pcDNA3.1(+)	Developed in the authors' lab [6].	
HEK293T stably overexpressing EGFR	Developed in the authors' lab [12].	
HEK293T stably overexpressing EGFP	Developed in the authors' lab [12].	

Supplementary Table 11. List of total human cDNA used for this study.

Tissue	Company	Catalog #	Lot #	Donor #	Sex	Age(s)	Medical Notes
Heart	Biochain, Newark, CA, USA	R1234138-50	B711068	1	M	27	Normal Left Ventricle
Heart	Takara, San Jose, CA, USA	636532	2008105A	3	M, M, M	30, 30, 39	Trauma
Heart	Zyagen, San Diego, CA, USA	HR-801		1	M	55	
Lung	Biochain	R1234152-50	C502069	1	M	27	Normal
Lung	Takara	636524	2105090	4	F, M, M, M	24-66	N/A
Lung	Zyagen	HR-601		1	M	57	
Liver	Biochain	R1234149-50	B705065	1	M	64	Normal
Liver	Takara	636531	2103225	4	M/F	26, 64, 70, 78	N/A
Liver	Zyagen	HR-314		1	M	45	
Skeletal Muscle	Biochain	R1234171-50	B902003	1	M	24	Normal
Skeletal Muscle	Takara	636534	1812055	4	M/F	24, 30, 78, 87	N/A
Skeletal Muscle	Zyagen	HR-102		1	M	50	

Supplementary References

1. Claros, M. G. & Vincens, P. Computational method to predict mitochondrially imported proteins and their targeting sequences. *Eur. J. Biochem.* 241, 779–786 (1996).

2. Botker, H. E., Cabrera-Fuentes, H. A., Ruiz-Meana, M., Heusch, G. & Ovize, M. Translational issues for mitoprotective agents as adjunct to reperfusion therapy in patients with ST-segment elevation myocardial infarction. *J. Cell. Mol. Med.* (2020).

3. Lin, J. J. Monoclonal antibodies against myofibrillar components of rat skeletal muscle decorate the intermediate filaments of cultured cells. *Proc. Natl. Acad. Sci. U. S. A.* 78, 2335–2339 (1981).

4. Yoon, Y., Krueger, E. W., Oswald, B. J. & McNiven, M. A. The mitochondrial protein hFis1 regulates mitochondrial fission in mammalian cells through an interaction with the dynamin-like protein DLP1. *Mol. Cell. Biol.* 23, 5409–5420 (2003).

5. De Stefani, D., Raffaello, A., Teardo, E., Szabo, I. & Rizzuto, R. A forty-kilodalton protein of the inner membrane is the mitochondrial calcium uniporter. *Nature* 476, 336–340 (2011).

6. O-Uchi, J. *et al*. Adrenergic signaling regulates mitochondrial Ca2+ uptake through Pyk2-dependent tyrosine phosphorylation of the mitochondrial Ca2+ uniporter. *Antioxid. Redox Signal.* 21, 863–879 (2014).

7. Hamilton, S. *et al*. Pharmacological Modulation of Mitochondrial Ca(2+) Content Regulates Sarcoplasmic Reticulum Ca(2+) Release via Oxidation of the Ryanodine Receptor by Mitochondria-Derived Reactive Oxygen Species. *Front. Physiol.* 9, 1831 (2018).

8. Stewart, S. A. *et al*. Lentivirus-delivered stable gene silencing by RNAi in primary cells. *RNA* 9, 493–501 (2003).

9. Carter, R. E. & Sorkin, A. Endocytosis of functional epidermal growth factor receptor-green fluorescent protein chimera. *J. Biol. Chem.* 273, 35000–35007 (1998).

10. Bertolin, G. *et al*. Mitochondrial Aurora kinase A induces mitophagy by interacting with MAP1LC3 and Prohibitin 2. *Life. Sci. Alliance* 4, e202000806. doi: 10.26508/lsa.202000806. Print 2021 Jun (2021).

11. Tait, S. W. *et al.* Resistance to caspase-independent cell death requires persistence of intact mitochondria. *Dev. Cell.* 18, 802–813 (2010).

12. Vang, A. *et al*. alpha7 Nicotinic acetylcholine receptor mediates right ventricular fibrosis and diastolic dysfunction in pulmonary hypertension. *JCI Insight* 6, 10.1172/jci.insight.142945 (2021).
