# Supplementary material for: Mitochondrial Ca2+ uniporter (MCU) variants form plasma-membrane channels
Source: Commun Biol. 2026 May 20;9:1074. doi: 10.1038/s42003-026-10285-x (PMC13458766; doi:10.1038/s42003-026-10285-x)
Supplement: Supplementary file 2 [file 42003_2026_10285_MOESM4_ESM.pdf]

nature portfolio

Corresponding author(s): Jin O-Uchi

Last updated by author(s): 03182026

Reporting Summary

Nature Portfolio wishes to improve the reproducibility of the work that we publish. This form provides structure for consistency and transparency in reporting. For further information on Nature Portfolio policies, see our Editorial Policies and the Editorial Policy Checklist.

Statistics

For all statistical analyses, confirm that the following items are present in the figure legend, table legend, main text, or Methods section.

n/a	Confirmed	
☐	☒	The exact sample size (*n*) for each experimental group/condition, given as a discrete number and unit of measurement
☐	☒	A statement on whether measurements were taken from distinct samples or whether the same sample was measured repeatedly
☐	☒	The statistical test(s) used AND whether they are one- or two-sided<br>*Only common tests should be described solely by name; describe more complex techniques in the Methods section.*
☒	☐	A description of all covariates tested
☒	☐	A description of any assumptions or corrections, such as tests of normality and adjustment for multiple comparisons
☐	☒	A full description of the statistical parameters including central tendency (e.g. means) or other basic estimates (e.g. regression coefficient) AND variation (e.g. standard deviation) or associated estimates of uncertainty (e.g. confidence intervals)
☐	☒	For null hypothesis testing, the test statistic (e.g. *F*, *t*, *r*) with confidence intervals, effect sizes, degrees of freedom and *P* value noted<br>*Give P values as exact values whenever suitable.*
☒	☐	For Bayesian analysis, information on the choice of priors and Markov chain Monte Carlo settings
☒	☐	For hierarchical and complex designs, identification of the appropriate level for tests and full reporting of outcomes
☐	☒	Estimates of effect sizes (e.g. Cohen's *d*, Pearson's *r*), indicating how they were calculated

Our web collection on statistics for biologists contains articles on many of the points above.

Software and code

Policy information about availability of computer code

Data collection	N/A
Data analysis	N/A

For manuscripts utilizing custom algorithms or software that are central to the research but not yet described in published literature, software must be made available to editors and reviewers. We strongly encourage code deposition in a community repository (e.g. GitHub). See the Nature Portfolio guidelines for submitting code & software for further information.

Data

Policy information about availability of data

All manuscripts must include a data availability statement. This statement should provide the following information, where applicable:

- Accession codes, unique identifiers, or web links for publicly available datasets
- A description of any restrictions on data availability
- For clinical datasets or third party data, please ensure that the statement adheres to our policy

All data supporting the findings of this study are available within the paper and its Supplementary Information.

Research involving human participants, their data, or biological material

Policy information about studies with human participants or human data. See also policy information about sex, gender (identity/presentation), and sexual orientation and race, ethnicity and racism.

Reporting on sex and gender	All the human platelet samples from healthy donors were obtained from commercial sources. The donors' information including age, sex, race provide from the commercial sources were shown in the supplementary information.
Reporting on race, ethnicity, or other socially relevant groupings	All the human platelet samples from healthy donors were obtained from commercial sources. The donors' information including age, sex, race provide from the commercial sources were shown in the supplementary information.
Population characteristics	N/A
Recruitment	N/A
Ethics oversight	University of Minnesota

Note that full information on the approval of the study protocol must also be provided in the manuscript.

Field-specific reporting

Please select the one below that is the best fit for your research. If you are not sure, read the appropriate sections before making your selection.

☒ Life sciences ☐ Behavioural & social sciences ☐ Ecological, evolutionary & environmental sciences

For a reference copy of the document with all sections, see nature.com/documents/nr-reporting-summary-flat.pdf

Life sciences study design

All studies must disclose on these points even when the disclosure is negative.

Sample size	We used power analysis to determine the sample size (Lenth, R. V. (2006-9). Java Applets for Power and Sample Size [Computer software] http://www.stat.uiowa.edu/~rlenth/power; as published: Chan TO et al, Proc Natl Acad Sci U S A. 2011, Circulation. 2011 Aug 2;124(5):572-81).
Data exclusions	No data were excluded from the data analysis.
Replication	The biochemical assessments were performed duplicate or triplicate tand confirmed the reproducibility.
Randomization	Cell groups and and their treatments will be randomly assigned by Research Randomizer.
Blinding	All outcome measures and analysis were performed blinded to treatment.

Reporting for specific materials, systems and methods

We require information from authors about some types of materials, experimental systems and methods used in many studies. Here, indicate whether each material, system or method listed is relevant to your study. If you are not sure if a list item applies to your research, read the appropriate section before selecting a response.

Materials & experimental systems

n/a	Involved in the study	
☐	☒	Antibodies
☐	☒	Eukaryotic cell lines
☒	☐	Palaeontology and archaeology
☐	☒	Animals and other organisms
☒	☐	Clinical data
☒	☐	Dual use research of concern
☒	☐	Plants

Methods

n/a	Involved in the study	
☒	☐	ChIP-seq
☒	☐	Flow cytometry
☒	☐	MRI-based neuroimaging

Antibodies

Antibodies used	The detailed information of all commercial antibodies used in this study, including company name and catalog numbers, was listed in

Antibodies used	the supplementary tables
Validation	The specificity of antibodies was verified by Western blot analysis with purified target proteins, peptide blocking experiments or knockout samples.

Eukaryotic cell lines

Policy information about cell lines and Sex and Gender in Research

Cell line source(s)	All the cell lines were purchased from American Type Culture Collection (Manassas, VA) except HEK293T cells and C2c12 cells. HEK293T cells were kindly provided by Dr. Keigi Fujiwara (University of Texas, MD Anderson Cancer Center, Houston, TX). C2C12 mouse myoblast cells were kindly provided by Dr. Robert T Dirksen (University of Rochester, Rockester NY).
Authentication	None of the cell line used are not authenticated.
Mycoplasma contamination	Cell lines were not tested for mycoplasma contamination.
Commonly misidentified lines (See ICLAC register)	N/A

Animals and other research organisms

Policy information about studies involving animals; ARRIVE guidelines recommended for reporting animal research, and Sex and Gender in Research

Laboratory animals	Two- to three-day-old male (Envigo, Huntingdon, UK and Taconic Biosciences, Germantown, NY) and 8-month-old male and female adult FVB mice (Taconic) and C56BL/6 mice (Jackson Laboratory) were used.
Wild animals	This study does not involve wild animals.
Reporting on sex	Both sex animals were use as a source fo primary cells.
Field-collected samples	This study does not involve field-collected samples
Ethics oversight	All animal experiments were performed in accordance with the Guidelines on Animal Experimentation of Thomas Jefferson University, Rhode Island Hospital, and University of Minnesota. The study protocols were approved by the Institutional Animal Care and Use Committee at each institution.

Note that full information on the approval of the study protocol must also be provided in the manuscript.
