# Supplementary material for: Mitochondrial Ca2+ uniporter (MCU) variants form plasma-membrane channels
Source: Commun Biol. 2026 May 20;9:1074. doi: 10.1038/s42003-026-10285-x (PMC13458766; doi:10.1038/s42003-026-10285-x)
Supplement: Supplementary file 3 [file 42003_2026_10285_MOESM5_ESM.pdf]

nature portfolio

Peer Review File

Mitochondrial Ca2+ uniporter (MCU) variants form plasma-membrane channels

Corresponding Author: Dr Jin O-Uchi

This file contains all reviewer reports in order by version, followed by all author rebuttals in order by version.

Version 0:

Reviewer comments:

Reviewer #1

(Remarks to the Author)
The manuscript provides intriguing insights into the role of MCU-S in mitochondrial calcium biology. While the findings are promising, there are several areas that could benefit from further clarification and experimental validation.
Comments:
MCU Variants and Localization: The manuscript suggests that MCU-S does not have a mitochondrial targeting sequence (MTS) and thus does not localize to the mitochondria. However, it's essential to provide more experimental evidence to support this claim, such as electron microscopy or high-resolution confocal microscopy.
Overexpression Concerns: Overexpression can sometimes lead to non-physiological interactions. It would be beneficial if the authors could provide evidence that the levels of MCU-S overexpression are within a physiologically relevant range. Address the potential for MCU-S aggregation due to overexpression using techniques like size-exclusion chromatography.
MCU-L and MCU-S Interaction: Provide more direct evidence of the MCU-L and MCU-S interaction using techniques like Förster resonance energy transfer (FRET) or bimolecular fluorescence complementation (BiFC). Investigate if this interaction occurs in cells or tissues where both proteins are endogenously expressed.
Functional Assays: Consider patch-clamp experiments or other electrophysiological techniques to provide direct evidence of the calcium channels' activity formed by MCU-S. Provide a dose-response curve for the use of YM-58483 to ensure specificity for SOCE inhibition.
Physiological Relevance: Investigate the role of MCU-S in more physiologically relevant models, such as primary cells or animal models. Explore the potential role of MCU-S in platelet function, such as aggregation or clotting.
Elucidate the mechanism by which MCU-S inhibits MCU-L trafficking to the inner mitochondrial membrane (IMM). Determine the domains of MCU-L and MCU-S involved in their interaction.
Figures and Supplementary Data: Ensure clarity and proper labeling of all figures and supplementary data. Address any anomalies or unexpected results in the discussion.
Discuss the broader implications of the findings and suggest future research directions in this area.

Reviewer #2

(Remarks to the Author)
In this work, O-Uchi and his team report that MCU-S, an isoform of the mitochondrial calcium uniporter devoid of the mitochondrial targeting sequence (MTS), a product of alternative RNA splicing, is ubiquitously expressed in human cells and tissues along with the full-length MCU, (here referred to as MCU-L). The authors find that MCU-S is targeted to the plasma membrane, where it forms Ca2+-conducting channels. The highest expression of MCU-S is found in platelets, where, as the authors propose, it contributes to the platelet activation process. Evidence is also presented that alternative splicing of the corresponding mouse gene produces an even shorter isoform (MCU-SS), missing not only MTS but also ~100 residues of the N-terminal domain of mature MCU. The manuscript is well-written and generally provides adequate experimental support for the conclusions. However, there are a few caveats that raise questions about the physiological significance of MCU-S and MCU-SS.

1. I don't think there is convincing evidence in the manuscript for the presence of MCU-S or MCU-SS proteins in wild-type cells and tissues. The detection method used here is primarily based on PCR and specific primers, which quantifies the

corresponding mRNA but does not guarantee that the respective proteins are present. An attempt at detecting these proteins is the immunoblot in Fig. 1E for the mouse MCU-SS. However, the antibody used in this experiment is not specific for MCU-SS; it cross-reacts with MCU-L. Hence, the faint 25kDa band on the immunoblot might originate from MCU-L degradation. The human MCU-S protein is technically challenging to detect (as the authors point out) due to its similarity to the mature MCU-L protein and was not confirmed. Most subsequent experiments with MCU-S presented in the manuscript are based on overexpression of appropriate plasmids in model cell lines.

2. The non-mitochondrial localization of MTS-free MCU and its capacity to reconstitute Ca2+conducting channels in the plasma membrane is very interesting and well documented in the manuscript. However, this observation is not entirely novel. A heterologous expression of a concatenated MCU-EMRE construct in Xenopus oocytes plasma membrane has been described and utilized for functional studies (PMID: 29891485).

3. It has been argued (e.g., PMID: 27712956) that the most biologically plausible alternatively spliced protein isoforms tend to have the most cross-species conservation and do not compromise functional domains. In this respect, the alternatively spliced MCU isoforms presented here are worrisome. While both human MCU-S and mouse MCU-SS comprise the transmembrane region of MCU, which is indispensable for the channel's function, there is a ~100 residue difference between them on the matrix side. Structurally, this results in the loss of the N-terminal domain in MCU-SS, potentially leading to reduced stability of the MCU-SS tetramer and precludes the characteristic dimerization of the uniporter complex evident from the cryo-EM structures (PMID: 32494073, PMID: 32762847).

4. It is unclear how the Ca2+ conductance of MCU-S channels could be regulated. There is strong evidence that the MICU1/MICU2 complex regulates the function of the uniporter in mitochondria. A similar mechanism cannot operate for the plasma membrane-localized MCU-S Ca2+ channel due to its continuous exposure to Ca2+ concentrations in the millimolar range, which is ~1000x above the Kd for Ca2+ of MICU1/MICU2.

Minor comments:

While reading the manuscript, I often wondered if the abbreviations referred to mRNAs or their protein products. It would be helpful for a reader if there were a clear distinction between the two in the text.

Supplementary Fig. 2 is supposed to show the design of the primers, but something is amiss. No MCU-S and MCU-L primers are shown. MCU-L2 primers are shown twice.

Fig. 2E. What is MCU1-Flag?

Page 5, Fig. 3B, and Supplementary Fig. 6. The authors argue that overexpression of MCU-S redirects the MCU-L into the plasma membrane, where the two isoforms form a heterocomplex. The result is the appearance of unprocessed full-length MCU-L (including MTS) in the cytosolic fraction, a decrease of the mature MCU-L in the mitochondrial fraction, and a loss of mitochondrial Ca2+ uptake (Fig. 3B). While the presence of MCU-S and MCU-L in the cytosolic fraction is visible, I don't see any evidence for the incorporation of MCU-S into the mitochondria (the case of MCU-L > MCU-S in part C).

Fig. 4, D,E,F. It is unclear why there is no Ru360 sensitivity in panels E and F. A clarification in the text is needed.

Version 1:

Reviewer comments:

Reviewer #2

(Remarks to the Author)
The authors addressed the reviewers' comments satisfactorily. The experiments are expertly executed, and the data present convincing arguments that a short form of MCU devoid of the mitochondria-targeting sequence can form Ca2+-permeable channels at the plasma membrane of platelets. Lingering questions remain with respect to the potential mechanism of regulation of such channels and their physiological relevance. Hopefully, these questions will be addressed in future studies.

Open Access This Peer Review File is licensed under a Creative Commons Attribution 4.0 International License, which permits use, sharing, adaptation, distribution and reproduction in any medium or format, as long as you give appropriate credit to the original author(s) and the source, provide a link to the Creative Commons license, and indicate if changes were made.
In cases where reviewers are anonymous, credit should be given to 'Anonymous Referee' and the source.
The images or other third party material in this Peer Review File are included in the article's Creative Commons license, unless indicated otherwise in a credit line to the material. If material is not included in the article's Creative Commons license and your intended use is not permitted by statutory regulation or exceeds the permitted use, you will need to obtain permission directly from the copyright holder.
To view a copy of this license, visit https://creativecommons.org/licenses/by/4.0/

We appreciate the constructive comments from the reviewers and editor. Following the reviewers' suggestions, we performed additional experiments and extensively revised our manuscript. All the revised and additional texts in the updated manuscript were shown in red. Our new data sets were shown in Fig. 2D and E, Fig. 4I and J, Table 1, and Supplementary Figs. 2, 5, 7, 8, and 10.

Reviewer #1

1-1) MCU Variants and Localization: The manuscript suggests that MCU-S does not have a mitochondrial targeting sequence (MTS) and thus does not localize to the mitochondria. However, it's essential to provide more experimental evidence to support this claim, such as electron microscopy or high-resolution confocal microscopy.

>> Following the reviewer's suggestions, we quantified the plasma membrane (PM) localization of MCU-S and MCU-SS using confocal microscopy (see Fig. 2D and E, page 4 line 37 to 38 in the revised manuscript. The materials and methods corresponding to this additional experiment were added to the revised supplementary file).

1-2) Overexpression Concerns: Overexpression can sometimes lead to non-physiological interactions. It would be beneficial if the authors could provide evidence that the levels of MCU-S overexpression are within a physiologically relevant range.

>> We agree with the reviewer and confirmed that MCU-S overexpression levels in the MEG-01 cells are comparable to those in the human platelets (see Fig. 4J, page 6 line 22 to 23 in the revised manuscript. The materials and methods corresponding to this additional experiment were added to the revised supplementary file).

1-3) Address the potential for MCU-S aggregation due to overexpression using techniques like size-exclusion chromatography.

>> Following the reviewer`s suggestions, we performed additional experiments to assess the potential aggregation via MCU constructs by staining the cells with an aggresome-detectable dye [1]. We confirmed that the MCU constructs used in this study did not significantly induce protein aggregations in the transfected cells (see Supplementary Fig. 7, and page 4 line 40 to 42 in the revised manuscript. The materials and methods corresponding to this additional experiment were added to the revised supplementary file).

1-4) MCU-L and MCU-S Interaction: Provide more direct evidence of the MCU-L and MCU-S interaction using techniques like Förster resonance energy transfer (FRET) or bimolecular fluorescence complementation (BiFC). Investigate if this interaction occurs in cells or tissues where both proteins are endogenously expressed.

>> Following the reviewer`s suggestions, we performed additional experiments using FRET in live cells [2] and confirmed MCU-L-MCU-S and MCU-L-MCU-SS interactions (see Supplementary Fig. 10 and page 5 line 21 to 23 in the revised manuscript The materials and methods corresponding to this additional experiment was added to the revised supplementary file).

To assess the endogenous MCU-L-MCU-S interaction in cells/tissues, it is required to have variant-specific antibodies. However, as reviewer 2 mentioned (see comment 2-1), developing the variant-specific antibodies is technically challenging due to the high similarity of the mature MCU-L and MCU-S proteins (only one amino acid difference on the N-terminal, see

also Supplementary Fig. 1). We added this information to the result and discussion sections in the revised manuscript as a key limitation of this study (see page 4 line 11 to 14, and page 8 line 43 to 45 in the revised manuscript).

1-5) Functional Assays: Consider patch-clamp experiments or other electrophysiological techniques to provide direct evidence of the calcium channels' activity formed by MCU-S.

>> As we provided the data set in the initial submission, the electrophysiological detection of the MCU-S channel activity using a whole-cell patch clamp technique is shown in Fig. 2L-N (see also page 5 line 7 to 14 in the revised manuscript).

1-6) Provide a dose-response curve for the use of YM-58483 to ensure specificity for SOCE inhibition.

>> A concentration-dependent effect of YM-58483 on SOCE and the effect of YM-58483 on non-capacitive Ca^{2+} entry in human platelets were reported by Harper M et al [3]. We selected the concentration and treatment time that can significantly block SOCE but not non-capacitive Ca^{2+} entry (see page 6 line 3 to 7 in the revised manuscript).

1-7) Physiological Relevance: Investigate the role of MCU-S in more physiologically relevant models, such as primary cells or animal models. Explore the potential role of MCU-S in platelet function, such as aggregation or clotting.

>> Following the reviewer's suggestion, we showed the effect of MCU blocker Ru360 on human platelet aggregations using a microtiter plate-based light transmission aggregometry [4, 5] (see Fig. 4I, page 5 line 46, page 6 line 9, page 8 line 21 to 22 in the revised manuscript. The materials and methods corresponding to this additional experiment were added to the revised supplementary file).

1-8) Elucidate the mechanism by which MCU-S inhibits MCU-L trafficking to the inner mitochondrial membrane (IMM). Determine the domains of MCU-L and MCU-S involved in their interaction.

>> Since matured MCU-S and MCU-L has only one amino acid difference at the N-terminal (Supplementary Fig.1), it is feasible that MCU-S and MCU-L can form hetero-oligomers by the similar domain interactions found in MCU-L-MCU-L or MCU-L-MCUb [6, 7], which contributes the extra-mitochondrial retention of MCU heteromers under MCU-S overexpression (Fig. 3 and Supplementary Fig. 11). We added this to the discussion on page 7 line 14 to 19 in the revised manuscript.

1-9) Figures and Supplementary Data: Ensure clarity and proper labeling of all figures and supplementary data.

>> We ensured proper labeling of all the main figures and supplementary data in the revised manuscript.

1-10) Address any anomalies or unexpected results in the discussion. Discuss the broader implications of the findings and suggest future research directions in this area.

>> We addressed the detection of EMRE from the PM of human platelets as an unexpected result and further discussed any potential discrepancy with other data we collected in this study (see page 7 line 25 to 31, and line 32 to 41).

Moreover, following the reviewer's suggestion, we added to the discussion the broader implications of the findings and provided future research directions in this area (see page 8 line 48 to page 9 line 3).

Reviewer #2:

2-1) I don't think there is convincing evidence in the manuscript for the presence of MCU-S or MCU-SS proteins in wild-type cells and tissues. The detection method used here is primarily based on PCR and specific primers, which quantifies the corresponding mRNA but does not guarantee that the respective proteins are present. An attempt at detecting these proteins is the immunoblot in Fig. 1E for the mouse MCU-SS. However, the antibody used in this experiment is not specific for MCU-SS; it cross-reacts with MCU-L. Hence, the faint 25kDa band on the immunoblot might originate from MCU-L degradation. The human MCU-S protein is technically challenging to detect (as the authors point out) due to its similarity to the mature MCU-L protein and was not confirmed. Most subsequent experiments with MCU-S presented in the manuscript are based on overexpression of appropriate plasmids in model cell lines.

>> The reviewer is correct. There are several technical challenges in detecting endogenous MCU variant proteins, which include that it is nearly impossible to design a short MCU variant specific antibody due to their structural similarity with MCU-L (see page 4 line 11 to 14, and page 8 line 46 to 48 in the revised manuscript).

As the reviewer mentioned, the 25-kDa band in the C2C12 was faint (Fig. 1E), possibly due to the endogenous *MCU-SS* expression level in this cell line. Therefore, following the reviewer's suggestions, we detected the endogenous MCU-SS with more prominent 25-kDa band in the protein lysates from mouse platelets (Supplementary Fig. 5 and page 4 line 14 to 18, and line 20-21). We also emphasize the technical challenges in detecting endogenous MCU variant proteins as one of the "limitations of this study" in the discussion section (see page 8 line 46 to 48 in the revised manuscript).

2-2) The non-mitochondrial localization of MTS-free MCU and its capacity to reconstitute Ca^{2+} conducting channels in the plasma membrane is very interesting and well documented in the manuscript. However, this observation is not entirely novel. A heterologous expression of a concatenated MCU-EMRE construct in Xenopus oocytes plasma membrane has been described and utilized for functional studies (PMID: 29891485).

>> Thank you for this comment about PMID: 29891485 [8] which was the first report showing the detection of MCU channel activity at the PM using the heterologous Xenopus oocyte expression system. We included this reference [8] in our discussion section (see page 7 line 22 to line 24, and line 35 to 38).

2-3) It has been argued (e.g., PMID: 27712956) that the most biologically plausible alternatively spliced protein isoforms tend to have the most cross-species conservation and do not compromise functional domains. In this respect, the alternatively spliced MCU

isoforms presented here are worrisome. While both human MCU-S and mouse MCU-SS comprise the transmembrane region of MCU, which is indispensable for the channel's function, there is a ~100 residue difference between them on the matrix side. Structurally, this results in the loss of the N-terminal domain in MCU-SS, potentially leading to reduced stability of the MCU-SS tetramer and precludes the characteristic dimerization of the uniporter complex evident from the cryo-EM structures (PMID: 32494073, PMID: 32762847).

>> MCU-S and MCU-SS, which are the focus of this manuscript, are <u>not splicing variants but variants from alternative promoters</u>. Since the use of alternative promoter is associated with various diseases[9], and organ development [10], exploring the MCU-S and MCU-SS expression has the potential to provide new insights into the regulatory complexity of MCU functions in human physiology and pathophysiology (see also page 8 line 48 to 51).

As discussed in PMID: 27712956, a large majority of predicted alternative splicing variants may not exist or not be translated into proteins, which is consistent with a part of our results in that the predicted alternative splicing variant *MCU-L2* was barely datable in all cell types we tested (see Fig. 1 and Supplementary Fig. 4). We agree that most human genes have a single main protein isoform, while the alternative isoforms that are identified tend to be the most biologically plausible (such as those with the most cross-species conservation and those that do not compromise functional domains). Therefore, as the reviewer commented, careful assessment is needed to test the functions of the alternatively spliced variants.

We also agree that there is significant structural difference in the N-terminal region of MCU-S and MCU-SS, which warrants further discussion. Therefore, we expanded our discussion to include whether MCU-SS form stable functional channel despite we detected Ca^{2+}-permeability at the PM after MCU-SS overexpression (see Supplementary Fig. 8), and how the N-terminal deletion in MCU-SS may lead to functional difference between MCU-S or MCU-L (see page 8 line 1 to 16).

2-4) It is unclear how the Ca2+ conductance of MCU-S channels could be regulated. There is strong evidence that the MICU1/MICU2 complex regulates the function of the uniporter in mitochondria. A similar mechanism cannot operate for the plasma membrane-localized MCU-S Ca2+ channel due to its continuous exposure to Ca2+ concentrations in the millimolar range, which is ~1000x above the Kd for Ca2+ of MICU1/MICU2.

>> We agree with the reviewer that similar mechanism of MICU1/MICU2 regulation in MCU-L is unlikely to be appreciable to PM-localized MCU-S channels operating for the plasma membrane-localized MCU-S Ca^{2+} channel due to its continuous exposure to Ca^{2+} concentrations in the millimolar range, which is ~1000x above the K_d for Ca^{2+} of MICU1/MICU2. Giving that $I_{Ca,MCU}$ currents were recorded without reconstitution of MICU1/MICU2 in our HEK293T heterologous system and in the Xenopus oocyte expression system by Tsai`s group [8], we explored the possibility that MCU-S may not receive the MICU1/MICU2 regulation and added this to the discussion in our revised manuscript (see page 7 line 32 to 41, and line 46 to 48). Since the interaction between the MICU subunits and MCU-L is suggested to occur via EMRE [11], MCU-S at the PM may not interact with MICU subunits due the lack of (or low expression of) EMRE at the PM in human platelets.

Minor comments:

1. While reading the manuscript, I often wondered if the abbreviations referred to mRNAs or their protein products. It would be helpful for a reader if there were a clear distinction between the two in the text.

>> In the revised manuscript, we now use *italic text* for all mRNAs (e.g., *MCU-L*) and non-italic for proteins (e.g., MCU-L) and also specify whether abbreviations refer to mRNA (gene) or protein. We also provide a new able 1 summarizing all abbreviations related to MCU variants used in this study.

2. Supplementary Fig. 2 is supposed to show the design of the primers, but something is amiss. No MCU-S and MCU-L primers are shown. MCU-L2 primers are shown twice.

>> We revised Supplementary Fig. 2 and now provide the information for the primers specific to all human *MCU* (A) and mouse *Mcu* variants.

3. Fig. 2E. What is MCU1-Flag?
>>We replaced "MCU1-Flag" with "MCU-L-Flag" (see Fig. 2G in the revised manuscript).

4. Page 5, Fig. 3B, and Supplementary Fig. 6. The authors argue that overexpression of MCU-S redirects the MCU-L into the plasma membrane, where the two isoforms form a heterocomplex. The result is the appearance of unprocessed full-length MCU-L (including MTS) in the cytosolic fraction, a decrease of the mature MCU-L in the mitochondrial fraction, and a loss of mitochondrial Ca2+ uptake (Fig. 3B). While the presence of MCU-S and MCU-L in the cytosolic fraction is visible, I don't see any evidence for the incorporation of MCU-S into the mitochondria (the case of MCU-L > MCU-S in part C).

>> In Fig. 2A to C, we reintroduced MCU-SS or -SS-GFP MCU knock-down (MCU-KD) cells (i.e., HEK293T cell line stably overexpressing shRNA plasmids targeting the 3'-UTR of *MCU* mRNA[12]), where small amount of residual MCU-L still exists endogenously. In this condition, MCU-S and MCU-SS showed significantly lower co-localization with mt-RFP compared to MCU-L, indicating that these isoforms are distributed at extramitochondrial sites. However, a small fraction is still detectable in the mitochondria if there are a small amount of residual MCU-L co-existed (see page 4 line 34 to 36).

In addition, a new data set is shown in Supplementary Fig. 10. When MCU-S was co-overexpressed in HEK293T cells with a mitochondrial protein Smac-mCherry, they are significantly co-localized (but FRET between GFP and mCherry was low), suggesting that exogenous MCU-S is mostly transferred to mitochondria when endogenous MCU-L exists.

5. Fig. 4 D,E,F. It is unclear why there is no Ru360 sensitivity in panels E and F. A clarification in the text is needed.

>> Following the reviewer's suggestions, we now provide graph legends within panels D, E, and F in Fig 4 to show more clearly that E and F include the Ru360 sensitivity experiments.

References

1. Ma, M. T. *et al*. Antibody-mediated clearance of an ER-resident aggregate that causes glaucoma. *PNAS Nexus* **4**, pgae556 (2024).

2. Bertolin, G. *et al*. Mitochondrial Aurora kinase A induces mitophagy by interacting with MAP1LC3 and Prohibitin 2. *Life. Sci. Alliance* **4**, e202000806. doi: 10.26508/lsa.202000806. Print 2021 Jun (2021).

3. Harper, M. T. & Poole, A. W. Store-operated calcium entry and non-capacitative calcium entry have distinct roles in thrombin-induced calcium signalling in human platelets. *Cell Calcium* **50**, 351–358 (2011).

4. Tamang, H. K., Stringham, E. N. & Tourdot, B. E. Platelet Functional Testing Via High-Throughput Microtiter Plate-Based Assays. *Curr. Protoc.* **3**, e668 (2023).

5. Chan, M. V., Armstrong, P. C. & Warner, T. D. 96-Well Plate-Based Aggregometry. *Platelets* **29**, 650–655 (2018).

6. Noble, M., Colussi, D. M., Junop, M. & Stathopulos, P. B. The MCU and MCUb amino-terminal domains tightly interact: mechanisms for low conductance assembly of the mitochondrial calcium uniporter complex. *iScience* **27**, 109699 (2024).

7. Zhuo, W. *et al*. Structure of intact human MCU supercomplex with the auxiliary MICU subunits. *Protein Cell.* **12**, 220–229 (2021).

8. Tsai, C. & Tsai, M. Electrical recordings of the mitochondrial calcium uniporter in Xenopus oocytes. *J. Gen. Physiol.* **150**, 1035–1043 (2018).

9. Vacik, T. & Raska, I. Alternative intronic promoters in development and disease. *Protoplasma* **254**, 1201–1206 (2017).

10. Tan, J. & Sun, Y. Alternative promoter usage during organ development. *PLoS Genet.* **21**, e1011635 (2025).

11. Sancak, Y. *et al*. EMRE is an essential component of the mitochondrial calcium uniporter complex. *Science* **342**, 1379–1382 (2013).

12. Baughman, J. M. *et al*. Integrative genomics identifies MCU as an essential component of the mitochondrial calcium uniporter. *Nature* **476**, 341–345 (2011).
