# Supplementary material for: Mitochondrial Ca2+ uniporter (MCU) variants form plasma-membrane channels
Source: Commun Biol. 2026 May 20;9:1074. doi: 10.1038/s42003-026-10285-x (PMC13458766; doi:10.1038/s42003-026-10285-x)
Supplement: Supplementary file 4 [file 42003_2026_10285_MOESM2_ESM.docx]

**Description of Additional Supplementary Files**

**File name:** Supplementary Data 1

**Description:** All source data underlying the graphs presented in the main figures and the supplementary figures
